# An Efficient Synthesis of Aryl-Substituted Pyrroles by the Suzuki–Miyaura Coupling Reaction of SEM-Protected Pyrroles

**DOI:** 10.3390/molecules24081594

**Published:** 2019-04-22

**Authors:** Keli Cui, Meng Gao, Hongyi Zhao, Dongfeng Zhang, Hong Yan, Haihong Huang

**Affiliations:** 1 College of Life Science and Bio-engineering, Beijing University of Technology, 100 Ping Le Yuan, Chaoyang District, Beijing 100124, China; ckl0615@163.com; 2 State Key Laboratory of Bioactive Substances and Function of Natural Medicine, Beijing Key Laboratory of Active Substance Discovery and Druggability Evaluation, Institute of Materia Medica, Peking Union Medical College and Chinese Academy of Medical Sciences, 1 Xian Nong Tan Street, Beijing 100050, China; gaomengss@imm.ac.cn (M.G.); zhaohongyicool@imm.ac.cn (H.Z.)

**Keywords:** aryl-substituted pyrroles, Suzuki–Miyaura coupling reaction, SEM-protected pyrroles

## Abstract

An efficient arylation of SEM-protected pyrroles by the Suzuki–Miyaura coupling reaction has been developed. The reaction can be carried out under mild conditions to provide aryl-substituted pyrroles in moderate to excellent yields. The scope and limitations of the methodology were evaluated, and the reaction was tolerant of a wide range of functionalities. Compared to the reported methods, the protocol has some advantages, such as commercially available materials, no debrominated by-products being formed, and the amine-protecting group being stable under the reaction conditions. The synthetic utility of the product has also been demonstrated, with several common transformations of the aryl-substituted pyrrole product being conducted. This protocol will offer the opportunity to explore other metal-catalyzed cross-coupling reactions employing SEM-protected pyrroles.

## 1. Introduction

Aryl-substituted pyrroles are an important structural motif in both pharmaceuticals and natural products alike, and display a wide range of interesting biological activities [1]. Aryl pyrroles bearing an ester functional group are commonly used in organic synthesis and medicinal chemistry as synthetic intermediates [2,3,4,5,6]. There are a great deal of methods for the preparation of aryl-substituted pyrroles with this pendant ester group, and generally, the pyrrole core is formed from functionalized precursors with complex structures employing various catalysts and ligands [7,8,9,10,11,12,13,14]. As our aim was to construct a small library of aryl-substituted pyrroles for biological activity assays, we found that it was inefficient to build these required pyrrole structures from some non-commercial starting materials. If the aryl-substituted pyrroles could be synthesized from commercially available pyrrole compounds, the approach will be simple, straightforward, and efficient. The coupling reaction is a powerful method in the formation of C(sp_2_)–C(sp_2_) bonds, and the aryl-substituted pyrroles could be synthesized by the Stille–Migita cross-coupling reaction [15,16], decarboxylative coupling reaction [16,17], metal-catalyzed desulfitative coupling reaction [18], and Suzuki–Miyaura coupling reaction [16]. Considering the availability of materials and the operability of the reaction, the Suzuki–Miyaura coupling reaction may be the most suitable method to prepare aryl pyrroles from arylboronic acids and bromopyrroles. However, without protection of the nitrogen of the pyrrole moiety, debromination readily occurs, and the debrominated compounds are generally obtained in a large percentage [19,20]. A *t*-butyloxy carbonyl (BOC) group is the traditional choice for an amine-protecting group; however, it is well known that these groups are often unstable under general Suzuki–Miyaura coupling reaction conditions [3,19,21]. Therefore, the development of a high-throughput synthesis of aryl-substituted pyrroles with protection for subsequent application via a Suzuki–Miyaura coupling reaction is highly desirable. SEM (2-(trimethylsilyl)ethoxymethyl) represents a versatile and robust protecting group for nitrogen heterocycles that can readily be removed under a variety of deprotection conditions [22,23,24,25,26,27]. Herein, we present a method that allows for the efficient arylation of SEM-protected pyrroles via the Suzuki–Miyaura coupling reaction. This methodology provides the desired products in high yields for a wide range of substrates.

## 2. Results and Discussion

Initially, phenylboronic acid (**1a**) and 4-bromopyrrole (**2a**) were chosen as the model substrates for the Suzuki–Miyaura coupling reaction. In the presence of Pd(PPh_3_)_4_ (0.1 equiv.) and Na_2_CO_3_ (2 equiv.), the reaction between **1a** and **2a** in dioxane/H_2_O (4:1) at 90 °C afforded the desired coupling product (**3a**) in 61% yield (Table 1, entry 1). However, the use of Pd^(II)^ salts, such as Pd(PPh_3_)_2_Cl_2_, Pd(AcO)_2_, and Pd(dppf)Cl_2 ­_as the catalysts reduced the yield of the product (Table 1, entries 2–4). Although the temperature was increased to 110 °C, almost no desired product was gained in DMF (*N*,*N*-dimethyl formamide) (Table 1, entry 5). We observed that the main by-product of the reaction was the homocoupling of the arylboronic acid. Therefore, we increased the amount of **1a** to two equivalents; however, the product **3a** was obtained in almost the same yield (Table 1, entry 6 versus entry 1). Moreover, increasing the amount of Na_2_CO_3_ (6 equiv.) did not lead to a discernible effect on the yield (Table 1, entry 7 versus entry 1). We further optimized the reaction conditions by reducing the amount of H_2_O, but the product was obtained in only 5% yield (Table 1, entry 8). For further improvement of the reaction, other bases such as K_2_CO_3_, KF, and Cs_2_CO_3_ were also examined (Table 1, entries 9–11). To our delight, the product was obtained in 85% yield with Cs_2_CO_3_ as the base. Furthermore, we observed that an increase or reduction of the reaction temperature were both unfavorable for the transformation (Table 1, entries 12 and 13). When the amount of Pd(PPh_3_)_4_ was reduced to 5 mol%, the product **3a** was obtained in a slightly lower yield (Table 1, entry 14). From the above-mentioned results, it was concluded that the optimized conditions for this Suzuki–Miyaura coupling reaction protocol were 10 mol% Pd(PPh_3_)_4_, Cs_2_CO_3_ (2 equiv.) in dioxane/H_2_O (4:1) at 90 °C (Table 1, entry 11); especially, we have observed no dehalogenation in the reaction. To assess the effect of SEM-protected and BOC-protected pyrroles, BOC-protected pyrrole (**2a’**) was selected as the substrate. The reaction gave the product **3a** in good yield, but the deprotected by-product (**4a**) was obtained in 5% yield (Table 1, entry 15). The result was confirmed with the assay conducted using Na_2_CO_3_ as the base, which led to **4a** in 11% yield (Table 1, entry 16). In conclusion, the SEM-protecting group was stable under the optimal reaction conditions compared to the BOC protecting group, and a cross-coupling product was obtained in very good yield.

**Scheme 1:**
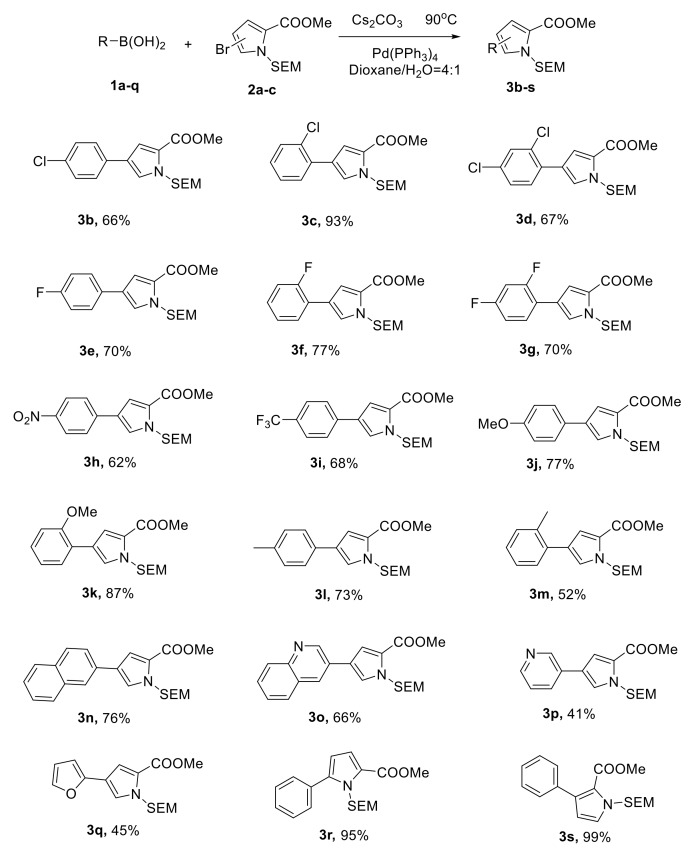
Scope of the Suzuki coupling reaction. Yields refer to the isolated yields.

With these conditions in hand, we next evaluated the substrate scope of the reaction (Scheme 1). Overall, it was found that all the attempted phenylboronic acid analogues produced the desired aryl-substituted pyrroles in moderate to excellent yields. To evaluate the effect of boronic acid substituents, we first studied the reaction of the substrate bearing the electron-withdrawing groups such as Cl, F, NO_2_, and CF_3_ (**3b**−**i**). When phenylboronic acid with Cl and F substituents were used in the ortho position, the target compounds (**3c** and **3f**) were isolated in 93% and 77% yield, respectively. In contrast, para substitution resulted in a decreased yield (**3b** and **3e**). The same behavior was observed with the 2,4-dichloro or 2,4-difluro phenylboronic acid (**3d** and **3g**). The presence of the strong electron-withdrawing NO_2_ and CF_3_ groups on the phenylboronic acid did not drastically affect the efficiency of the reaction (**3h** and **3i**).

Then, we focused our attention on the reactivity of the phenylboronic acid with the electron-donating groups. When the phenylboronic acid was substituted with an OMe group at the C-4 position, the reaction gave the product **3j** in 77% yield, while the OMe in the ortho position afforded the desired product in 87% yield (**3k**). The results displayed that this reaction followed the same behavior as the cross-coupling reaction compared to 2-fluorophenylboronic acid and 2-chlorophenylboronic acid as the substrate. Nevertheless, the Me group in the ortho position of phenylboronic acid lowered the efficiency of the reaction (**3l** versus **3m**). The reaction with the fused aromatic compounds gave the corresponding products in good yields as well (**3n** and **3o**). Notably, the reaction can also be carried out successfully with the heterocyclic substrates to furnish the desired products (**3p** and **3q**). Finally, 5-bromopyrrole and 3-bromopyrrole (**2b** and **2c**) were chosen as the substrates to document the potential of the method. The reactions worked very smoothly, giving the corresponding products in excellent yields (**3r** and **3s**).

**Scheme 2:**
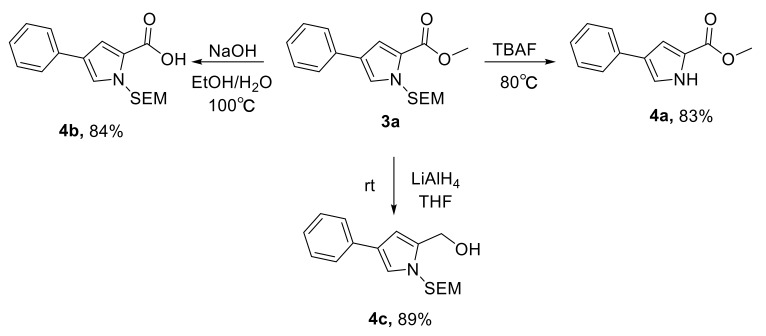
The application of SEM-protected pyrroles. Yields refer to the isolated yields.

To further explore this synthetic potential, several common transformations of the product **3a** were conducted (Scheme 2). First, the SEM-protecting group was removed smoothly in the presence of TBAF (tetrabutyl ammonium fluoride) to provide the *N*-*H*-4-phenyl pyrrole **4a** in 83% yield. Hydrolysis and reduction reactions readily occurred, leading to the fascinating pyrrole-2-carboxylic acid **4b** and 2-hydroxymethyl substituted pyrrole compound **4c** in good yields, respectively. No SEM deprotection of pyrrole was observed in the aforementioned hydrolysis and reduction reactions as anticipated.

## 3. Materials and Methods

### 3.1. General Methods

All the starting materials were obtained from commercial suppliers and were used without further purification unless stated otherwise. Reactions were monitored by Thin Layer Chromatography (silica gel GF254). Products were purified by column chromatography on silica gel (300–400 mesh). ^1^H and ^13^C spectra were recorded on a Varian (Palo Alto, CA, USA) 400 NMR or 500 NMR spectrometer using DMSO-*d*_6_ as a solvent and tetramethylsilane (TMS) as an internal standard. Chemical shifts (δ) are reported in parts per million (ppm), and coupling constants (*J*) are reported in Hertz (Hz). Data are represented as follows: chemical shift, multiplicity (br = broad, s = singlet, d = doublet, t = triplet, q = quartet, m = multiplet), coupling constants in Hertz (Hz), and integration. All the melting points were measured with a microscope melting point apparatus (MP-J3, Yanaco, Kyoto, Japan) and were uncorrected. High-resolution mass spectra were determined on ThermoExactive Orbitrap plus mass spectrometer (Waltham, MA, USA). ^1^H-NMR and HRMS spectra are available in Supplementary Materials.

### 3.2. Typical Procedure to Synthesize Compounds ***2a**–**c***

First, *t*-BuOK (330 mg, 2.94 mmol) was added to a solution of bromopyrroles (500 mg, 2.45 mmol) in anhydrous DMF (5 mL) in an ice-bath, and the mixture was stirred for 0.5 h. SEM-Cl (449 mg, 2.7 mmol) was added to the resulting mixture. The reaction mixture was stirred at rt for 2 h, and then quenched with H_2_O. The mixture was extracted with EtOAc (15 mL × 3), and the combined organic layer was washed with sat. NaHCO_3_ and brine, dried with Na_2_SO_4_, and concentrated. The product was obtained by flash chromatography (EtOAc in PE = 3%).

*Methyl 4-bromo-1-((2-(trimethylsilyl)ethoxy)methyl)-1H-pyrrole-2-carboxylate* (**2a**). Colorless oil, 71% yield. ^1^H-NMR (400 MHz, DMSO-*d_6_*) δ: 7.50 (d, *J* = 2.0 Hz, 1H, Pyrrole-5H), 6.94 (d, *J* = 2.0 Hz, 1H, Pyrrole-3H), 5.58 (s, 2H, NCH_2_O), 3.74 (s, 3H, CH_3_), 3.46 (t, *J* = 8.0 Hz, 2H, O*CH_2_*CH_2_Si), 0.79 (t, *J* = 8.0 Hz, 2H, OCH_2_*CH_2_*Si), −0.07 (s, 9H, Si(CH_3_)_3_). HRMS (ESI): *m/z* [M + H]^+^ calcd for C_12_H_21_BrNO_3_Si: 334.0469; found: 334.0448.

*Methyl 5-bromo-1-((2-(trimethylsilyl)ethoxy)methyl)-1H-pyrrole-2-carboxylate* (**2b**). Colorless oil, 96% yield. ^1^H-NMR (400 MHz, DMSO-*d_6_*) δ: 6.99 (d, *J* = 4.0 Hz, 1H, Pyrrole-3H), 6.40 (d, *J* = 4.0 Hz, 1H, Pyrrole-4H), 5.71 (s, 2H, NCH_2_O), 3.75 (s, 3H, CH_3_), 3.51 (t, *J* = 8.0 Hz, 2H, O*CH_2_*CH_2_Si), 0.80 (t, *J* = 8.0 Hz, 2H, OCH_2_*CH_2_*Si), −0.07 (s, 9H, Si(CH_3_)_3_). HRMS (ESI): *m/z* [M + H]^+^ calcd for C_12_H_21_BrNO_3_Si: 334.0469; found: 334.0463.

*Methyl 3-bromo-1-((2-(trimethylsilyl)ethoxy)methyl)-1H-pyrrole-2-carboxylate* (**2c**). Colorless oil, 81% yield. ^1^H-NMR (500 MHz, DMSO-*d_6_*) δ: 7.33 (d, *J* = 2.5 Hz, 1H, Pyrrole-5H), 6.35 (d, *J* = 2.5 Hz, 1H, Pyrrole-4H), 5.57 (s, 2H, NCH_2_O), 3.78 (s, 3H, CH_3_), 3.41 (t, *J* = 7.5 Hz, 2H, O*CH_2_*CH_2_Si), 0.78 (t, *J* = 7.5 Hz, 2H, OCH_2_*CH_2_*Si), −0.08 (s, 9H, Si(CH_3_)_3_). HRMS (ESI): *m/z* [M + H]^+^ calcd for C_12_H_21_BrNO_3_Si: 334.0469; found: 334.0460.

### 3.3. Typical Procedure to Synthesize Products ***3a**–**s***

Dioxane (8 mL) and H_2_O (2 mL) were added to the mixture of **1a**–**q** (1.5 mmol), **2a**–**c** (1 mmol), Cs_2_CO_3_ (652 mg, 2 mmol), and Pd(PPh_3_)_4_ (116 mg, 0.1 mmol) under Ar_2_ atmosphere. The reaction mixture was stirred at 90 °C for about 5 h (monitored by Thin Layer Chromatography) and cooled to rt. Then, the mixture was filtered to remove the solids, and the filtrate was concentrated. Purification by flash chromatography (EtOAc in PE = 3%) gave the desired product.

*Methyl 4-phenyl-1-((2-(trimethylsilyl)ethoxy)methyl)-1H-pyrrole-2-carboxylate* (**3a**). Colorless oil, 85% yield. ^1^H-NMR (400 MHz, DMSO-*d_6_*) δ: 7.80 (d, *J* = 1.2 Hz, 1H, Pyrrole-5H), 7.61 (d, *J* = 8.0 Hz, 2H, Ar-2,6H), 7.36–7.32 (m, 3H, Ar-3,5H, Pyrrole-3H), 7.19 (t, *J* = 8.0 Hz, 1H, Ar-4H), 5.64 (s, 2H, NCH_2_O), 3.77 (s, 3H, CH_3_), 3.50 (t, *J* = 7.6 Hz, 2H, O*CH_2_*CH_2_Si), 0.82 (t, *J* = 7.6 Hz, 2H, OCH_2_*CH_2_*Si), −0.07 (s, 9H, Si(CH_3_)_3_). ^13^C-NMR (100 MHz, DMSO-*d_6_*) δ: 161.0, 134.2, 129.2, 127.4, 126.6, 125.2, 123.9, 122.7, 116.1, 76.9, 65.6, 51.6, 17.6, −1.0. HRMS (ESI): *m/z* [M + H]^+^ calcd for C_18_H_26_NO_3_Si: 332.1677; found: 332.1662.

*Methyl 4-(4-chlorophenyl)-1-((2-(trimethylsilyl)ethoxy)methyl)-1H-pyrrole-2-carboxylate* (**3b**). Colorless oil, 66% yield. ^1^H-NMR (500 MHz, DMSO-*d*_6_) δ: 7.84 (s, 1H, Pyrrole-5H), 7.65 (d, *J* = 8.5 Hz, 2H, Ar-2,6H), 7.39 (d, *J* = 8.5 Hz, 2H, Ar-3,5H), 7.35 (s, 1H, Pyrrole-3H), 5.64 (s, 2H, NCH_2_O), 3.77 (s, 3H, CH_3_), 3.50 (t, *J* = 8.0 Hz, 2H, O*CH_2_*CH_2_Si), 0.82 (t, *J* = 8.0 Hz, 2H, OCH_2_*CH_2_*Si), −0.07 (s, 9H, Si(CH_3_)_3_). ^13^C-NMR (100 MHz, DMSO-*d_6_*) δ: 160.9, 133.2, 130.9, 129.2, 127.7, 126.9, 122.9, 122.6, 116.2, 76.9, 65.6, 51.7, 17.5, −1.0. HRMS (ESI): *m/z* [M + H]^+^ calcd for C_18_H_25_ClNO_3_Si: 366.1287; found: 366.1271.

*Methyl 4-(2-chlorophenyl)-1-((2-(trimethylsilyl)ethoxy)methyl)-1H-pyrrole-2-carboxylate* (**3c**). Colorless oil, 93% yield. ^1^H-NMR (500 MHz, DMSO-*d*_6_) δ: 7.75 (s, 1H, Pyrrole-5H), 7.56 (d, *J* = 7.5 Hz, 1H, Ar-3H), 7.50 (d, *J* = 7.5 Hz, 1H, Ar-6H), 7.36 (t, *J* = 7.0 Hz, 1H, Ar-5H), 7.29–7.27 (m, 2H, Ar-4H, Pyrrole-3H), 5.68 (s, 2H, NCH_2_O), 3.77 (s, 3H, CH_3_), 3.52 (t, *J* = 7.5 Hz, 2H, O*CH_2_*CH_2_Si), 0.82 (t, *J* = 7.5 Hz, 2H, OCH_2_*CH_2_*Si), −0.07 (s, 9H, Si(CH_3_)_3_). ^13^C-NMR (100 MHz, DMSO-*d_6_*) δ: 160.9, 133.0, 131.1, 130.7, 130.0, 128.5, 128.0, 121.9, 120.8, 119.1, 77.0, 65.6, 51.7, 17.6, −1.0. HRMS (ESI): *m/z* [M + H]^+^ calcd for C_18_H_25_ClNO_3_Si: 366.1287; found: 366.1274.

*Methyl 4-(2,4-dichlorophenyl)-1-((2-(trimethylsilyl)ethoxy)methyl)-1H-pyrrole-2-carboxylate* (**3d**). Colorless oil, 67% yield. ^1^H-NMR (500 MHz, DMSO-*d*_6_) δ 7.78 (d, *J* = 1.5 Hz, 1H, Pyrrole-5H), 7.65 (d, *J* = 1.5 Hz, 1H, Ar-3H), 7.60 (d, *J* = 8.5 Hz, 1H, Ar-6H), 7.43 (dd, *J* = 8.0, 1.5 Hz, 1H, Ar-5H), 7.28 (d, *J* = 1.5 Hz, 1H, Pyrrole-3H), 5.68 (s, 2H, NCH_2_O), 3.78 (s, 3H, CH_3_), 3.52 (t, *J* = 8.0 Hz, 2H, O*CH_2_*CH_2_Si), 0.82 (t, *J* = 8.0 Hz, 2H, OCH_2_*CH_2_*Si), −0.07 (s, 9H, Si(CH_3_)_3_). ^13^C-NMR (100 MHz, DMSO-*d_6_*) δ: 160.8, 132.0, 131.9, 131.8, 130.1, 130.0, 128.1, 122.0, 119.7, 119.0, 77.0, 65.6, 51.7, 17.6, −1.0. HRMS (ESI): *m/z* [M + H]^+^ calcd for C_18_H_24_Cl_2_NO_3_Si: 400.0897; found: 400.0892.

*Methyl 4-(4-fluorophenyl)-1-((2-(trimethylsilyl)ethoxy)methyl)-1H-pyrrole-2-carboxylate* (**3e**). Colorless oil, 70% yield. ^1^H-NMR (400 MHz, DMSO-*d*_6_) δ: 7.77 (d, *J* = 2.0 Hz, 1H, Pyrrole-5H), 7.66–7.63 (m, 2H, Ar-2,6H), 7.31 (d, *J* = 2.0 Hz, 1H, Pyrrole-3H), 7.17 (t, *J* = 8.8 Hz, 2H, Ar-3,5H), 5.63 (s, 2H, NCH_2_O), 3.77 (s, 3H, CH_3_), 3.50 (t, *J* = 8.0 Hz, 2H, O*CH_2_*CH_2_Si), 0.81 (t, *J* = 8.0 Hz, 2H, OCH_2_*CH_2_*Si), −0.08 (s, 9H, Si(CH_3_)_3_). ^13^C-NMR (100 MHz, DMSO-*d_6_*) δ: 161.2 (d, *J* = 241.1 Hz), 160.9, 130.8 (d, *J* = 2.9 Hz), 127.3, 127.1 (d, *J* = 7.8 Hz), 122.8 (d, *J* = 23.6 Hz), 116.1, 116.0, 115.9, 76.9, 65.6, 51.6, 17.6, −1.0. HRMS (ESI): *m/z* [M + H]^+^ calcd for C_18_H_25_FNO_3_Si: 350.1582; found: 350.1565.

*Methyl 4-(2-fluorophenyl)-1-((2-(trimethylsilyl)ethoxy)methyl)-1H-pyrrole-2-carboxylate* (**3f**). Colorless oil, 77% yield. ^1^H-NMR (500 MHz, DMSO-*d*_6_) δ: 7.76–7.72 (m, 2H, Pyrrole-5H, Ar-6H), 7.36 (s, 1H, Pyrrole-3H), 7.26–7.21 (m, 3H, Ar-3,4,5H), 5.68 (s, 2H, NCH_2_O), 3.78 (s, 3H, CH_3_), 3.51 (t, *J* = 7.5 Hz, 2H, O*CH_2_*CH_2_Si), 0.81 (t, *J* = 7.5 Hz, 2H, OCH_2_*CH_2_*Si), −0.07 (s, 9H, Si(CH_3_)_3_). ^13^C-NMR (100 MHz, DMSO-*d_6_*) δ: 160.9, 159.3 (d, *J* = 244.4 Hz), 129.3 (d, *J* = 8.6 Hz), 128.3 (d, *J* = 4.2 Hz), 128.2 (d, *J* = 8.4 Hz), 125.2 (d, *J* = 3.1 Hz), 122.4, 121.8 (d, *J* = 12.8 Hz), 117.5, 117.4, 116.5 (d, *J* = 22.1 Hz), 76.9, 65.6, 51.7, 17.5, −1.0. HRMS (ESI): *m/z* [M + H]^+^ calcd for C_18_H_25_FNO_3_Si: 350.1582; found: 350.1572.

*Methyl 4-(2,4-difluorophenyl)-1-((2-(trimethylsilyl)ethoxy)methyl)-1H-pyrrole-2-carboxylate* (**3g**). Colorless oil, 70% yield. ^1^H-NMR (500 MHz, DMSO-*d*_6_) δ: 7.78 (dd, *J* = 16.0, 9.0 Hz, 1H, Ar-3H), 7.74 (s, 1H, Pyrrole-5H), 7.34 (s, 1H, Pyrrole-3H), 7.28 (t, *J* = 9.5 Hz, 1H, Ar-6H), 7.10 (t, *J* = 8.5 Hz, 1H, Ar-5H), 5.68 (s, 2H, NCH_2_O), 3.79 (s, 3H, CH_3_), 3.51 (t, *J* = 8.0 Hz, 2H, O*CH_2_*CH_2_Si), 0.82 (t, *J* = 8.0 Hz, 2H, OCH_2_*CH_2_*Si), −0.07 (s, 9H, Si(CH_3_)_3_). ^13^C-NMR (100 MHz, DMSO-*d_6_*) δ: 161.1 (dd, *J* = 244.0, 12.2 Hz), 160.8, 159.3 (dd, *J* = 247.3, 11.9 Hz), 129.4 (dd, *J* = 9.3, 5.9 Hz), 129.0 (d, *J* = 8.5 Hz), 122.4, 118.5 (dd, *J* = 13.0, 3.6 Hz), 117.3 (d, *J* = 3.5 Hz), 116.8, 112.3 (dd, *J* = 21.0, 3.2 Hz), 104.8 (t, *J* = 26.1 Hz), 76.9, 65.6, 51.6, 17.5, −1.1. HRMS (ESI): *m/z* [M + H]^+^ calcd for C_18_H_24_F_2_NO_3_Si: 368.1488; found: 368.1480.

*Methyl 4-(4-nitrophenyl)-1-((2-(trimethylsilyl)ethoxy)methyl)-1H-pyrrole-2-carboxylate* (**3h**). Yellow solid, 62% yield. M.p. 82–83 °C. ^1^H-NMR (400 MHz, DMSO-*d*_6_) δ: 8.20 (d, *J* = 8.4 Hz, 2H, Ar-3,5H), 8.08 (d, *J* = 1.6 Hz, 1H, Pyrrole-5H), 7.91 (d, *J* = 8.4 Hz, 2H, Ar-2,6H), 7.51 (d, *J* = 2.0 Hz, 1H, Pyrrole-3H), 5.66 (s, 2H, NCH_2_O), 3.79 (s, 3H, CH_3_), 3.52 (t, *J* = 8.0 Hz, 2H, O*CH_2_*CH_2_Si), 0.83 (t, *J* = 8.0 Hz, 2H, OCH_2_*CH_2_*Si), −0.07 (s, 9H, Si(CH_3_)_3_). ^13^C-NMR (100 MHz, DMSO-*d*_6_) δ: 160.8, 145.7, 141.3, 129.4, 125.8, 124.7, 123.5, 121.8, 116.8, 77.2, 65.8, 51.8, 17.5, −1.0. HRMS (ESI): *m/z* [M + H]^+^ calcd for C_18_H_25_N_2_O_5_Si: 377.1527; found: 377.1545.

*Methyl 4-(4-(trifluoromethyl)phenyl)-1-((2-(trimethylsilyl)ethoxy)methyl)-1H-pyrrole-2-carboxylate* (**3i**). Colorless oil, 68% yield. ^1^H-NMR (500 MHz, DMSO-*d*_6_) δ: 7.95 (s, 1H, Pyrrole-5H), 7.83 (d, *J* = 5.0 Hz, 2H, Ar-3,5H), 7.67 (brs, 2H, Ar-2, 6H), 7.43 (s, 1H, Pyrrole-3H), 5.65 (s, 2H, NCH_2_O), 3.78 (s, 3H, CH_3_), 3.51 (t, *J* = 7.5 Hz, 2H, O*CH_2_*CH_2_Si), 0.82 (t, *J* = 7.5 Hz, 2H, OCH_2_*CH_2_*Si), −0.08 (s, 9H, Si(CH_3_)_3_). ^13^C-NMR (100 MHz, DMSO-*d*_6_) δ: 160.9, 138.4, 128.5, 126.9, 126.1 (q, *J* = 3.7 Hz), 125.6, 125.1 (q, *J* = 236.0 Hz), 123.2, 122.3, 116.5, 77.1, 65.7, 51.7, 17.5, −1.0. HRMS (ESI): *m/z* [M + H]^+^ calcd for C_19_H_25_F_3_NO_3_Si: 400.1550; found: 400.1567.

*Methyl 4-(4-methoxyphenyl)-1-((2-(trimethylsilyl)ethoxy)methyl)-1H-pyrrole-2-carboxylate* (**3j**). Colorless oil, 77% yield. ^1^H-NMR (400 MHz, DMSO-*d*_6_) δ: 7.68 (d, *J* = 1.6 Hz, 1H, Pyrrole-5H), 7.53 (d, *J* = 8.8 Hz, 2H, Ar-2,6H), 7.25 (d, *J* = 2.0 Hz, 1H, Pyrrole-3H), 6.92 (d, *J* = 8.8 Hz, 2H, Ar-3,5H), 5.63 (s, 2H, NCH_2_O), 3.76 (s, 3H, CH_3_), 3.75 (s, 3H, CH_3_), 3.50 (t, *J* = 8.0 Hz, 2H, O*CH_2_*CH_2_Si), 0.81 (t, *J* = 8.0 Hz, 2H, OCH_2_*CH_2_*Si), −0.07 (s, 9H, Si(CH_3_)_3_). ^13^C-NMR (100 MHz, DMSO-*d*_6_) δ: 161.0, 158.3, 126.8, 126.6, 126.4, 123.8, 122.5, 115.8, 114.6, 76.8, 65.5, 55.5, 51.5, 17.6, −1.0. HRMS (ESI): *m/z* [M + H]^+^ calcd for C_19_H_28_NO_4_Si: 362.1782; found: 362.1792.

*Methyl 4-(2-methoxyphenyl)-1-((2-(trimethylsilyl)ethoxy)methyl)-1H-pyrrole-2-carboxylate* (**3k**). Colorless oil, 87% yield. ^1^H-NMR (400 MHz, DMSO-*d*_6_) δ: 7.76 (d, *J* = 2.0 Hz, 1H, Pyrrole-5H), 7.59 (dd, *J* = 8.0, 2.0 Hz, 1H, Ar-6H), 7.37 (d, *J* = 2.0 Hz, 1H, Pyrrole-3H), 7.20 (td, *J* = 7.6, 1.6 Hz, 1H, Ar-4H), 7.05 (d, *J* = 7.6 Hz, 1H, Ar-3H), 6.95 (td, *J* = 7.6, 1.2 Hz, 1H, Ar-5H), 5.67 (s, 2H, NCH_2_O), 3.86 (s, 3H, CH_3_), 3.77 (s, 3H, CH_3_), 3.51 (t, *J* = 7.6 Hz, 2H, O*CH_2_*CH_2_Si), 0.82 (t, *J* = 7.6 Hz, 2H, OCH_2_*CH_2_*Si), −0.06 (s, 9H, Si(CH_3_)_3_). ^13^C-NMR (100 MHz, DMSO-*d*_6_) δ: 161.1, 156.3, 129.9, 127.7, 122.6, 121.6, 121.2, 120.0, 118.0, 112.1, 76.7, 65.4, 55.8, 51.5, 17.6, −1.0. HRMS (ESI): *m/z* [M + H]^+^ calcd for C_19_H_28_NO_4_Si: 362.1782; found: 362.1784.

*Methyl 4-(p-tolyl)-1-((2-(trimethylsilyl)ethoxy)methyl)-1H-pyrrole-2-carboxylate* (**3l**). Colorless oil, 73% yield. ^1^H-NMR (400 MHz, DMSO-*d*_6_) δ: 7.74 (d, *J* = 2.0 Hz, 1H, Pyrrole-5H), 7.49 (d, *J* = 8.0 Hz, 2H, Ar-2,6H), 7.28 (d, *J* = 2.0 Hz, 1H, Pyrrole-3H), 7.16 (d, *J* = 8.0 Hz, 2H, Ar-3,5H), 5.63 (s, 2H, NCH_2_O), 3.76 (s, 3H, CH_3_), 3.50 (t, *J* = 8.0 Hz, 2H, O*CH_2_*CH_2_Si), 2.29 (s, 3H, CH_3_), 0.81 (t, *J* = 8.0 Hz, 2H, OCH_2_*CH_2_*Si), −0.07 (s, 9H, Si(CH_3_)_3_). ^13^C-NMR (100 MHz, DMSO-*d*_6_) δ: 161.0, 135.7, 131.4, 129.8, 127.1, 125.1, 123.9, 122.6, 116.0, 76.8, 65.5, 51.6, 21.1, 17.6, −1.0. HRMS (ESI): *m/z* [M + H]^+^ calcd for C_19_H_28_NO_3_Si: 346.1833; found: 346.1839.

*Methyl 4-(o-tolyl)-1-((2-(trimethylsilyl)ethoxy)methyl)-1H-pyrrole-2-carboxylate* (**3m**). Colorless oil, 52% yield. ^1^H-NMR (400 MHz, DMSO-*d*_6_) δ: 7.52 (d, *J* = 2.0 Hz, 1H, Pyrrole-5H), 7.32 (dd, *J* = 7.6, 2.0 Hz, 1H, Ar-6H), 7.25–7.16 (m, 3H, Ar-3,4,5H), 7.09 (d, *J* = 2.0 Hz, 1H, Pyrrole-3H), 5.66 (s, 2H, NCH_2_O), 3.76 (s, 3H, CH_3_), 3.51 (t, *J* = 8.0 Hz, 2H, O*CH_2_*CH_2_Si), 2.37 (s, 3H, CH_3_), 0.81 (t, *J* = 8.0 Hz, 2H, OCH_2_*CH_2_*Si), −0.07 (s, 9H, Si(CH_3_)_3_). ^13^C-NMR (100 MHz, DMSO-*d*_6_) δ: 161.0, 135.1, 134.2, 131.1, 129.5, 129.2, 127.0, 126.5, 123.4, 121.8, 119.1, 76.9, 65.5, 51.6, 21.5, 17.6, −1.0. HRMS (ESI): *m/z* [M + H]^+^ calcd for C_19_H_28_NO_3_Si: 346.1833; found: 346.1843.

*Methyl 4-(naphthalen-2-yl)-1-((2-(trimethylsilyl)ethoxy)methyl)-1H-pyrrole-2-carboxylate* (**3n**). Colorless oil, 76% yield. ^1^H-NMR (400 MHz, DMSO-*d*_6_) δ: 8.16 (dd, *J* = 7.2, 2.4 Hz, 1H, Ar-5H), 7.98–7.95 (m, 1H, Ar-4H), 7.87 (d, *J* = 8.0 Hz, 1H, Ar-8H), 7.65 (d, *J* = 2.0 Hz, 1H, Pyrrole-5H), 7.54–7.46 (m, 4H, Ar-1,3,6,7H), 7.18 (d, *J* = 2.0 Hz, 1H, Pyrrole-3H), 5.73 (s, 2H, NCH_2_O), 3.79 (s, 3H, CH_3_), 3.57 (t, *J* = 7.6 Hz, 2H, O*CH_2_*CH_2_Si), 0.84 (t, *J* = 7.6 Hz, 2H, OCH_2_*CH_2_*Si), −0.05 (s, 9H, Si(CH_3_)_3_). ^13^C-NMR (100 MHz, DMSO-*d*_6_) δ: 161.0, 134.1, 132.8, 131.3, 129.9, 128.9, 127.5, 127.0, 126.7, 126.3, 126.2, 125.5, 122.4, 122.2, 119.7, 77.0, 65.6, 51.7, 17.6, −1.0. HRMS (ESI): *m/z* [M + H]^+^ calcd for C_22_H_28_NO_3_Si: 382.1833; found: 382.1839.

*Methyl 4-(quinolin-3-yl)-1-((2-(trimethylsilyl)ethoxy)methyl)-1H-pyrrole-2-carboxylate* (**3o**). Colorless solid, 66% yield. M.p. 52–54 °C. ^1^H-NMR (400 MHz, DMSO-*d*_6_ ) δ: 9.21 (d, *J* = 2.0 Hz, 1H, Ar-2H), 8.53 (d, *J* = 2.0 Hz, 1H, Ar-4H), 8.06 (d, *J* = 2.0 Hz, 1H, Pyrrole-5H), 7.94 (d, *J* = 8.4 Hz, 1H, Ar-8H), 7.89 (d, *J* = 7.2 Hz, 1H, Ar-5H), 7.65 (td, *J* = 7.2, 1.6 Hz, 1H, Ar-6H), 7.57–7.53 (m, 2H, Ar-7H, Pyrrole-3H), 5.65 (s, 2H, NCH_2_O), 3.77 (s, 3H, CH_3_), 3.51 (t, *J* = 8.0 Hz, 2H, O*CH_2_*CH_2_Si), 0.80 (t, *J* = 8.0 Hz, 2H, OCH_2_*CH_2_*Si), −0.1 (s, 9H, Si(CH_3_)_3_). ^13^C-NMR (100 MHz, DMSO-*d*_6_) δ: 160.9, 149.0, 146.7, 130.1, 129.2, 129.1, 128.4, 128.3, 128.2, 127.5, 127.4, 123.2, 120.8, 116.5, 77.1, 65.7, 51.8, 17.6, −1.0. HRMS (ESI): *m/z* [M + H]^+^ calcd for C_21_H_27_N_2_O_3_Si: 383.1786; found: 383.1783.

*Methyl 4-(pyridin-3-yl)-1-((2-(trimethylsilyl)ethoxy)methyl)-1H-pyrrole-2-carboxylate* (**3p**). Colorless oil, 41% yield. ^1^H-NMR (500 MHz, DMSO-*d*_6_) δ: 8.88 (s, 1H, Py-2H), 8.40 (d, *J* = 3.5 Hz, 1H, Py-6H), 8.01 (d, *J* = 7.5 Hz, 1H, Py-4H), 7.93 (s, 1H, Pyrrole-5H), 7.43 (s, 1H, Pyrrole-3H), 7.36 (dd, *J* = 7.5, 5.0 Hz, 1H, Py-5H), 5.65 (s, 2H, NCH_2_O), 3.78 (s, 3H, CH_3_), 3.51 (t, *J* = 8.0 Hz, 2H, O*CH_2_*CH_2_Si), 0.82 (t, *J* = 8.0 Hz, 2H, OCH_2_*CH_2_*Si), −0.07 (s, 9H, Si(CH_3_)_3_). ^13^C-NMR (100 MHz, DMSO-*d*_6_) δ: 160.9, 147.6, 146.5, 132.3, 130.0, 127.9, 124.2, 123.1, 120.6, 116.2, 77.0, 65.7, 51.7, 17.5, −1.0. HRMS (ESI): *m/z* [M + H]^+^ calcd for C_17_H_25_N_2_O_3_Si: 333.1629; found: 333.1622.

*Methyl 4-(furan-2-yl)-1-((2-(trimethylsilyl)ethoxy)methyl)-1H-pyrrole-2-carboxylate* (**3q**). Colorless oil, 45% yield. ^1^H-NMR (500 MHz, DMSO-*d*_6_) δ: 7.61 (s, 1H, Furan-5H), 7.58 (s, 1H, Pyrrole-5H), 7.16 (s, 1H, Furan-4H), 6.57 (d, *J* = 2.5 Hz, 1H, Furan-3H), 6.49 (s, 1H, Pyrrole-3H), 5.64 (s, 2H, NCH_2_O), 3.76 (s, 3H, CH_3_), 3.49 (t, *J* = 8.0 Hz, 2H, O*CH_2_*CH_2_Si), 0.81 (t, *J* = 8.0 Hz, 2H, OCH_2_*CH_2_*Si), −0.08 (s, 9H, Si(CH_3_)_3_). ^13^C-NMR (100 MHz, DMSO-*d*_6_) δ: 160.8, 149.6, 141.5, 126.1, 122.6, 115.6, 115.1, 111.9, 104.1, 76.9, 65.6, 51.7, 17.5, −1.0. HRMS (ESI): *m/z* [M + H]^+^ calcd for C_16_H_24_NO_4_Si: 322.1469; found: 322.1483.

*Methyl 5-phenyl-1-((2-(trimethylsilyl)ethoxy)methyl)-1H-pyrrole-2-carboxylate* (**3r**). Colorless oil, 95% yield. ^1^H-NMR (500 MHz, DMSO-*d*_6_) δ: 7.55 (d, *J* = 7.5 Hz, 2H, Ar-2,6H), 7.49–7.44 (m, 3H, Ar-3,4,5H), 7.03 (d, *J* = 3.5 Hz, 1H, Pyrrole-3H), 6.33 (d, *J* = 4.0 Hz, 1H, Pyrrole-4H), 5.62 (s, 2H, NCH_2_O), 3.77 (s, 3H, CH_3_), 3.36 (t, *J* = 8.0 Hz, 2H, O*CH_2_*CH_2_Si), 0.73 (t, *J* = 8.0 Hz, 2H, OCH_2_*CH_2_*Si), −0.10 (s, 9H, Si(CH_3_)_3_). ^13^C-NMR (100 MHz, DMSO-*d_6_*) δ: 161.2, 142.5, 131.8, 129.5, 129.2, 128.8, 123.3, 119.3, 110.3, 73.4, 65.3, 51.6, 17.9, −1.0. HRMS (ESI): *m/z* [M + H]^+^ calcd for C_18_H_26_NO_3_Si: 332.1677; found: 332.1661.

*Methyl 3-phenyl-1-((2-(trimethylsilyl)ethoxy)methyl)-1H-pyrrole-2-carboxylate* (**3s**). Colorless solid, 99% yield. M.p. 87–88 °C. ^1^H-NMR (500 MHz, DMSO-*d_6_*) δ: 7.35–7.30 (m, 6H, Ar-2,3,4,5,6H, Pyrrole-5H), 6.24 (d, *J* = 2.0 Hz, 1H, Pyrrole-4H), 5.60 (s, 2H, NCH_2_O), 3.58 (s, 3H, CH_3_), 3.46 (t, *J* = 7.5 Hz, 2H, O*CH_2_*CH_2_Si), 0.81 (t, *J* = 7.5 Hz, 2H, OCH_2_*CH_2_*Si), −0.06 (s, 9H, Si(CH_3_)_3_). ^13^C-NMR (100 MHz, DMSO-*d_6_*) δ: 161.8, 136.3, 134.2, 129.4, 129.2, 128.2, 127.1, 118.1, 110.6, 77.6, 65.5, 51.3, 17.6, −1.0. HRMS (ESI): *m/z* [M + H]^+^ calcd for C_18_H_26_NO_3_Si: 332.1677; found: 332.1663.

### 3.4. Procedure for the Preparation of Methyl 4-phenyl-1H-pyrrole-2-carboxylate (***4a***)

A mixture of **3a** (500 mg, 1.5 mmol) and TBAF (3 mL, 1 mol/L THF) was stirred at 80 °C for 2 h and cooled to rt. Then, the reaction mixture was diluted with EtOAc and H_2_O. The aqueous phase was extracted with EtOAc, and the combined organic layer was washed with brine, dried with Na_2_SO_4_, and concentrated. Purification by flash chromatography (EtOAc in PE = 14%) gave **4a** (251 mg, 83%) as a colorless solid. M.p. 198–200 °C. ^1^H-NMR (500 MHz, DMSO-*d_6_*) δ: 12.07 (s, 1H, NH), 7.62 (d, *J* = 7.0 Hz, 2H, Ar-2,6H), 7.51 (s, 1H, Pyrrole-5H), 7.32 (t, *J* = 7.0 Hz, 2H, Ar-3,5H), 7.19–7.16 (m, 2H, Ar-4H, Pyrrole-3H), 3.79 (s, 3H, CH_3_). ^1^H-NMR data was in agreement with the literature [28]. ^13^C-NMR (100 MHz, DMSO-*d_6_*) δ: 161.3, 135.0, 129.1, 126.2, 125.6, 125.2, 123.2, 121.7, 112.4, 51.6. HRMS (ESI): *m/z* [M + H]^+^ calcd for C_12_H_12_NO_2_: 202.0863; found: 202.0856.

### 3.5. Procedure for the Preparation of 4-Phenyl-1-((2-(trimethylsilyl)ethoxy)methyl)-1H-pyrrole-2-carboxylic acid (***4b***)

A mixture of **3a** (250 mg, 0.75 mmol) and NaOH (60 mg, 1.5 mmol) in ethanol (6 mL) and H_2_O (4 mL) was stirred at 100 °C for 6 h. Then, the reaction mixture was concentrated to remove ethanol, and the residue was adjusted to pH 7 with HCl (2M). The aqueous phase was extracted with EtOAc, and the combined organic layer was washed with brine, dried with Na_2_SO_4_, and concentrated to give **4b** (200 mg, 84%) as a colorless solid. M.p. 83–85 °C. ^1^H-NMR (400 MHz, DMSO-*d_6_*) δ: 12.44 (s, 1H, COOH), 7.72 (d, *J* = 1.6 Hz, 1H, Pyrrole-5H), 7.59 (d, *J* = 7.2 Hz, 2H, Ar-2,6H), 7.34 (t, *J* = 7.6 Hz, 2H, Ar-3,5H), 7.26 (d, *J* = 2.0 Hz, 1H, Pyrrole-3H), 7.18 (t, *J* = 7.6 Hz, 1H, Ar-4H), 5.66 (s, 2H, NCH_2_O), 3.51 (t, *J* = 8.0 Hz, 2H, O*CH_2_*CH_2_Si), 0.82 (t, *J* = 8.0 Hz, 2H, OCH_2_*CH_2_*Si), −0.07 (s, 9H, Si(CH_3_)_3_). ^13^C-NMR (100 MHz, DMSO-*d_6_*) δ: 162.2, 134.4, 129.2, 126.8, 126.5, 125.1, 123.9, 123.6, 115.9, 76.7, 65.5, 17.6, −0.9. HRMS (ESI): *m/z* [M + H]^+^ calcd for C_17_H_24_NO_3_Si: 318.1520; found: 318.1489.

### 3.6. Procedure for the Preparation of (4-Phenyl-1-((2-(trimethylsilyl)ethoxy)methyl)-1H-pyrrol-2-yl)methanol (***4c***)

First, LiAlH_4_ (35 mg, 0.94 mmol) was added to a solution of **3a** (155 mg, 0.47 mmol) in anhydrous THF (6 mL) at rt. After stirring overnight, the reaction mixture was quenched with 15% NaOH (0.11 mL) and extracted with EtOAc/H_2_O, and the organic layer was washed with brine, dried with Na_2_SO_4_, and concentrated. Purification by flash chromatography (EtOAc in PE = 14%) gave **4c** (127 mg, 89%) as a yellow oil. ^1^H-NMR (500 MHz, DMSO-*d_6_*) δ: 7.49 (d, *J* = 7.5 Hz, 2H, Ar-2,6H), 7.31–7.28 (m, 3H, Ar-3,5H, Pyrrole-5H), 7.11 (t, *J* = 7.5 Hz, 1H, Ar-4H), 6.41 (s, 1H, Pyrrole-3H), 5.29 (s, 2H, NCH_2_O), 4.98 (brs, 1H, OH), 4.47 (d, *J* = 4.0 Hz, 2H, *CH_2_*OH), 3.50 (t, *J* = 8.0 Hz, 2H, O*CH_2_*CH_2_Si), 0.84 (t, *J* = 8.0 Hz, 2H, OCH_2_*CH_2_*Si), −0.04 (s, 9H, Si(CH_3_)_3_). ^13^C-NMR (100 MHz, DMSO-*d_6_*) δ: 135.9, 134.7, 129.1, 125.5, 124.7, 122.6, 120.0, 107.1, 76.0, 65.2, 55.3, 17.6, −0.9. HRMS (ESI): *m/z* [M + H]^+^ calcd for C_17_H_26_NO_2_Si: 304.1727; found: 304.1708.

## 4. Conclusions

In summary, we have developed an efficient Suzuki–Miyaura coupling reaction of SEM-protected bromopyrroles and arylboronic acids under mild conditions leading to aryl-substituted pyrroles. This methodology is a practical and straightforward way to synthesize various pyrrole building blocks for delivering novel compounds with broad functional group compatibility. The protocol displays some advantages compared to the reported methods, such as commercially available materials, no debrominated by-products being formed, and the SEM-protecting group being more tolerant than the BOC-protecting group under the reaction conditions. Around the privileged SEM-protected aryl-substituted pyrroles, we demonstrate the versatility of the pyrrole ring framework in synthetic transformations for constructing new structures. This protocol will offer the opportunity to explore other metal-catalyzed cross-coupling reactions using SEM-protected pyrroles and generate new bioactive compounds containing polyfunctionalized pyrroles.

## Figures and Tables

**Table 1:**
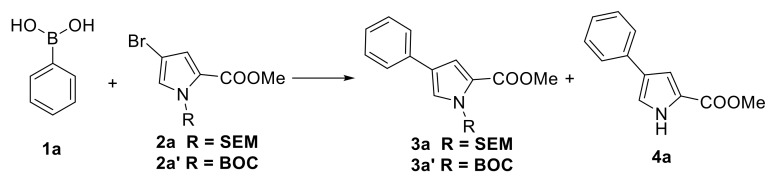
Screening reaction conditions of Suzuki coupling ^a^.

Entry	R	Catalyst	T (°C)	Base (equiv.)	Solvent	Time (h)	Yield (%) ^b^	
							3a (3a′)	4a
1	SEM	Pd(PPh_3_)_4_	90	Na_2_CO_3_ (2)	Dioxane/H_2_O (4:1)	1	61	0
2	SEM	Pd(PPh_3_)_2_Cl_2_	90	Na_2_CO_3_ (2)	Dioxane/H_2_O (4:1)	1	45	0
3	SEM	Pd(AcO)_2_	90	Na_2_CO_3_ (2)	Dioxane/H_2_O (4:1)	6	13	0
4	SEM	Pd(dppf)Cl_2_	90	Na_2_CO_3_ (2)	Dioxane/H_2_O (4:1)	6	28	0
5	SEM	Pd(PPh_3_)_4_	110	Na_2_CO_3_ (2)	DMF	6	trace	0
6 ^c^	SEM	Pd(PPh_3_)_4_	90	Na_2_CO_3_ (2)	Dioxane/H_2_O (4:1)	1	57	0
7 ^c^	SEM	Pd(PPh_3_)_4_	90	Na_2_CO_3_ (6)	Dioxane/H_2_O (4:1)	1	57	0
8	SEM	Pd(PPh_3_)_4_	90	Na_2_CO_3_ (2)	Dioxane/H_2_O (4:0.1)	6	5	0
9	SEM	Pd(PPh_3_)_4_	90	K_2_CO_3_ (2)	Dioxane/H_2_O (4:1)	5	45	0
10	SEM	Pd(PPh_3_)_4_	90	KF (2)	Dioxane/H_2_O (4:1)	6	32	0
11	SEM	Pd(PPh_3_)_4_	90	Cs_2_CO_3_ (2)	Dioxane/H_2_O (4:1)	5	85	0
12	SEM	Pd(PPh_3_)_4_	110	Cs_2_CO_3_ (2)	Dioxane/H_2_O (4:1)	3	75	0
13	SEM	Pd(PPh_3_)_4_	60	Cs_2_CO_3_ (2)	Dioxane/H_2_O (4:1)	5	53	0
14 ^d^	SEM	Pd(PPh_3_)_4_	90	Cs_2_CO_3_ (2)	Dioxane/H_2_O (4:1)	2	78	0
15	BOC	Pd(PPh_3_)_4_	90	Cs_2_CO_3_ (2)	Dioxane/H_2_O (4:1)	2	76	5
16	BOC	Pd(PPh_3_)_4_	90	Na_2_CO_3_ (2)	Dioxane/H_2_O (4:1)	2	64	11

^a^ General conditions: **1a** (1.5 mmol), **2a** (1 mmol), catalyst (10 mol %), and base in 10 mL of the indicated solvent under Ar_2_ atmosphere. ^b^ Isolated yield. ^c^ The equivalent ratio of **1a**:**2a** = 2:1. ^d^ 5 mol% Pd(PPh_3_)_4_.

## Supplementary Materials

The following are available online: ^1^H-NMR and HRMS of compounds **2a**–**c**, ^1^H-NMR, ^13^C-NMR and HRMS of compounds **3a**–**s** and **4a**–**c**.

## References

[1] Ma Z.N., Ma Z.C., Zhang D.W. (2018). Synthesis of multi-substituted pyrrole derivatives through [3+2] cycloaddition with tosylmethyl isocyanides (TosMICs) and electron-deficient compounds. Molecules.

[2] Hao F., Reddy A.R., Zhou C.Y., Che C.M. (2018). Cobalt(II) porphyrin catalyzed cascade reaction of pyrrolyl ketones for construction of polysubstituted pyrrolizidines and pyrrolizines. Adv. Synth. Catal..

[3] Guillon J., Le Borgne M., Rimbault C., Moreau S., Savrimoutou S., Pinaud N., Baratin S., Marchivie M., Roche S., Bollacke A. (2013). Synthesis and biological evaluation of novel substituted pyrrolo[1,2-a]quinoxaline derivatives as inhibitors of the human protein kinase CK2. Eur. J. Med. Chem..

[4] Desplat V., Moreau S., Gay A., Fabre S.B., Thiolat D., Massip S., Macky G., Godde F., Mossalayi D., Jarry C. (2010). Synthesis and evaluation of the antiproliferative activity of novel pyrrolo[1,2-a]quinoxaline derivatives, potential inhibitors of Akt kinase. Part II. J. Enzyme. Inhib. Med. Chem..

[5] Rahman K.M., Jackson P.J., James C.H., Basu B.P., Hartley J.A., de la Fuente M., Schatzlein A., Robson M., Pedley R.B., Pepper C. (2013). GC-targeted C8-linked pyrrolobenzodiazepine-biaryl conjugates with femtomolar in vitro cytotoxicity and in vivo antitumor activity in mouse models. J. Med. Chem..

[6] Burnham B.S., Berkowitz J.D., Orr Z., McCranor B., Cooper M., McCann K., Pillitteri K., Hallback J., Bergh L.-M., Gofman L. (2011). Lipid-lowering effects of polymers derived from halophenyl pyrroles. Lett. Drug Des. Discov..

[7] Lopez-Perez A., Robles-Machin R., Adrio J., Carretero J.C. (2007). Oligopyrrole Synthesis by 1,3-dipolar cycloaddition of azomethine ylides with bissulfonyl ethylenes. Angew. Chem. Int. Ed..

[8] Gao M., He C., Chen H., Bai R., Cheng B., Lei A. (2013). Synthesis of pyrroles by click reaction: Silver-catalyzed cycloaddition of terminal alkynes with isocyanides. Angew. Chem. Int. Ed..

[9] Farney E.P., Yoon T.P. (2014). Visible-light sensitization of vinyl azides by transition-metal photocatalysis. Angew. Chem. Int. Ed..

[10] O’Brien A.G., Levesque F., Seeberger P.H. (2011). Continuous flow thermolysis of azidoacrylates for the synthesis of heterocycles and pharmaceutical intermediates. Chem. Commun..

[11] Tiwari D.K., Pogula J., Sridhar B., Tiwari D.K., Likhar P.R. (2015). Nano-copper catalysed highly regioselective synthesis of 2,4-disubstituted pyrroles from terminal alkynes and isocyanides. Chem. Commun..

[12] Kudryavtsev K.V., Ivantcova P.M., Churakov A.V., Vasin V.A. (2012). Phenyl α-bromovinyl sulfone in cycloadditions with azomethine ylides: An unexpected facile aromatization of the cycloadducts into pyrroles. Tetrahedron Lett..

[13] Pasko C.M., Dissanayake A.A., Billow B.S., Odom A.L. (2016). One-pot synthesis of pyrroles using a titanium-catalyzed multicomponent coupling procedure. Tetrahedron.

[14] Chemler S.R., Fuller P.H. (2007). Heterocycle synthesis by copper facilitated addition of heteroatoms to alkenes, alkynes and arenes. Chem. Soc. Rev..

[15] Dowlut M., Mallik D., Organ M.G. (2010). An efficient low-temperature Stille-Migita cross-coupling reaction for heteroaromatic compounds by Pd-PEPPSI-IPent. Chem. Eur. J..

[16] Castro M.C.R., Raposo M.M.M. (2016). Synthesis of π-conjugated systems bearing thiophene and pyrrole heterocycles through palladium catalyzed cross-coupling reactions. Tetrahedron.

[17] Rodríguez N., Goossen L.J. (2011). Decarboxylative coupling reactions: A modern strategy for C-C-bond formation. Chem. Soc. Rev..

[18] Yuan K., Souleé J.-F., Doucet H. (2015). Functionalization of C−H Bonds via Metal-Catalyzed Desulfitative Coupling: An Alternative Tool for Access to Aryl- or Alkyl-Substituted (Hetero)arenes. ACS Catal..

[19] Handy S.T., Bregman H., Lewis J., Zhang X., Zhang Y. (2003). An unusual dehalogenation in the Suzuki coupling of 4-bromopyrrole-2-carboxylates. Tetrahedron Lett..

[20] Smith J.A., Ng S., White J. (2006). The regioselective synthesis of aryl pyrroles. Org. Biomol. Chem..

[21] Handy S.T., Zhang Y., Bregman H. (2004). A Modular synthesis of the lamellarins: Total synthesis of lamellarin G trimethyl ether. J. Org. Chem..

[22] Muchowski J.M., Solas D.R. (1984). Protecting groups for the pyrrole and indole nitrogen atom. the [2-(trimet hy1silyl)ethoxy]methyl moiety. lithiation of 1-[[2-(trimethylsilyl)ethoxy]methyl]pyrrole. J. Org. Chem..

[23] Whitten J.P., Matthews D.P., McCarthy J.R. (1986). [2-(Trimethylsilyl)ethoxy]methyl (SEM) as a novel and effective imidazole and fused aromatic imidazole protecting group. J. Org. Chem..

[24] Katz J.D., Overman L.E. (2004). Studies towards the total synthesis of palau’amine. Formation of 4,5-dihydropyrrole-2-carboxylate intermediates by alkene–enamide ring-closing metathesis. Tetrahedron.

[25] Toure B.B., Lane B.S., Sames D. (2006). Catalytic C-H arylation of SEM-protected azoles with palladium complexes of NHCs and phosphines. Org. Lett..

[26] Yamaguchi J., Seiple I.B., Young I.S., O’Malley D.P., Maue M., Baran P.S. (2008). Synthesis of 1,9-dideoxy-pre-axinellamine. Angew. Chem. Int. Ed..

[27] Sadler S.A., Hones A.C., Roberts B., Blakemore D., Marder T.B., Steel P.G. (2015). Multidirectional synthesis of substituted indazoles via iridium-catalyzed C-H borylation. J. Org. Chem..

[28] Mccomas C.C., Serrano-wu M.H., Vacca J.P. (2017). Fused quadracyclic compounds, compositions and uses thereof. U.S. Patent.

