# Supplementary material for: An Efficient Synthesis of Aryl-Substituted Pyrroles by the Suzuki–Miyaura Coupling Reaction of SEM-Protected Pyrroles
Source: Molecules. 2019 Apr 22;24(8):1594. doi: 10.3390/molecules24081594 (PMC6514742; doi:10.3390/molecules24081594)

Supplementary Materials

An Efficient Synthesis of Aryl-Substituted Pyrroles by the Suzuki–Miyaura Coupling Reaction of SEM-Protected Pyrroles

Keli Cui [1], Meng Gao [2], Hongyi Zhao [2], Dongfeng Zhang [2,*], Hong Yan [1,*], Haihong Huang [2,*]

1 College of Life Science and Bio-engineering, Beijing University of Technology, 100 Ping Le Yuan, Chaoyang District, Beijing 100124, China; ckl0615@163.com (K.C.); hongyan@bjut.edu.cn (H.Y.)

2 State Key Laboratory of Bioactive Substances and Function of Natural Medicine, Beijing Key Laboratory of Active Substance Discovery and Druggability Evaluation, Institute of Materia Medica, Peking Union Medical College and Chinese Academy of Medical Sciences, 1 Xian Nong Tan Street, Beijing 100050, China; gaomengss@imm.ac.cn (M.G.); zhaohongyicool@imm.ac.cn (H.Z.); zdf@imm.ac.cn (D.Z.); joyce@imm.ac.cn (H.H.)

* Correspondence: zdf@imm.ac.cn (D.Z.); hongyan@bjut.edu.cn (H.Y.); joyce@imm.ac.cn (H.H.)

Table of Contents

Copies of ^{1}H NMR and HRMS spectra of 2a-c……………………….S2-S4

Copies of ^{1}H, ^{13}C NMR and HRMS spectra of 3a-s……….………….S5-S22

Copies of ^{1}H, ^{13}C NMR and HRMS spectra of 4a-c…….......………..S43-S48

The synthesis method and yield of compound 4a in references........S44

Methyl 4-bromo-1-((2-(trimethylsilyl)ethoxy)methyl)-1H-pyrrole-2-carboxylate (**2a**)

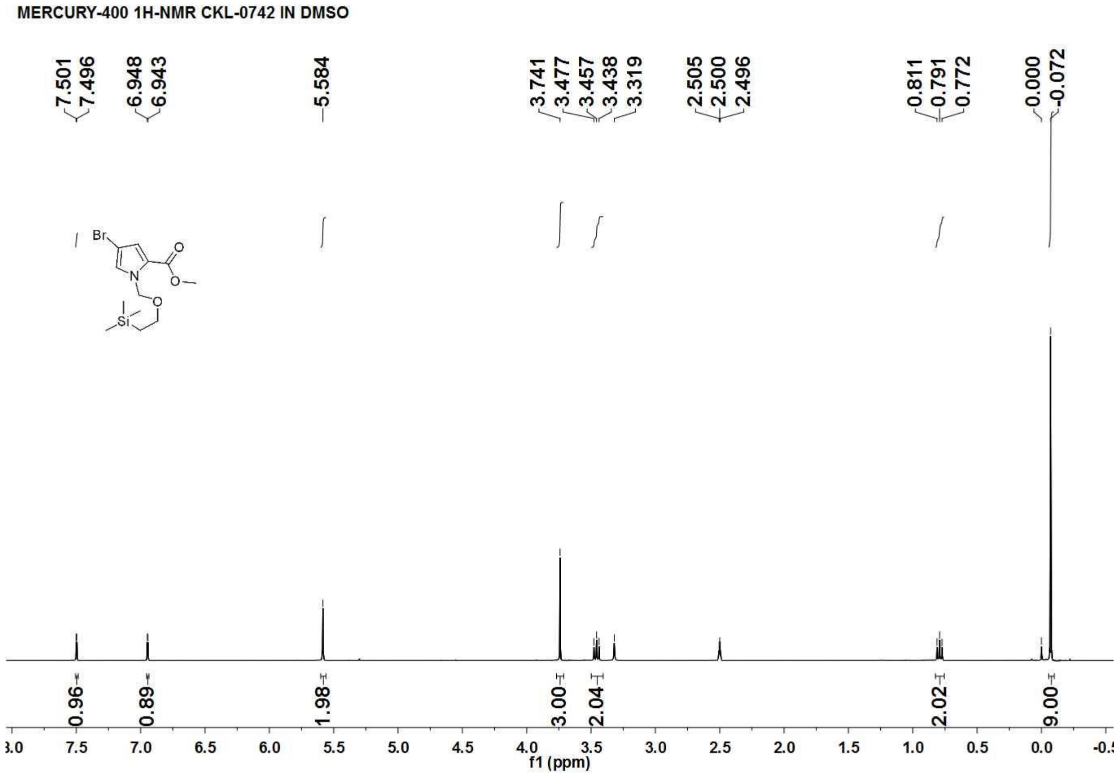

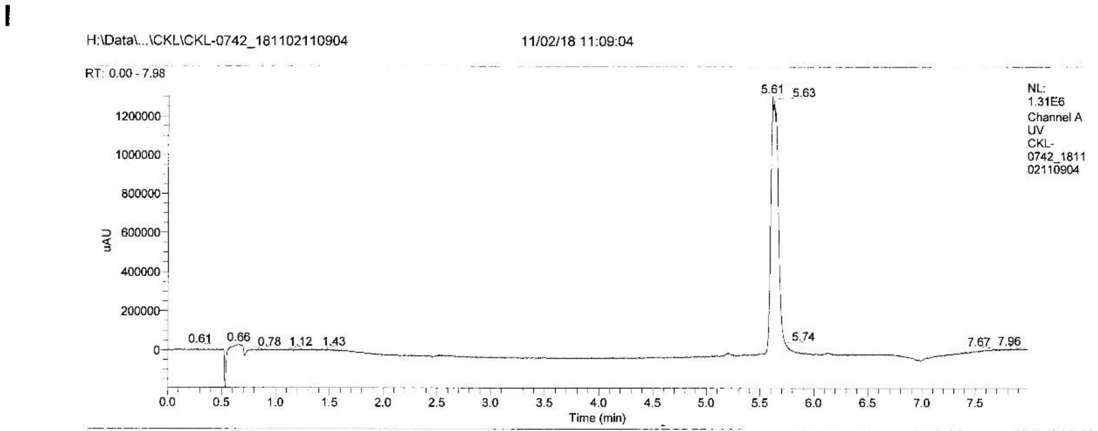

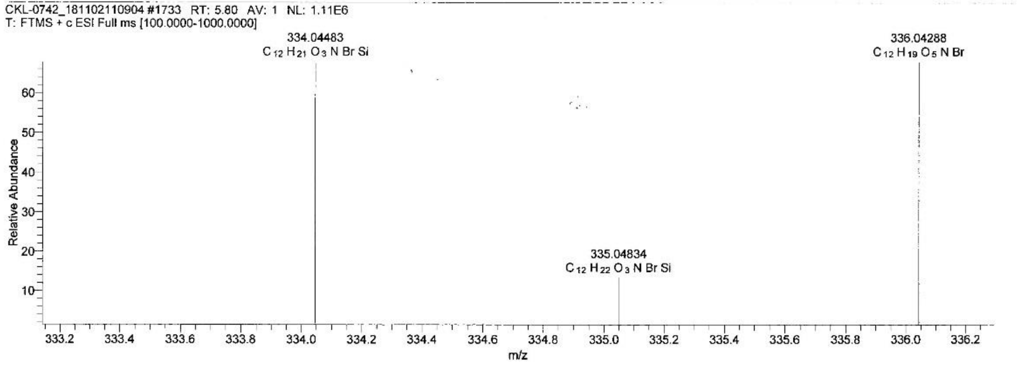

Methyl 5-bromo-1-((2-(trimethylsilyl)ethoxy)methyl)-1H-pyrrole-2-carboxylate (**2b**)

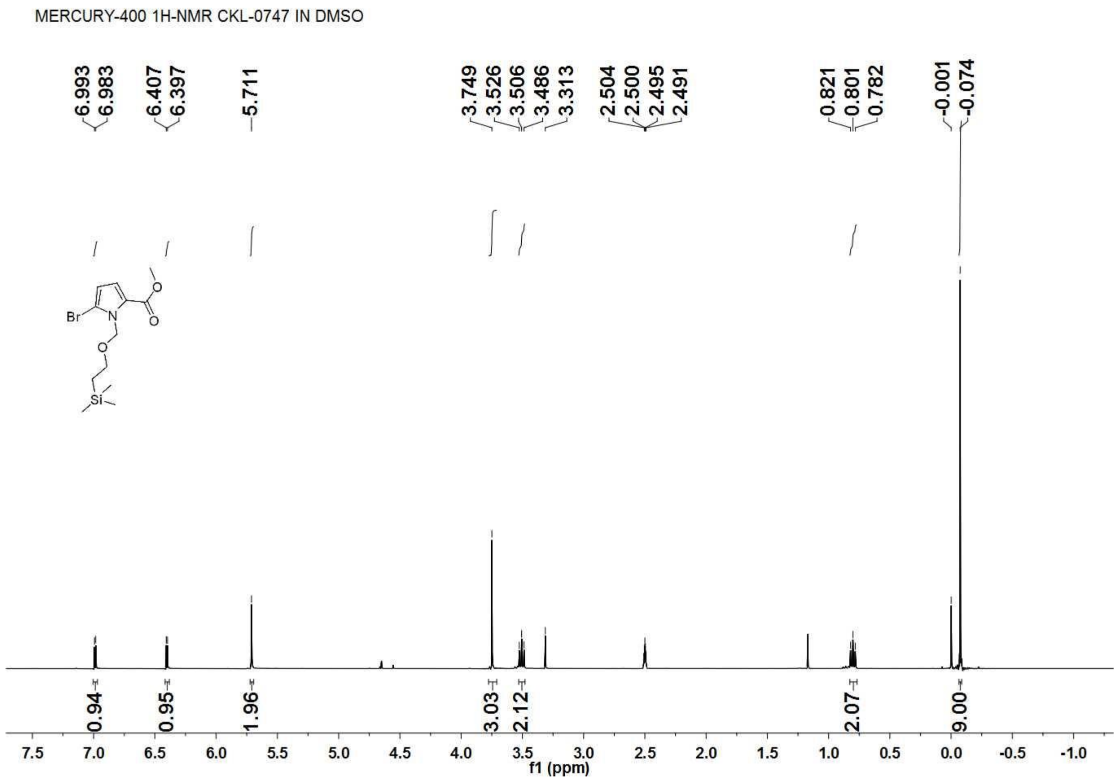

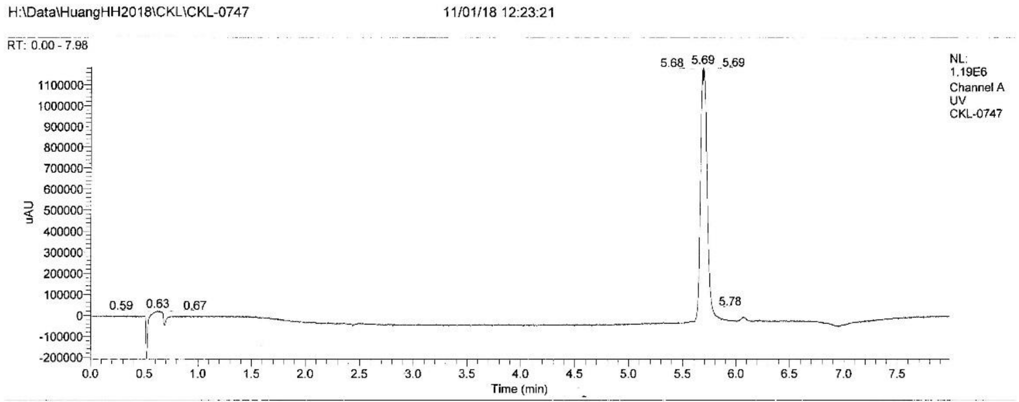

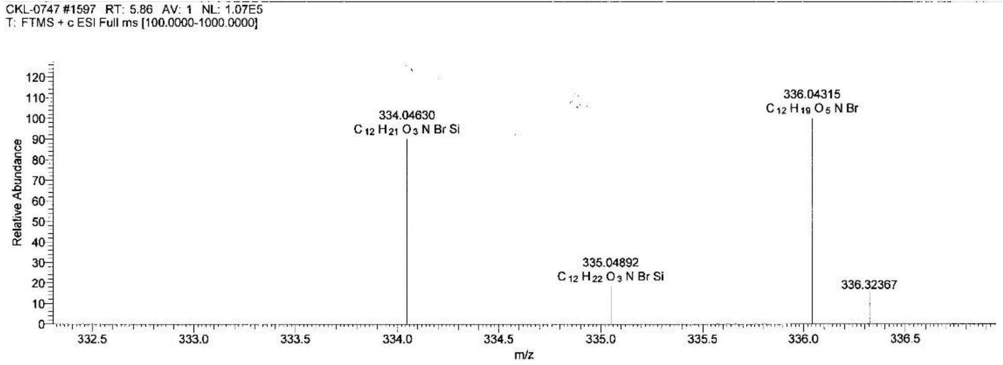

Methyl 3-bromo-1-((2-(trimethylsilyl)ethoxy)methyl)-1H-pyrrole-2-carboxylate (**2c**)

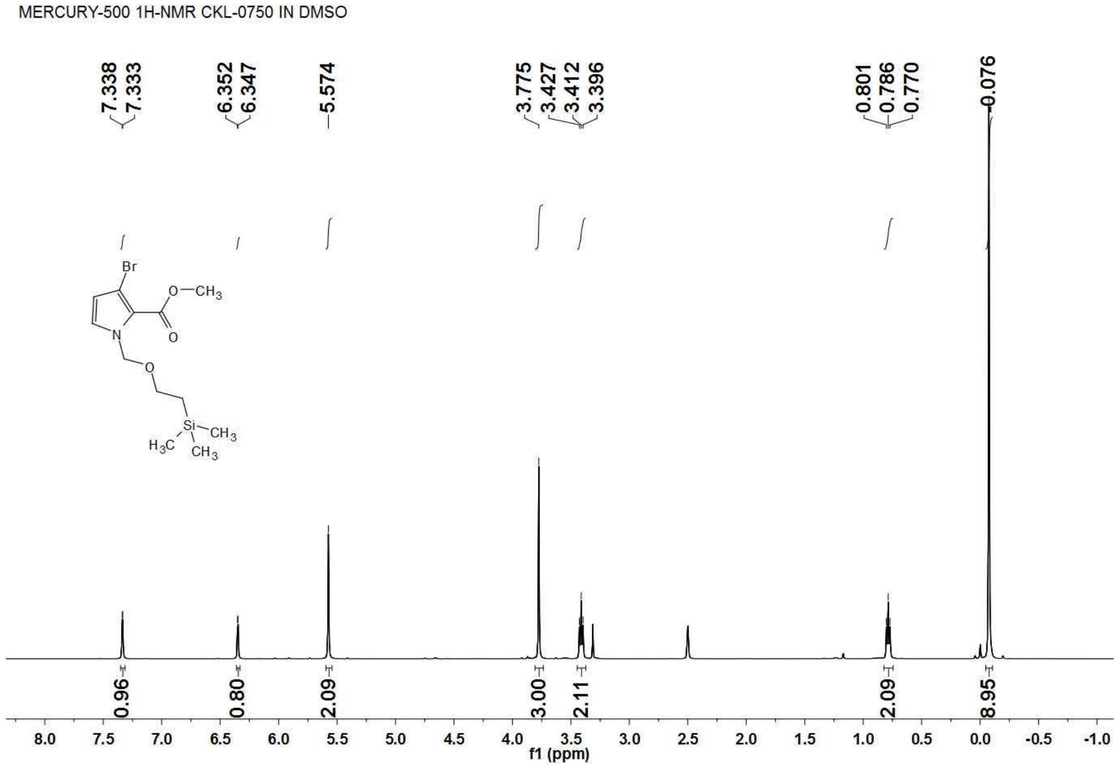

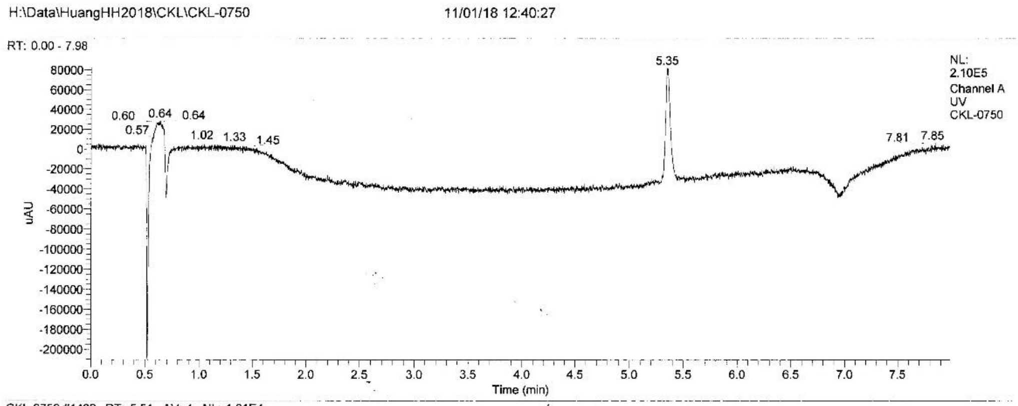

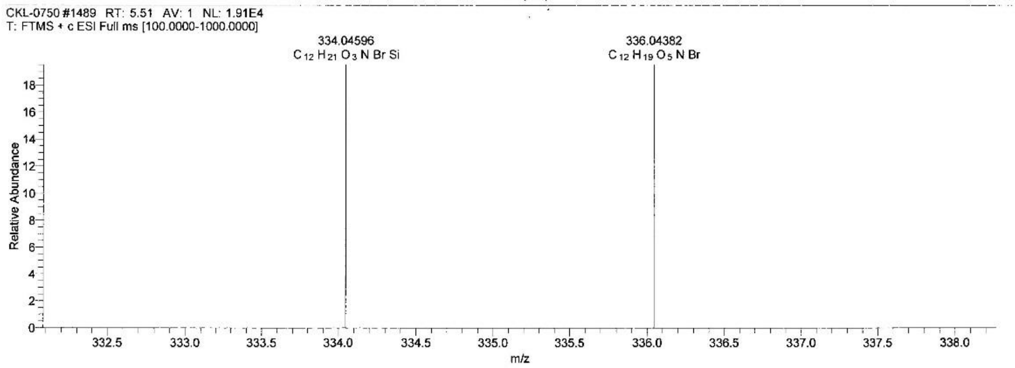

Methyl 4-phenyl-1-((2-(trimethylsilyl)ethoxy)methyl)-1H-pyrrole-2-carboxylate (**3a**)

MERCURY-400 1H-NMR CKL-0703 IN DMSO

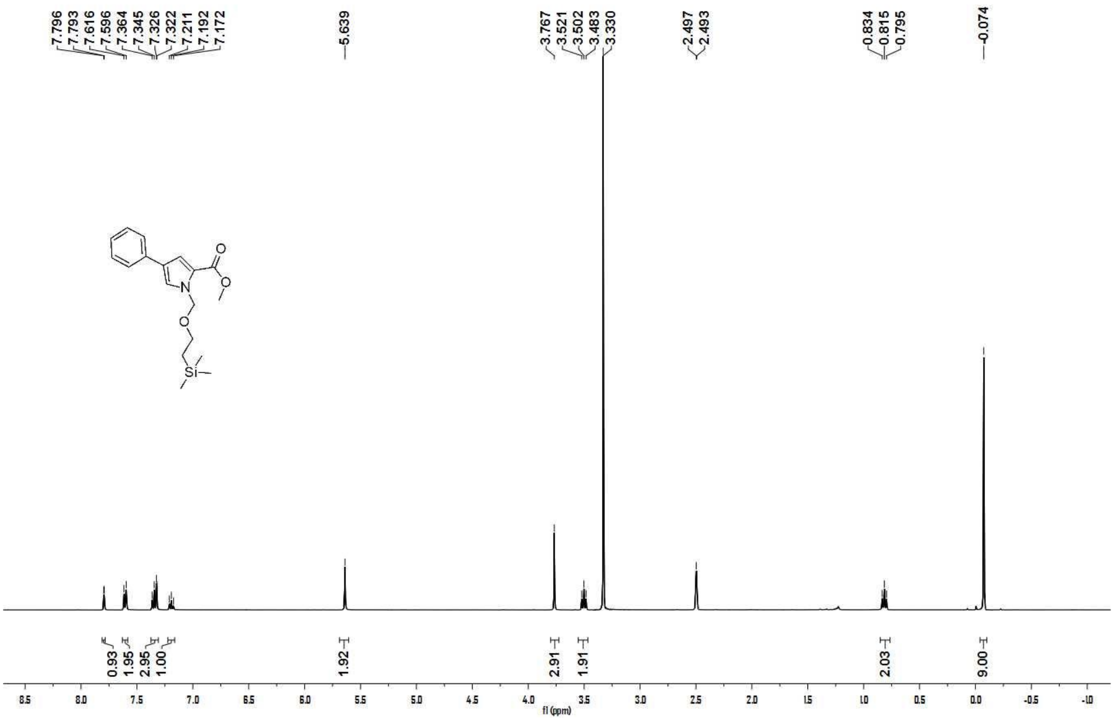

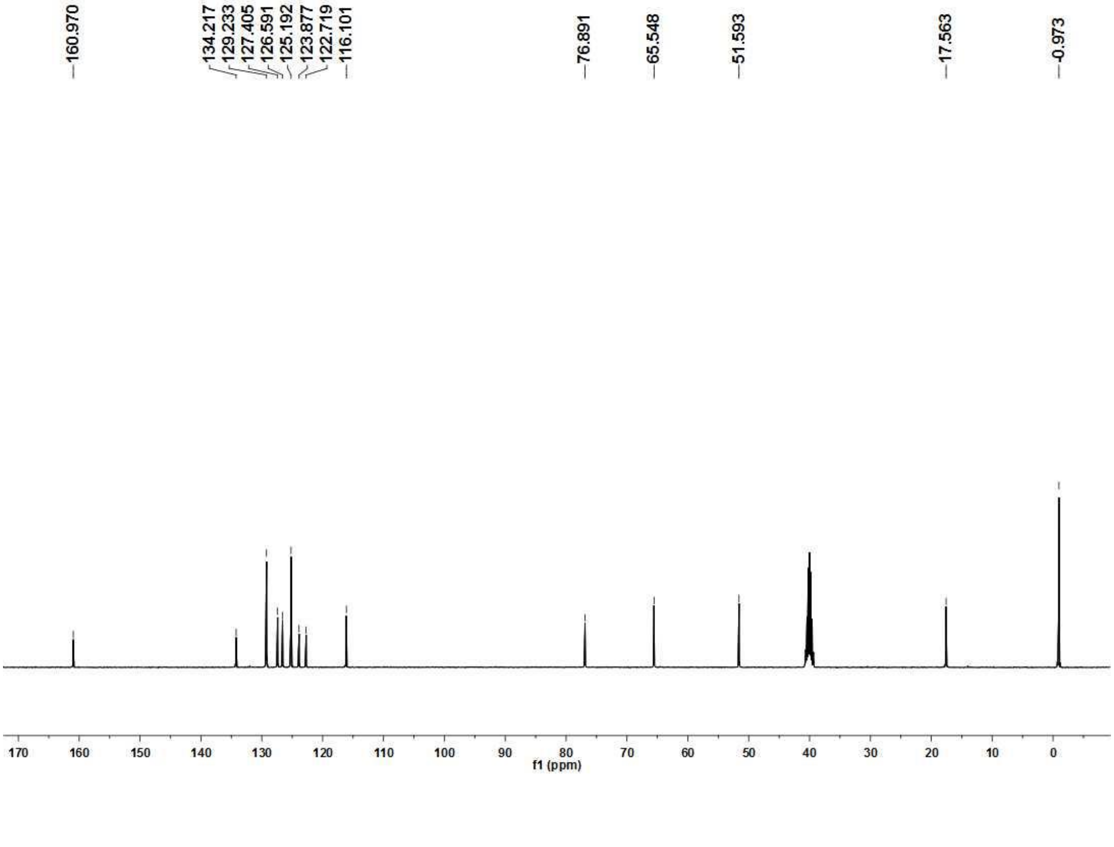

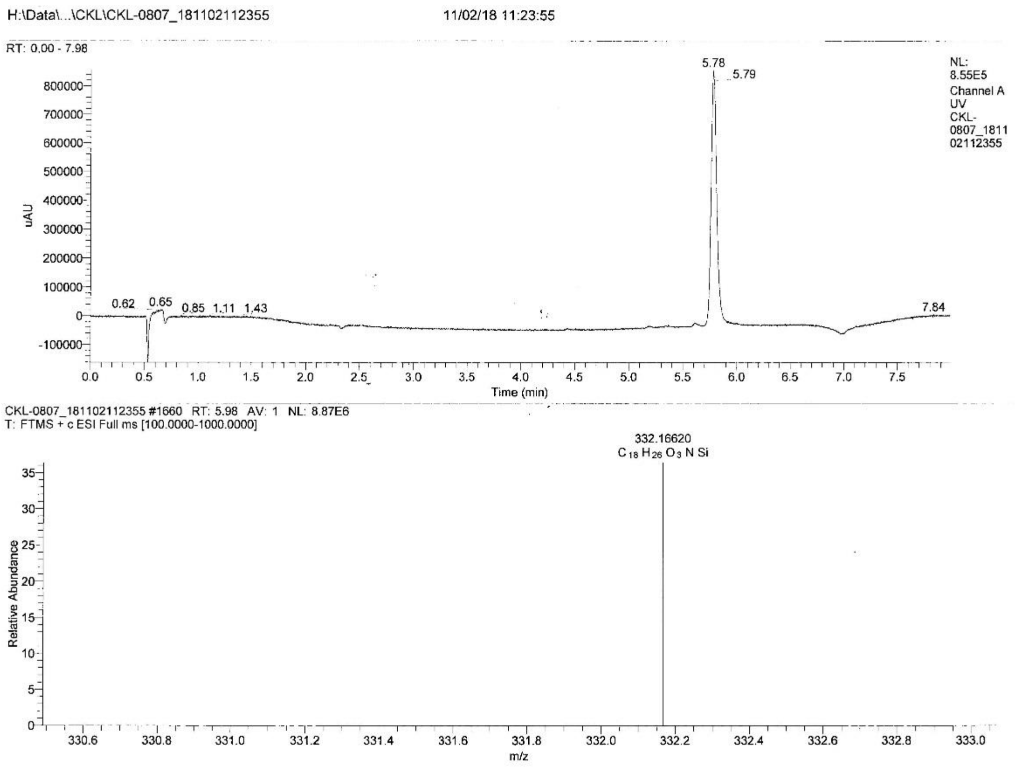

H:\Data\...\CKL\CKL-0807_181102112355
11/02/18 11:23:55
RT: 0.00 - 7.98
5.78
5.79
NL:
8.55E5
Channel A
UV
CKL-
0807_1811
02112355
uAU
800000
700000
600000
500000
400000
300000
200000
100000
0
-100000
0.62
0.65
0.85
1.11
1.43
7.84
0.0 0.5 1.0 1.5 2.0 2.5 3.0 3.5 4.0 4.5 5.0 5.5 6.0 6.5 7.0 7.5
Time (min)
CKL-0807_181102112355 #1660 RT: 5.98 AV: 1 NL: 8.87E6
T: FTMS + c ESI Full ms [100.0000-1000.0000]
332.16620
C 18 H 26 O 3 N Si
Relative Abundance
35
30
25
20
15
10
5
0
330.6 330.8 331.0 331.2 331.4 331.6 331.8 332.0 332.2 332.4 332.6 332.8 333.0
m/z

Methyl 4-(4-chlorophenyl)-1-((2-(trimethylsilyl)ethoxy)methyl)-1H-pyrrole-2-carboxy late (**3b**)

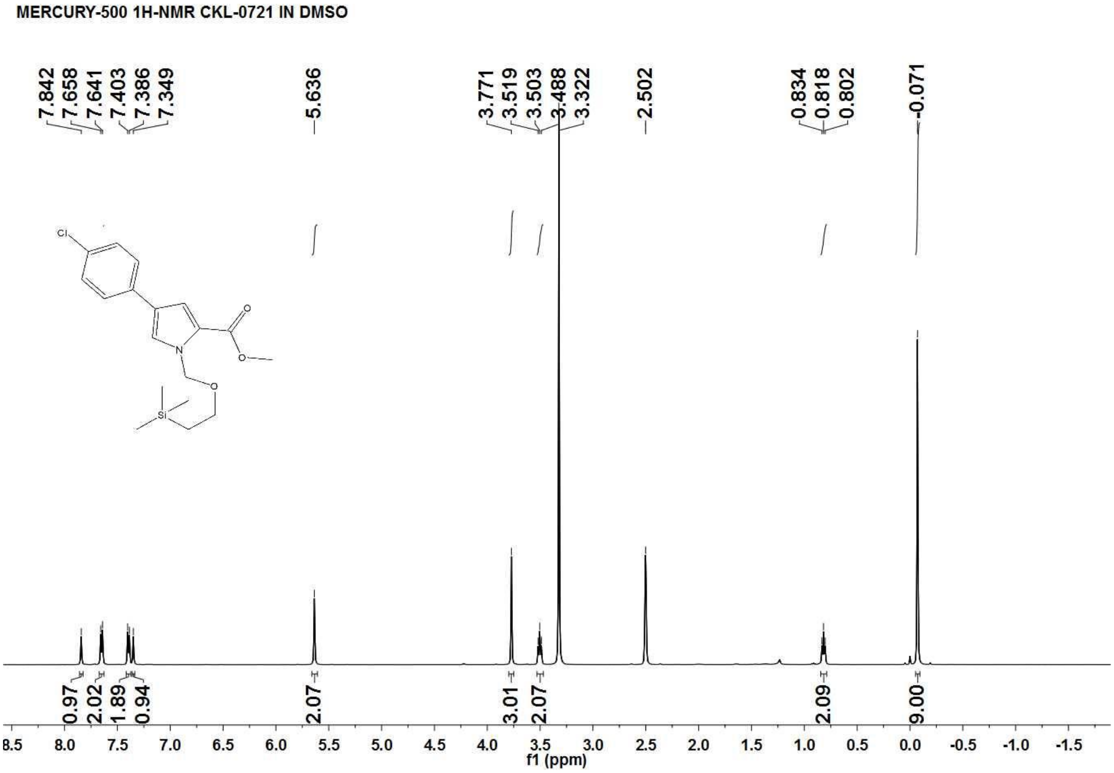

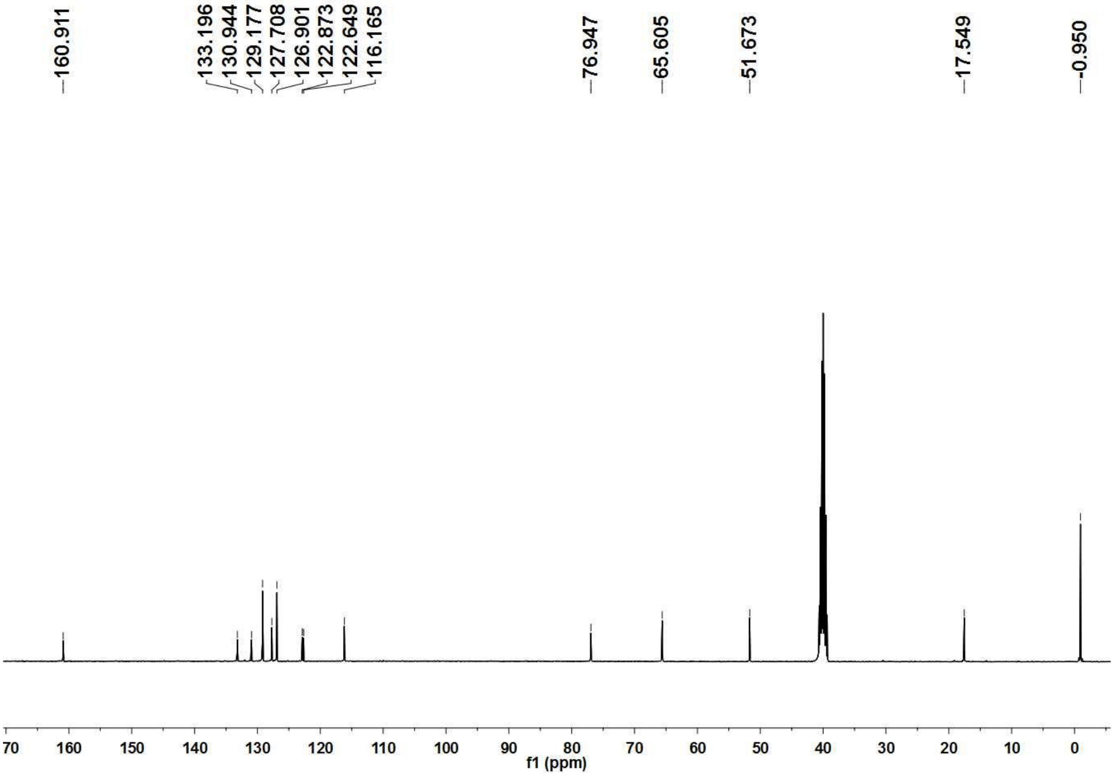

I

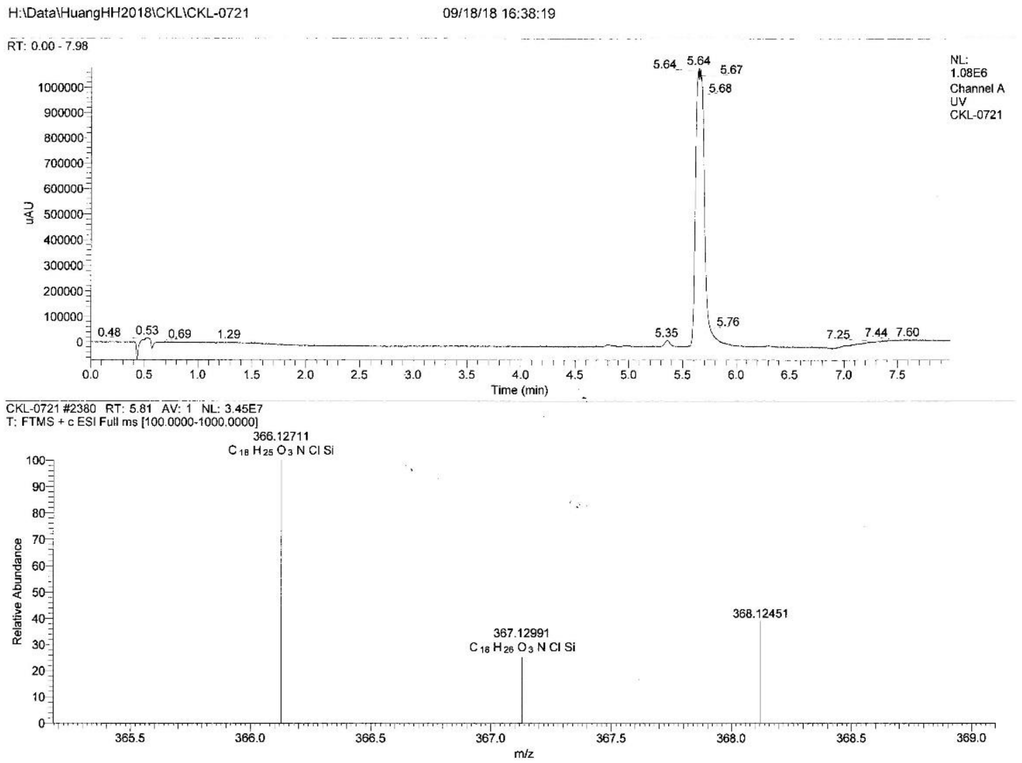

H:\Data\HuangHH2018\CKL\CKL-0721
09/18/18 16:38:19
RT: 0.00 - 7.98
NL: 1.08E6
Channel A
UV
CKL-0721
uAU
Time (min)
CKL-0721 #2380 RT: 5.81 AV: 1 NL: 3.45E7
T: FTMS + c ESI Full ms [100.0000-1000.0000]
366.12711
C 18 H 25 O 3 N Cl Si
367.12991
C 18 H 26 O 3 N Cl Si
368.12451
Relative Abundance
m/z

Methyl 4-(2-chlorophenyl)-1-((2-(trimethylsilyl)ethoxy)methyl)-1H-pyrrole-2-carboxylate (**3c**)

MERCURY-500 1H-NMR CKL-0722 IN DMSO

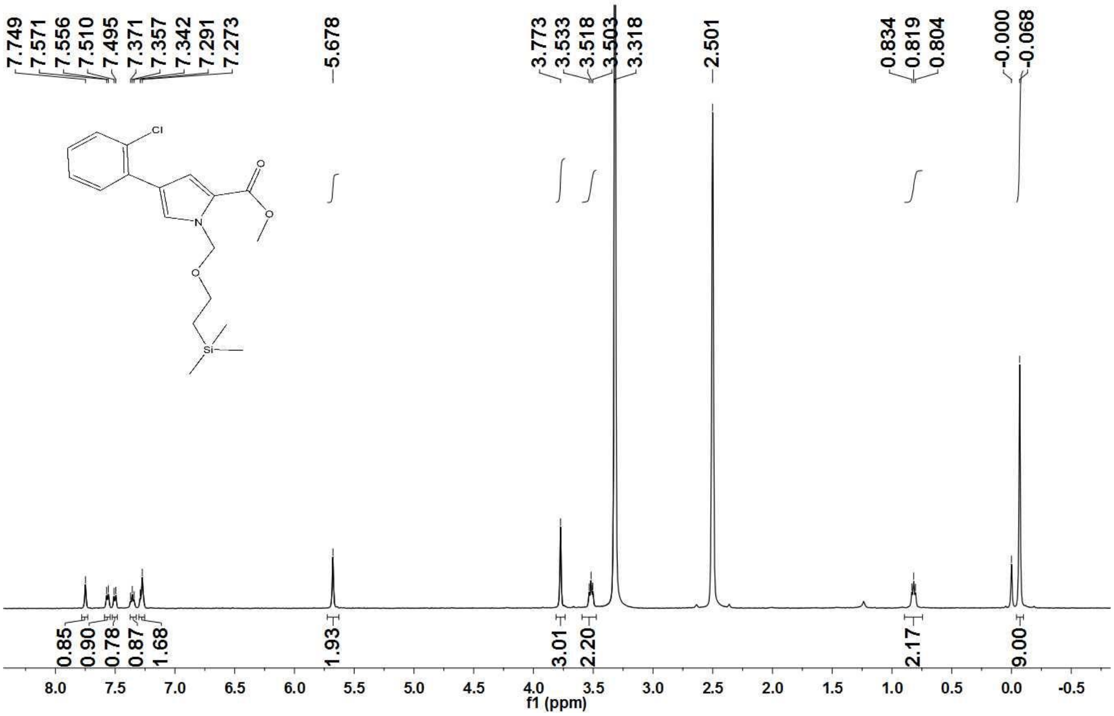

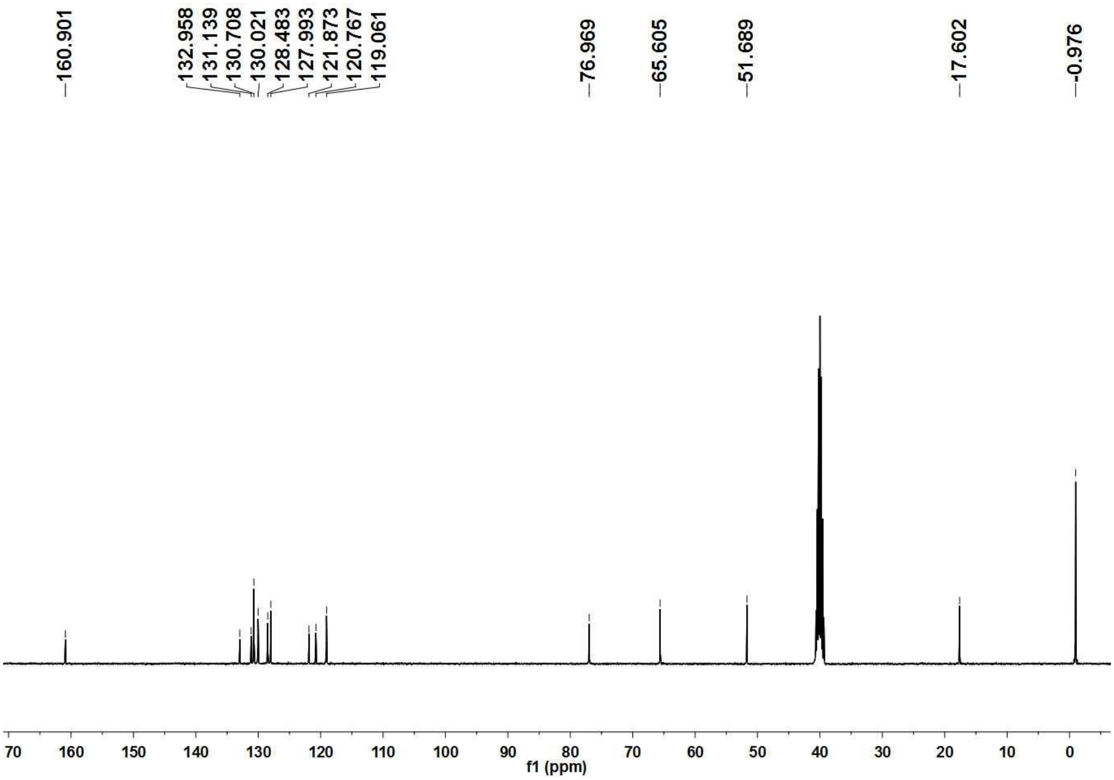

I

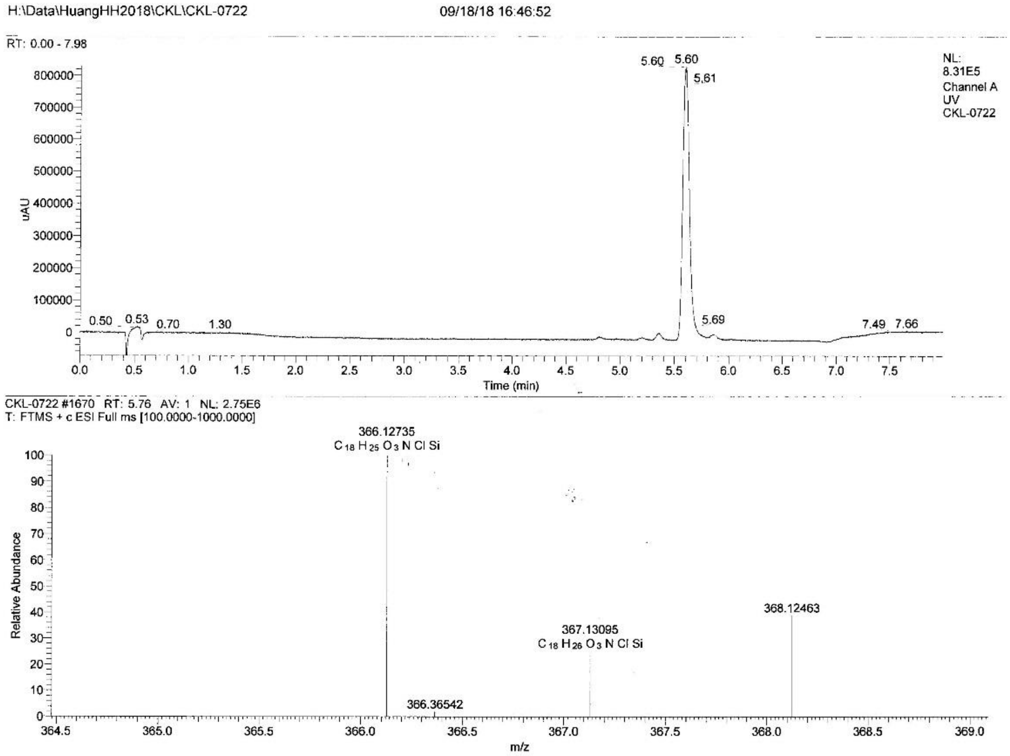
H:\Data\HuangHH2018\CKL\CKL-0722
09/18/18 16:46:52
RT: 0.00 - 7.98
NL:
8.31E5
Channel A
UV
CKL-0722
5.60
5.60
5.61
5.69
0.50
0.53
0.70
1.30
7.49
7.66
uAU
Time (min)
CKL-0722 #1670 RT: 5.76 AV: 1 NL: 2.75E6
T: FTMS + c ESI Full ms [100.0000-1000.0000]
366.12735
C 18 H 25 O 3 N Cl Si
367.13095
C 18 H 26 O 3 N Cl Si
368.12463
366.36542
Relative Abundance
m/z

Methyl 4-(2,4-dichlorophenyl)-1-((2-(trimethylsilyl)ethoxy)methyl)-1H-pyrrole-2-carboxylate (**3d**)

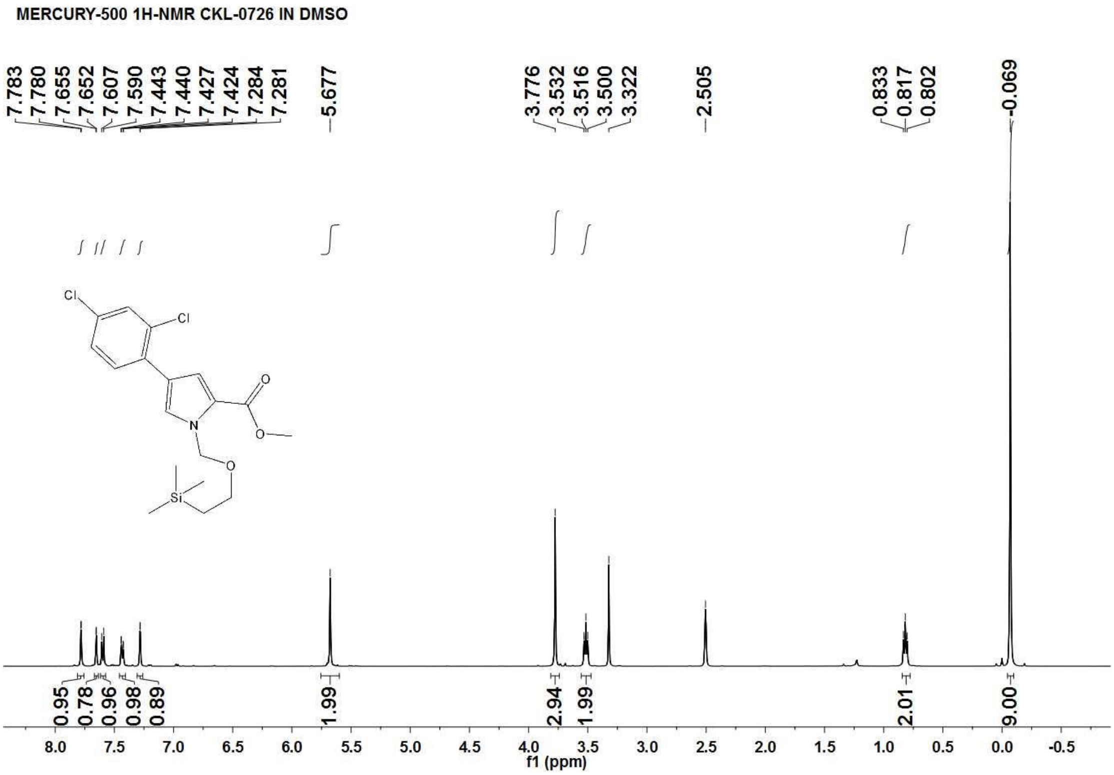

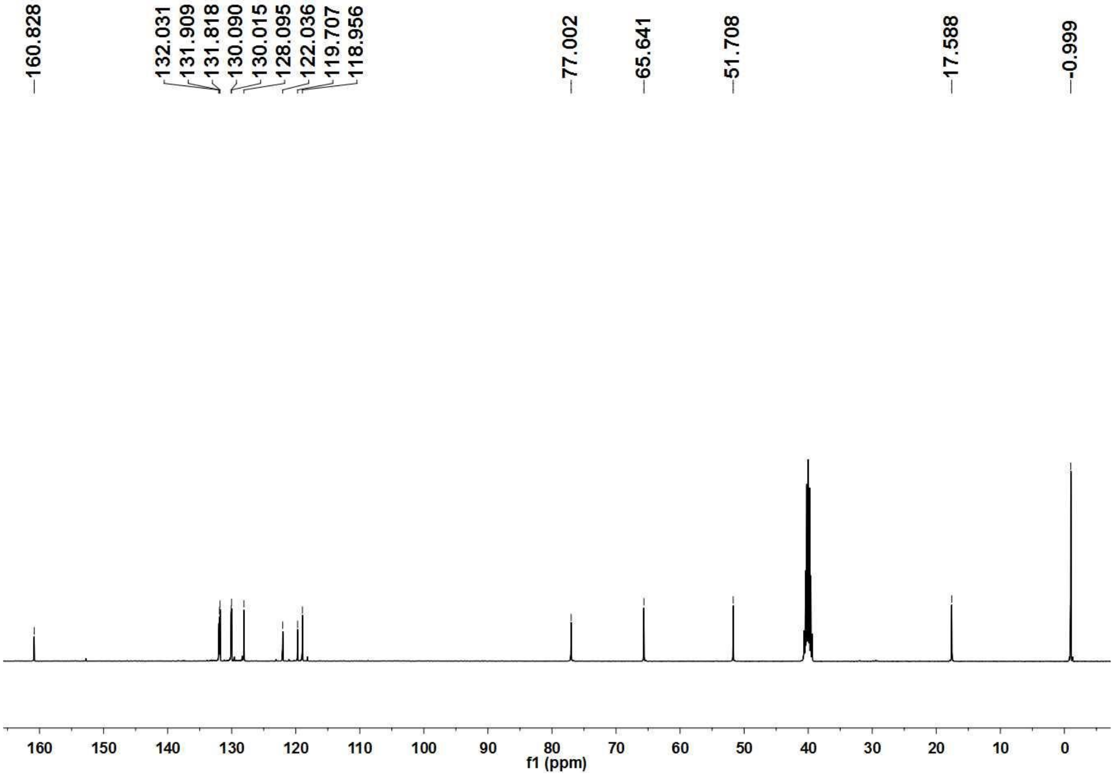

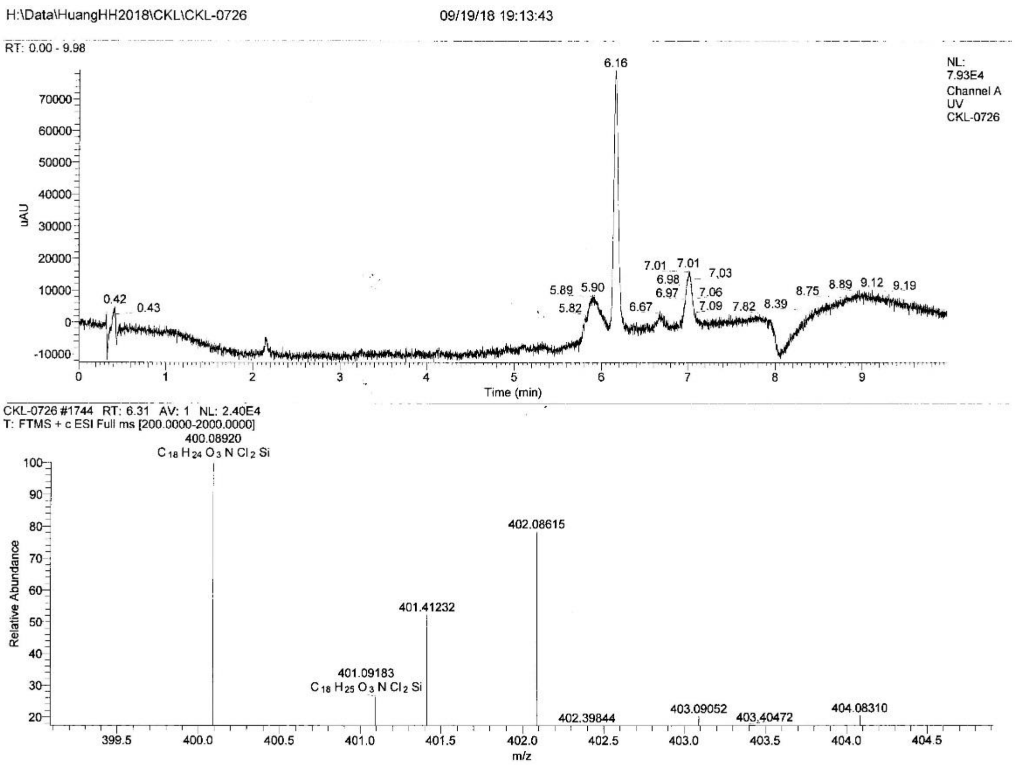
H:\Data\HuangHH2018\CKL\CKL-0726
09/19/18 19:13:43
RT: 0.00 - 9.98
NL: 7.93E4 Channel A UV CKL-0726
uAU
Time (min)
6.16
CKL-0726 #1744 RT: 6.31 AV: 1 NL: 2.40E4
T: FTMS + c ESI Full ms [200.0000-2000.0000]
400.08920
C18 H24 O3 N Cl2 Si
402.08615
401.41232
401.09183
C18 H25 O3 N Cl2 Si
402.39844
403.09052
403.40472
404.08310
Relative Abundance
m/z

Methyl 4-(4-fluorophenyl)-1-((2-(trimethylsilyl)ethoxy)methyl)-1H-pyrrole-2-carboxylate (**3e**)

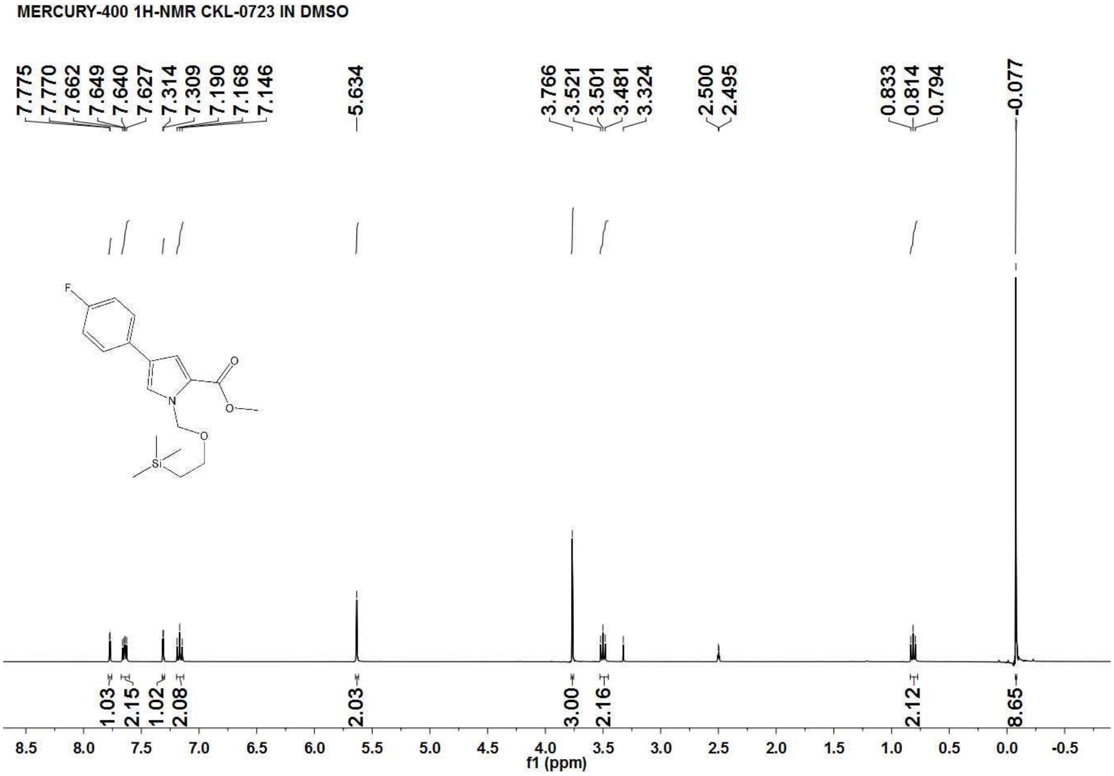

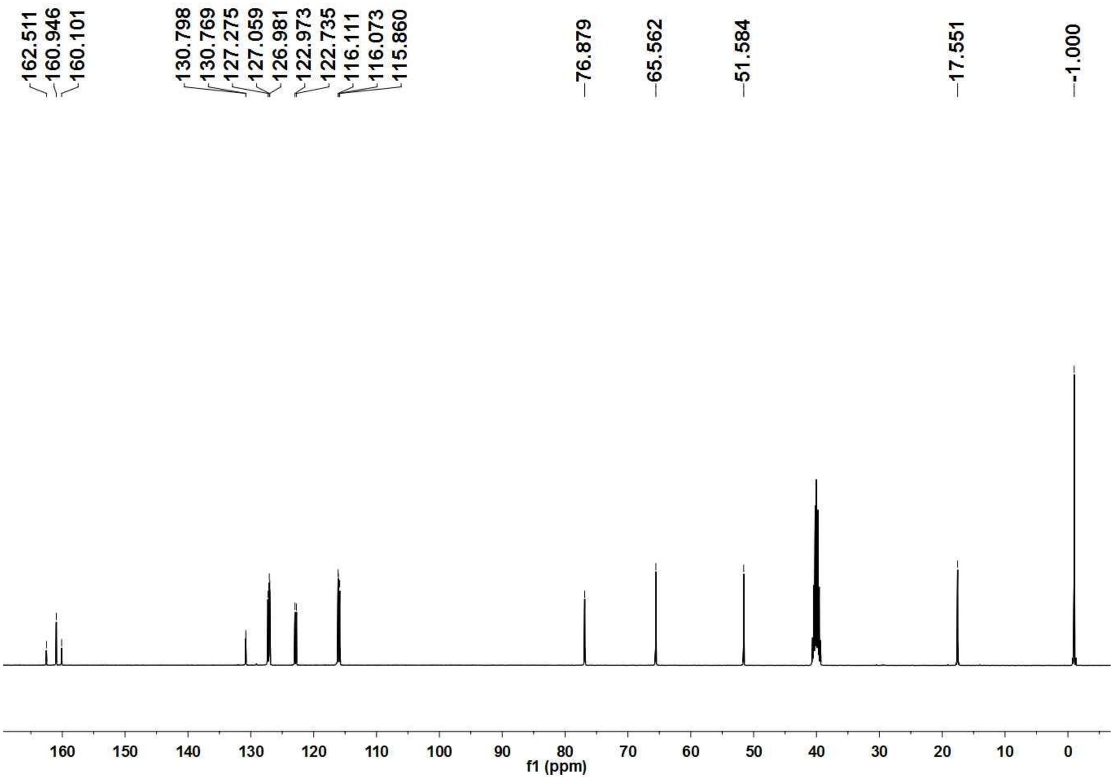

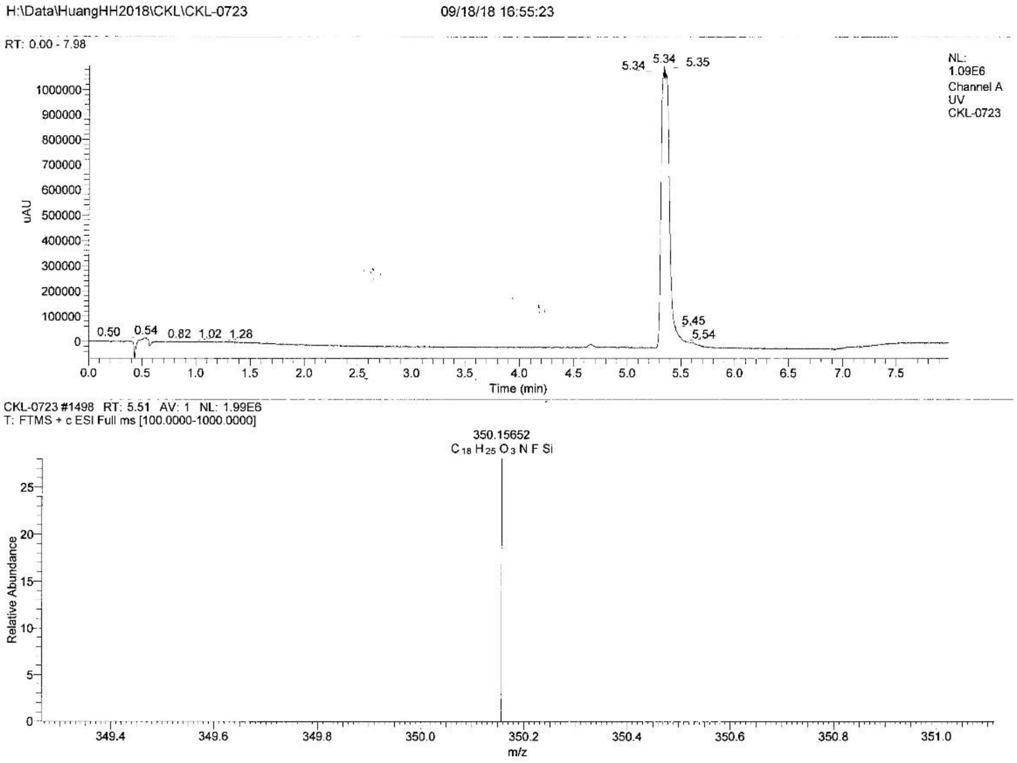

H:\Data\HuangHH2018\CKL\CKL-0723
09/18/18 16:55:23
RT: 0.00 - 7.98
NL: 1.09E6 Channel A UV CKL-0723
5.34 5.34 5.35
0.50 0.54 0.82 1.02 1.28
5.45 5.54
uAU
Time (min)
CKL-0723 #1498 RT: 5.51 AV: 1 NL: 1.99E6
T: FTMS + c ESI Full ms [100.0000-1000.0000]
350.15652
C18 H25 O3 N F Si
Relative Abundance
m/z

Methyl 4-(2-fluorophenyl)-1-((2-(trimethylsilyl)ethoxy)methyl)-1H-pyrrole-2-carboxy late (**3f**)

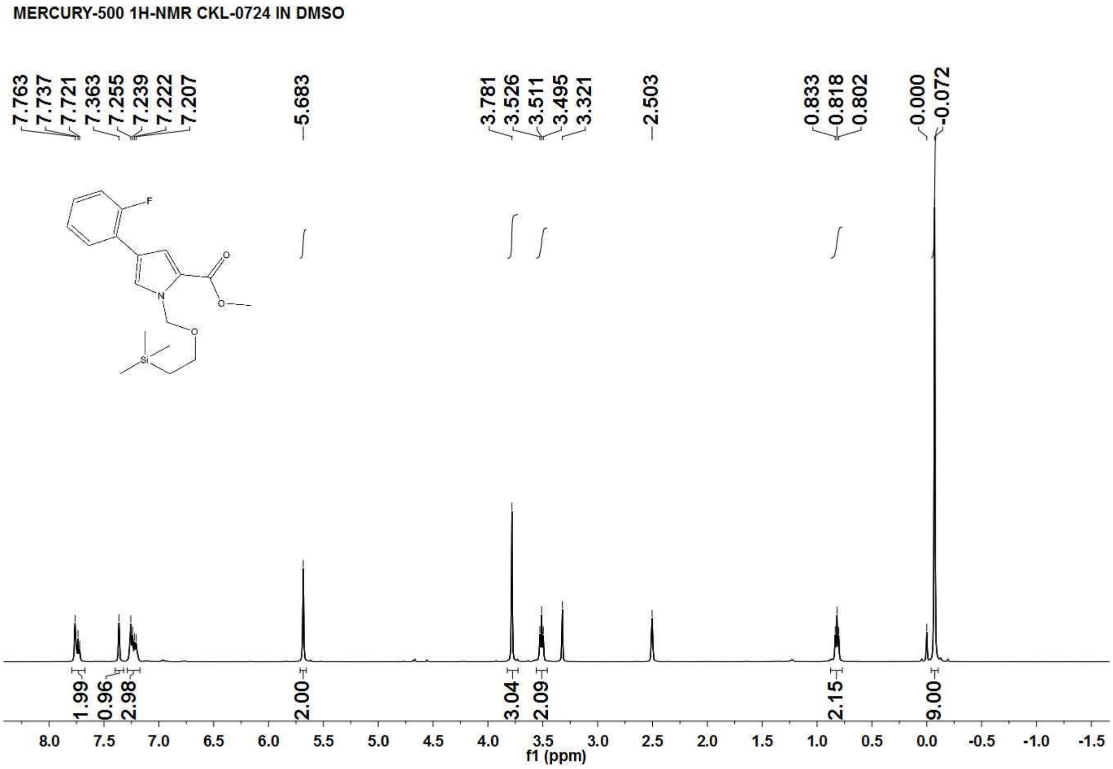

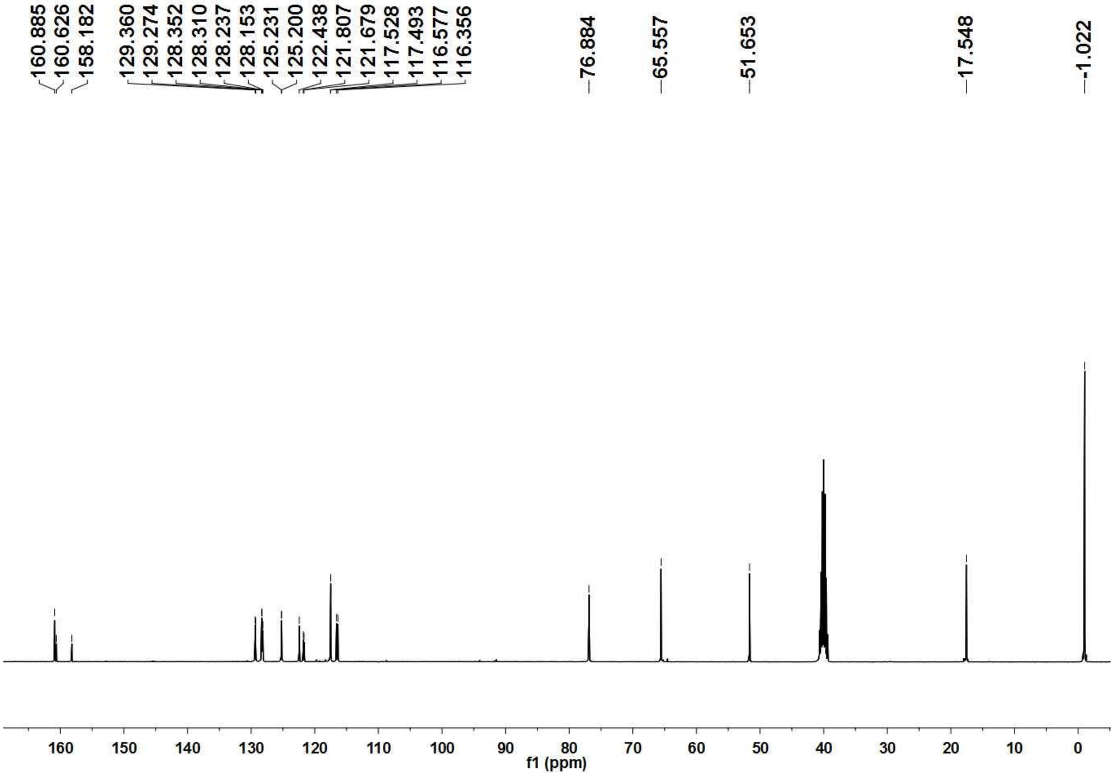

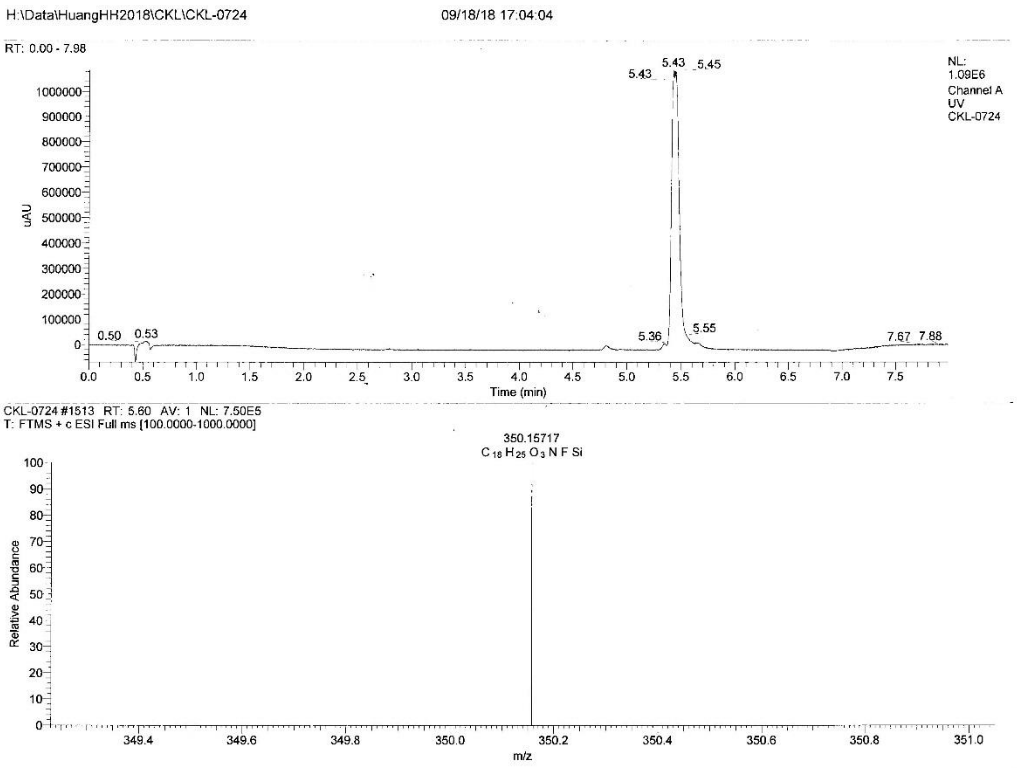
H:\Data\HuangHH2018\CKL\CKL-0724
09/18/18 17:04:04
RT: 0.00 - 7.98
NL: 1.09E6 Channel A UV CKL-0724
uAU
Time (min)
CKL-0724 #1513 RT: 5.60 AV: 1 NL: 7.50E5
T: FTMS + c ESI Full ms [100.0000-1000.0000]
350.15717
C 18 H 25 O 3 N F Si
Relative Abundance
m/z

Methyl 4-(2,4-difluorophenyl)-1-((2-(trimethylsilyl)ethoxy)methyl)-1H-pyrrole-2-carboxylate (**3g**)

MERCURY-500 1H-NMR CKL-0725 IN DMSO

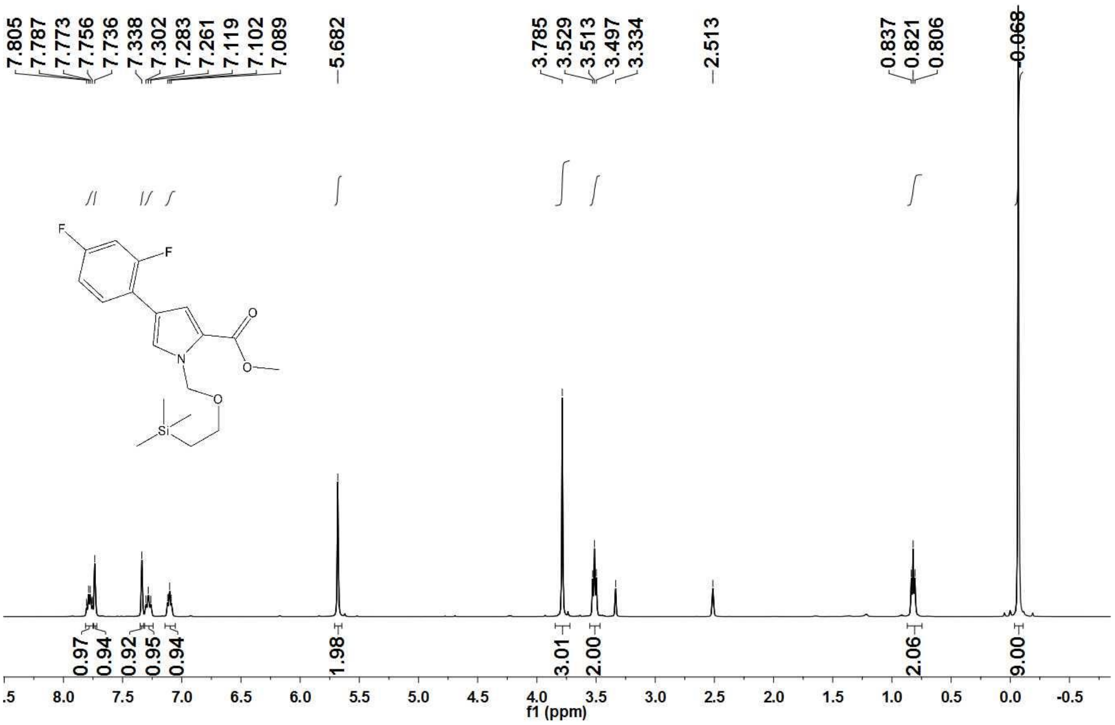

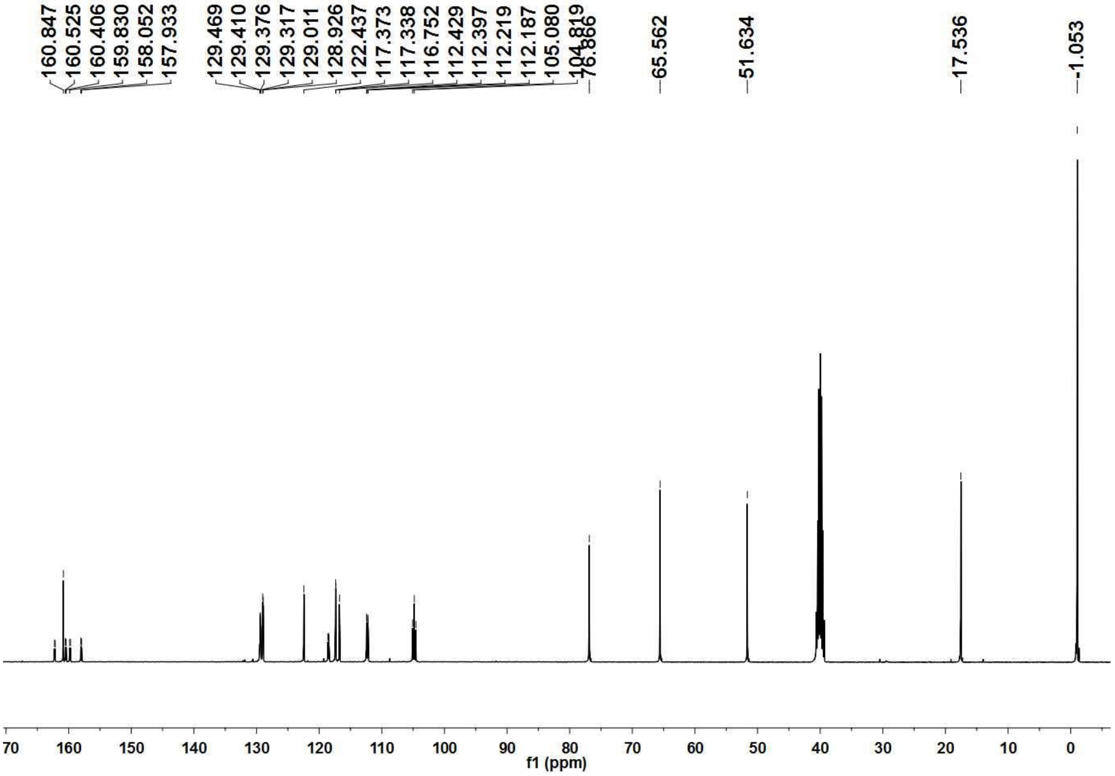

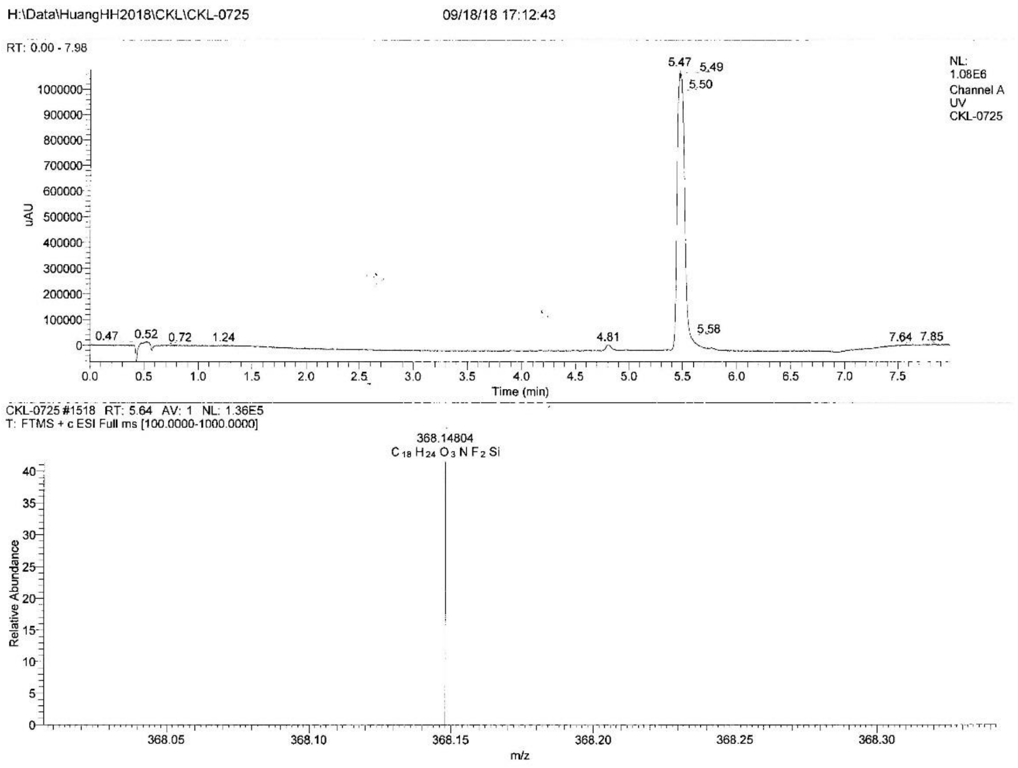
H:\Data\HuangHH2018\CKL\CKL-0725
09/18/18 17:12:43
RT: 0.00 - 7.98
NL: 1.08E6 Channel A UV CKL-0725
5.47
5.49
5.50
5.58
4.81
0.47
0.52
0.72
1.24
7.64
7.85
uAU
Time (min)
CKL-0725 #1518 RT: 5.64 AV: 1 NL: 1.36E5
T: FTMS + c ESI Full ms [100.0000-1000.0000]
368.14804
C 18 H 24 O 3 N F 2 Si
Relative Abundance
m/z

Methyl 4-(4-nitrophenyl)-1-((2-(trimethylsilyl)ethoxy)methyl)-1H-pyrrole-2-carboxylate (**3h**)

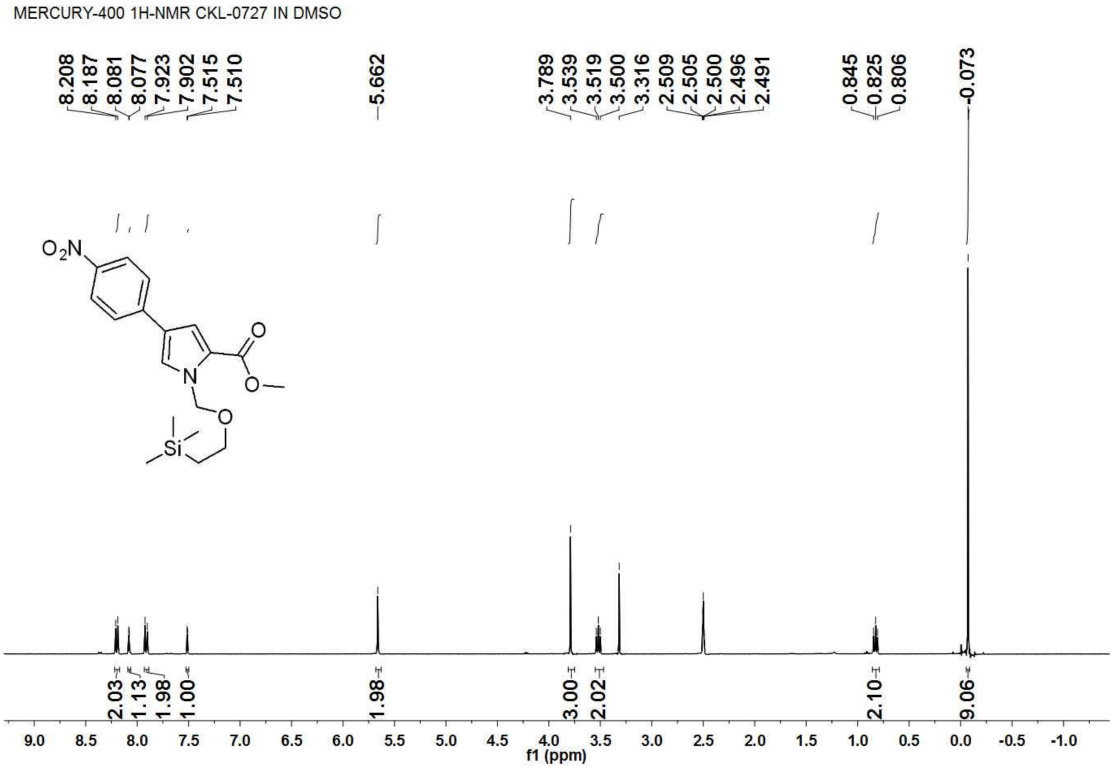

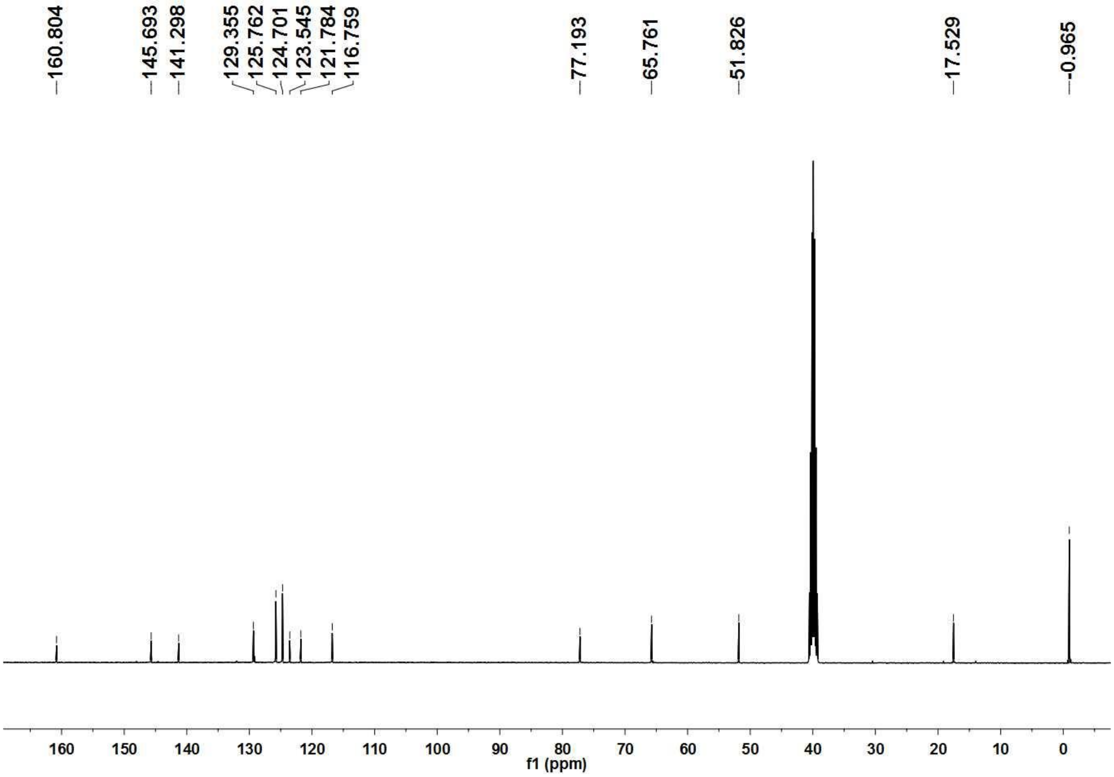

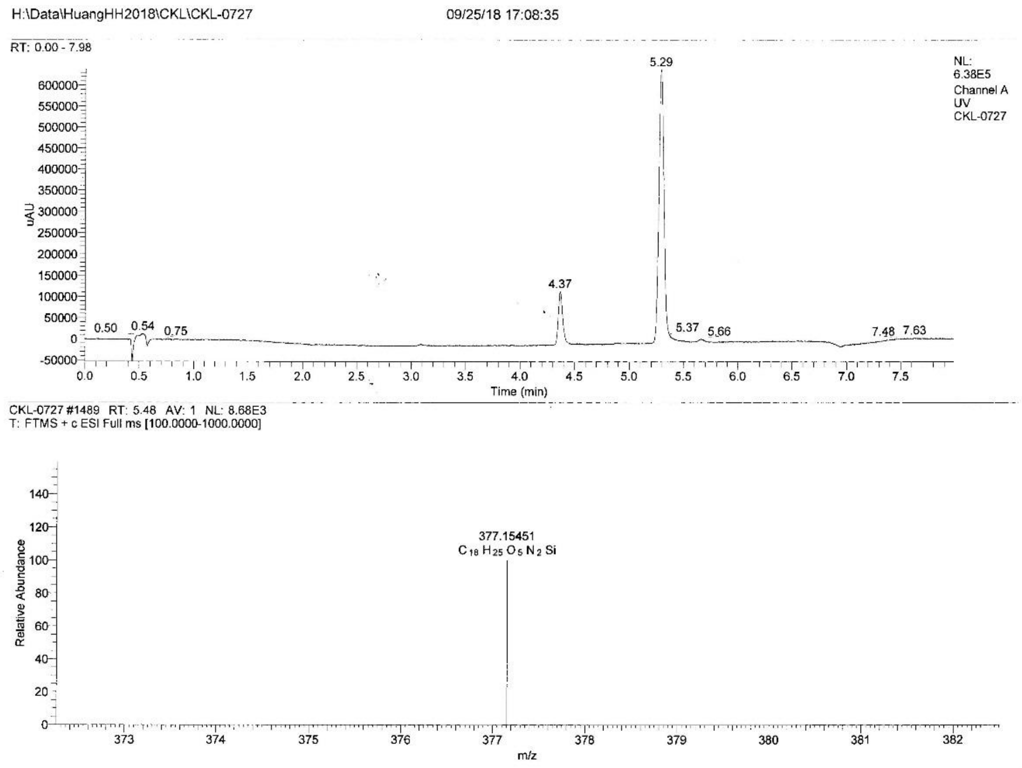
H:\Data\HuangHH2018\CKL\CKL-0727
09/25/18 17:08:35
RT: 0.00 - 7.98
NL: 6.38E5 Channel A UV CKL-0727
uAU
Time (min)
5.29
4.37
0.50 0.54 0.75
5.37 5.66
7.48 7.63
CKL-0727 #1489 RT: 5.48 AV: 1 NL: 8.68E3
T: FTMS + c ESI Full ms [100.0000-1000.0000]
Relative Abundance
377.15451
C18 H25 O5 N2 Si
m/z

Methyl 4-(4-(trifluoromethyl)phenyl)-1-((2-(trimethylsilyl)ethoxy)methyl)-1H-pyrrole -2-carboxylate (**3i**)

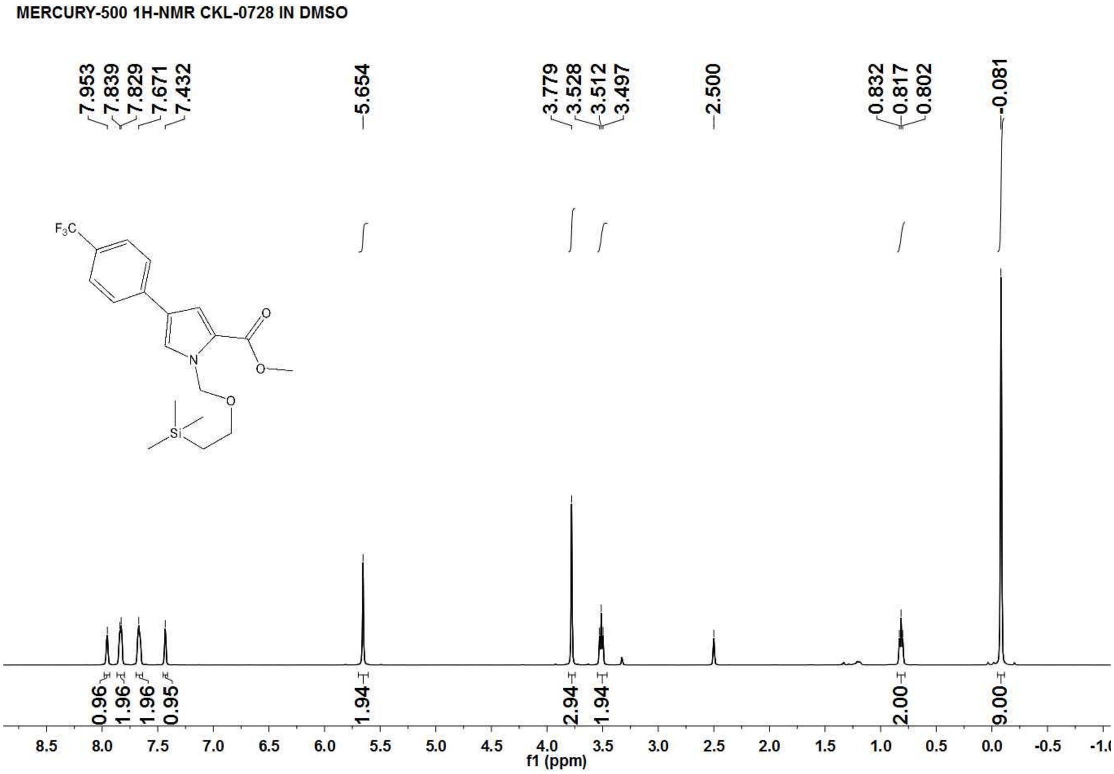

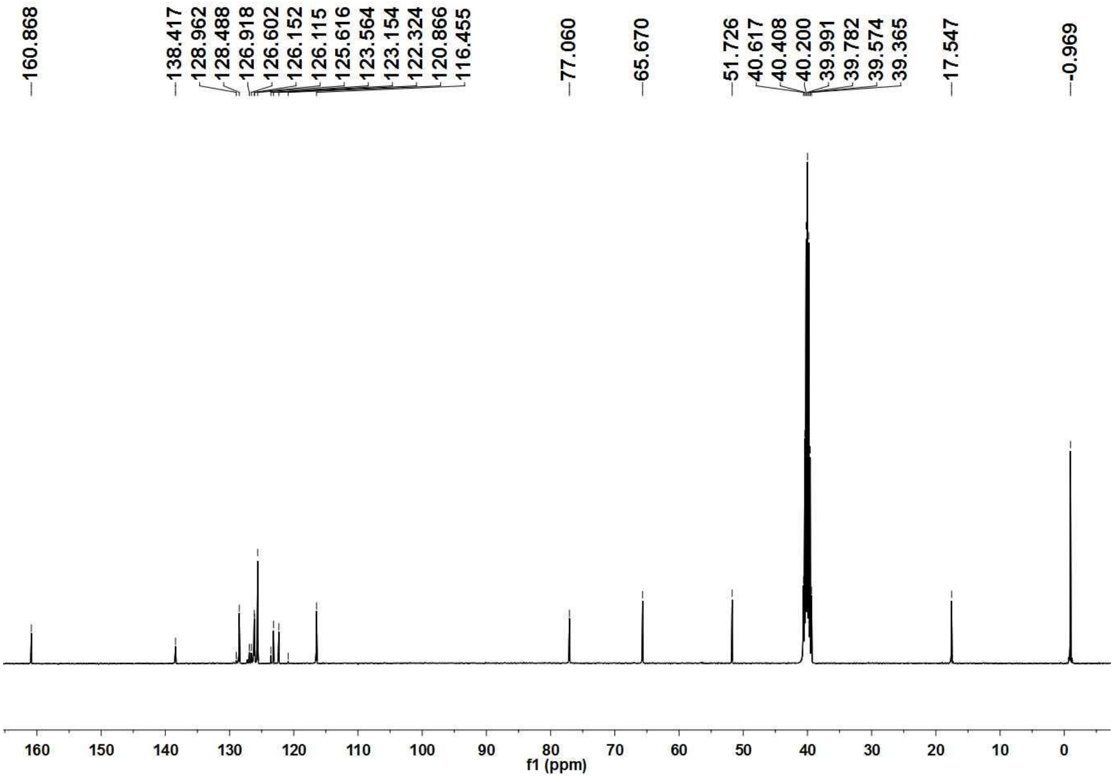

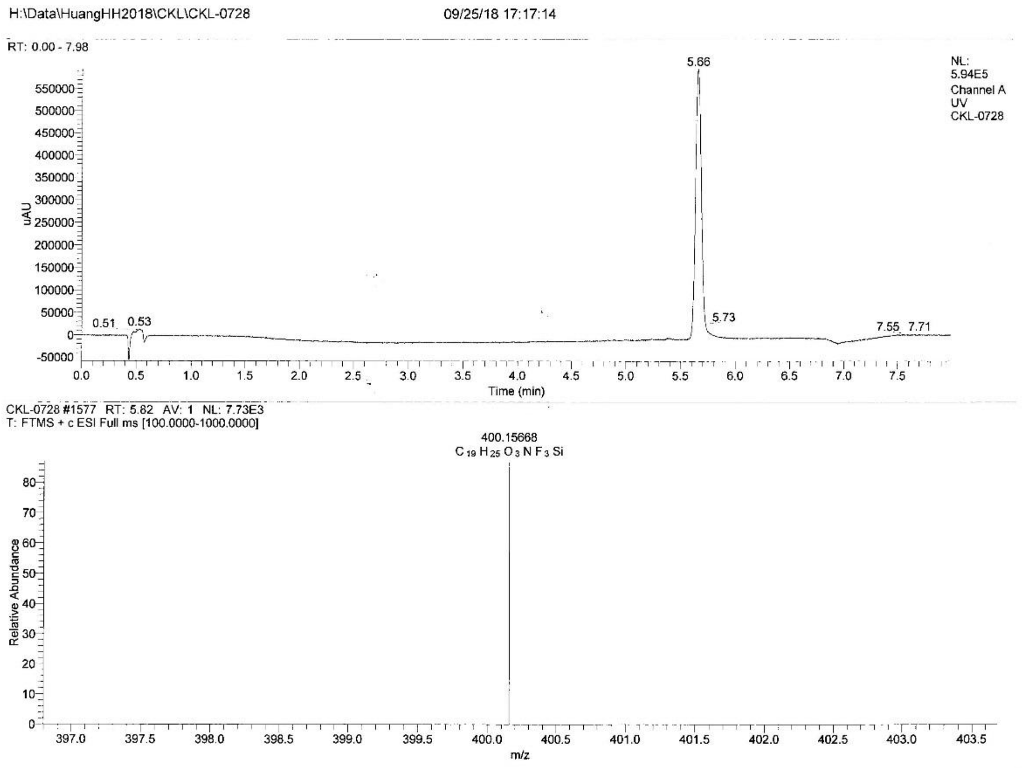

H:\Data\HuangHH2018\CKL\CKL-0728
09/25/18 17:17:14
RT: 0.00 - 7.98
5.66
NL:
5.94E5
Channel A
UV
CKL-0728
uAU
0.51
0.53
5.73
7.55
7.71
Time (min)
CKL-0728 #1577 RT: 5.82 AV: 1 NL: 7.73E3
T: FTMS + c ESI Full ms [100.0000-1000.0000]
400.15668
C 19 H 25 O 3 N F 3 Si
Relative Abundance
m/z

Methyl 4-(4-methoxyphenyl)-1-((2-(trimethylsilyl)ethoxy)methyl)-1H-pyrrole-2-carboxylate (**3j**)

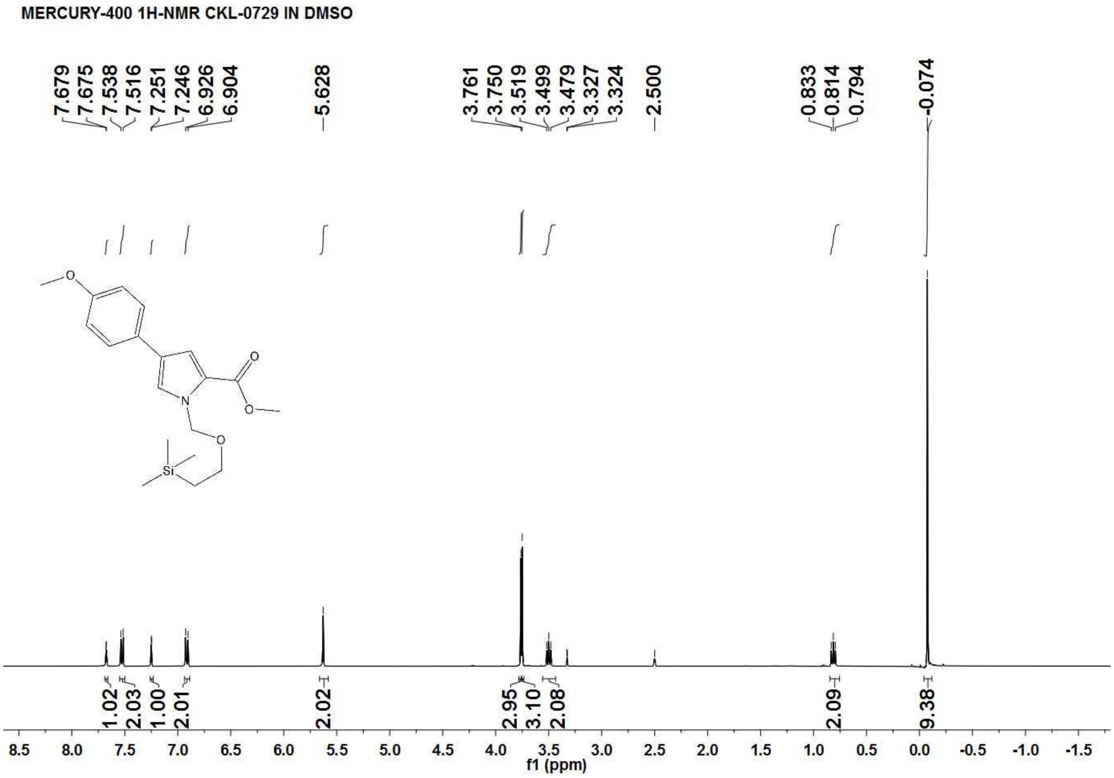

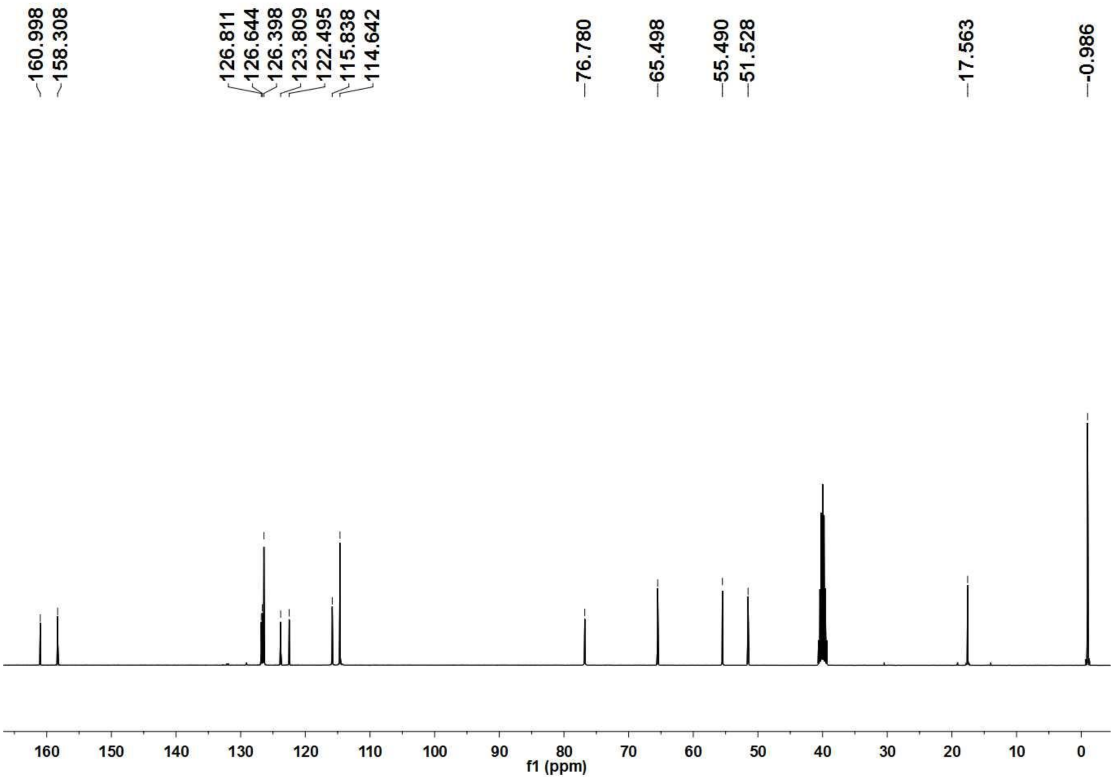

H:\Data\HuangHH2018\CKL\CKL-0729 09/25/18 17:25:54

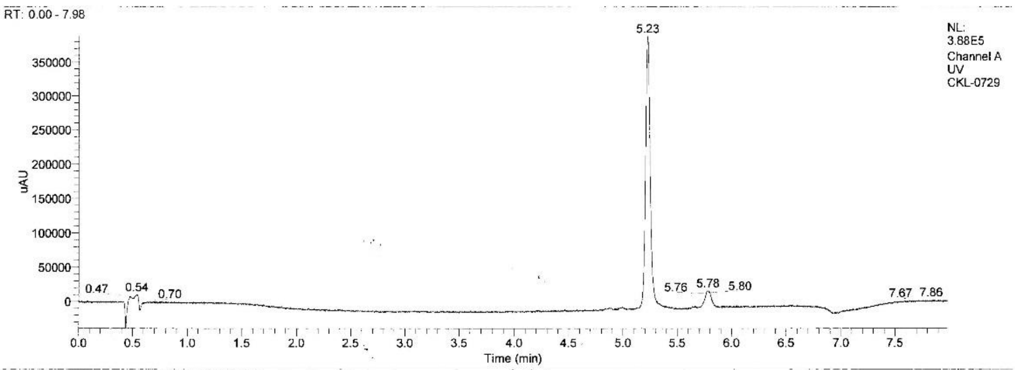

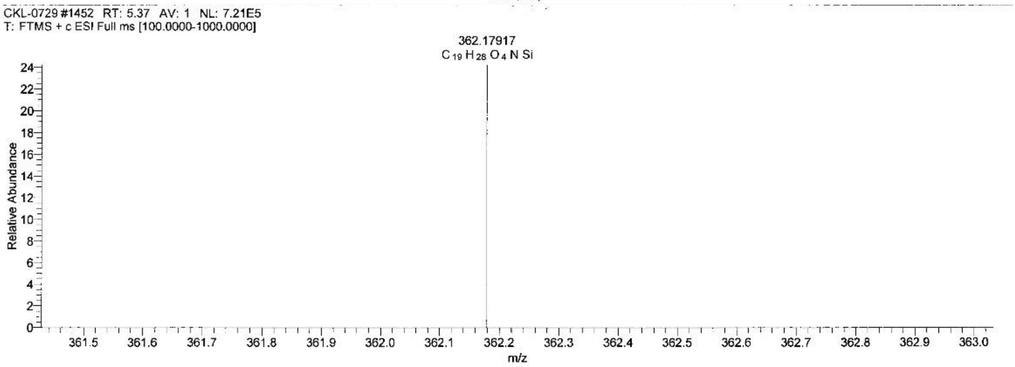

Methyl 4-(2-methoxyphenyl)-1-((2-(trimethylsilyl)ethoxy)methyl)-1H-pyrrole-2-carboxylate (**3k**)

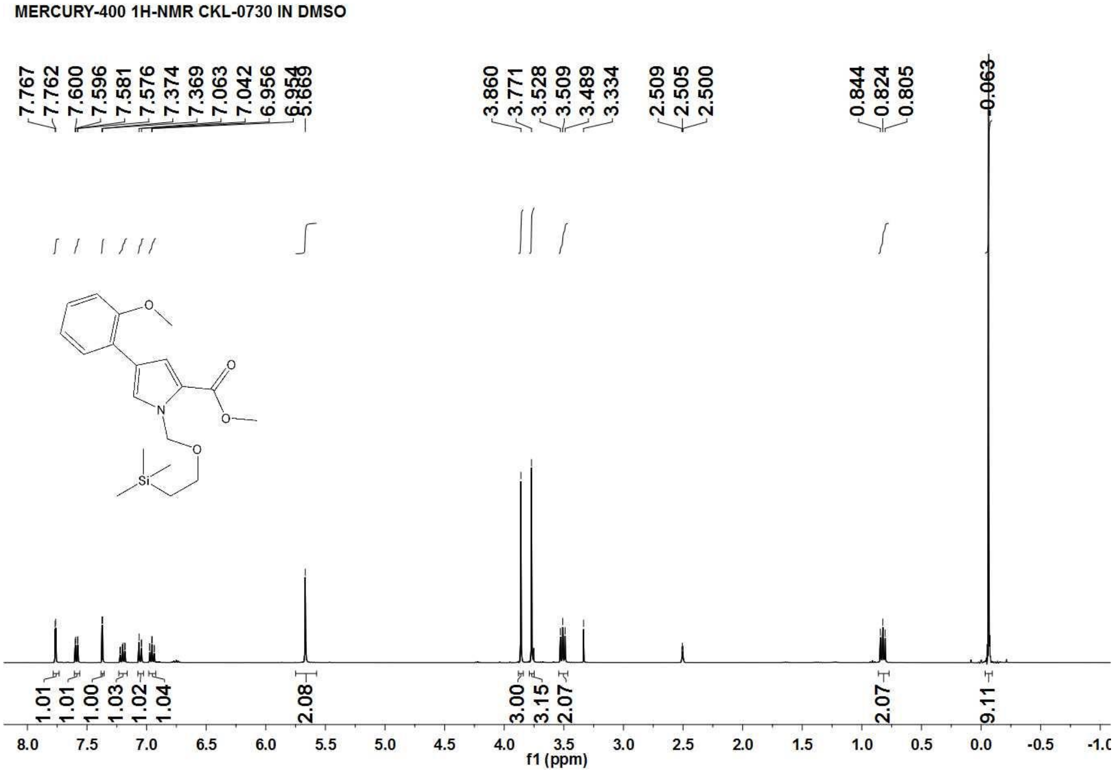

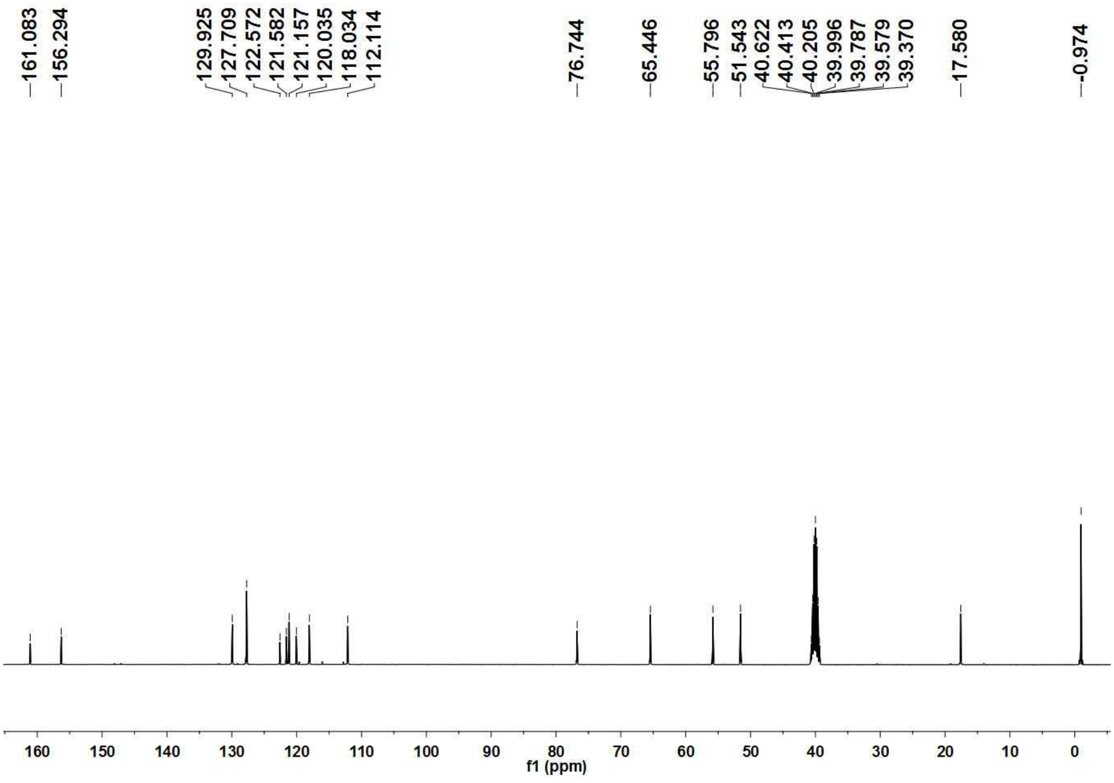

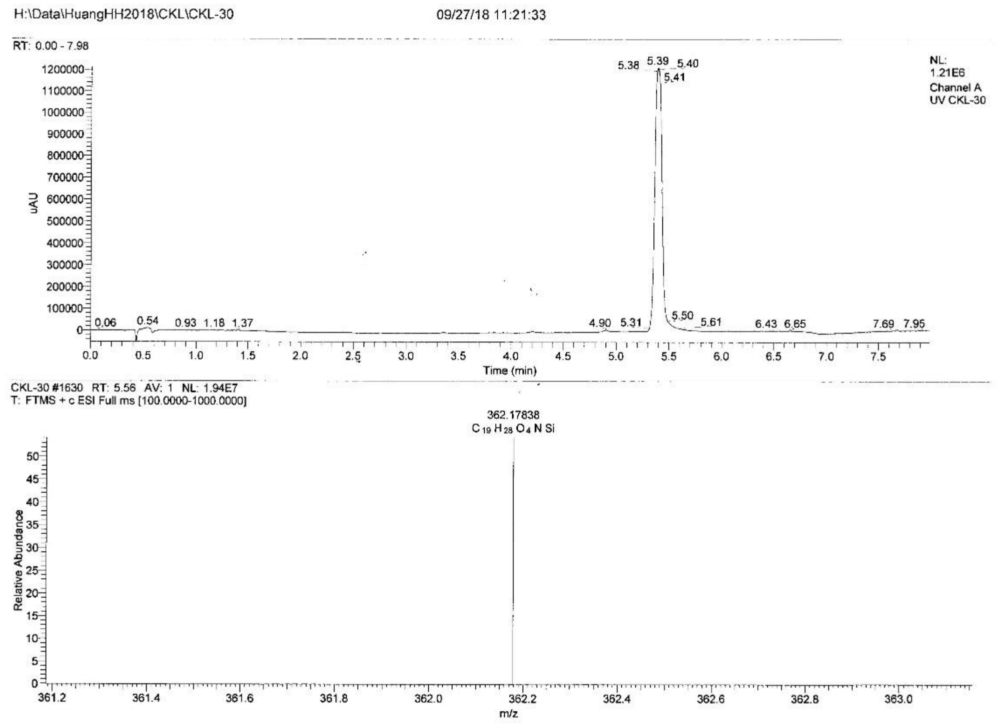
H:\Data\HuangHH2018\CKL\CKL-30
09/27/18 11:21:33
RT: 0.00 - 7.98
NL: 1.21E6 Channel A UV CKL-30
uAU
Time (min)
CKL-30 #1630 RT: 5.56 AV: 1 NL: 1.94E7
T: FTMS + c ESI Full ms [100.0000-1000.0000]
362.17838
$C_{19}H_{28}O_4NSi$
Relative Abundance
m/z

Methyl 4-(p-tolyl)-1-((2-(trimethylsilyl)ethoxy)methyl)-1H-pyrrole-2-carboxylate (**3l**)

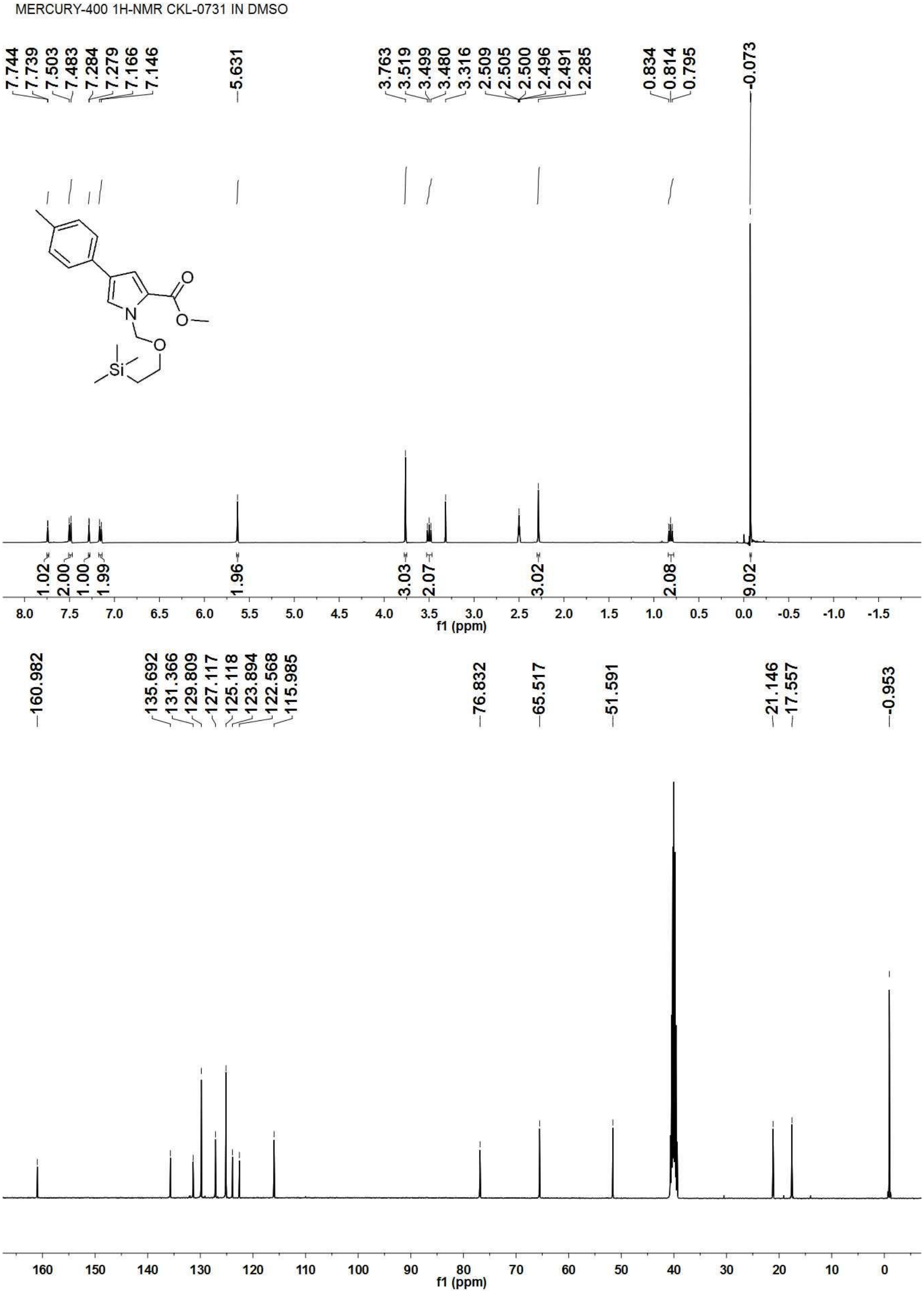

I

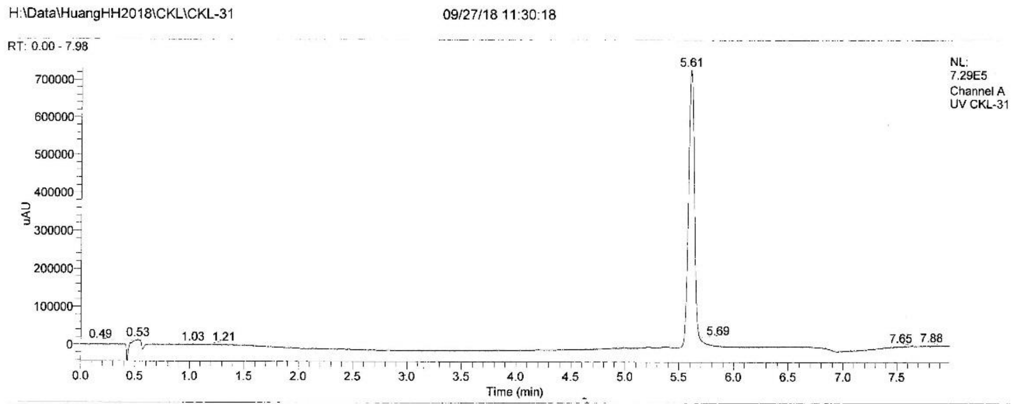
H:\Data\HuangHH2018\CKL\CKL-31
09/27/18 11:30:18
RT: 0.00 - 7.98
NL:
7.29E5
Channel A
UV CKL-31
5.61
0.49 0.53 1.03 1.21
5.69
7.65 7.88
uAU
Time (min)

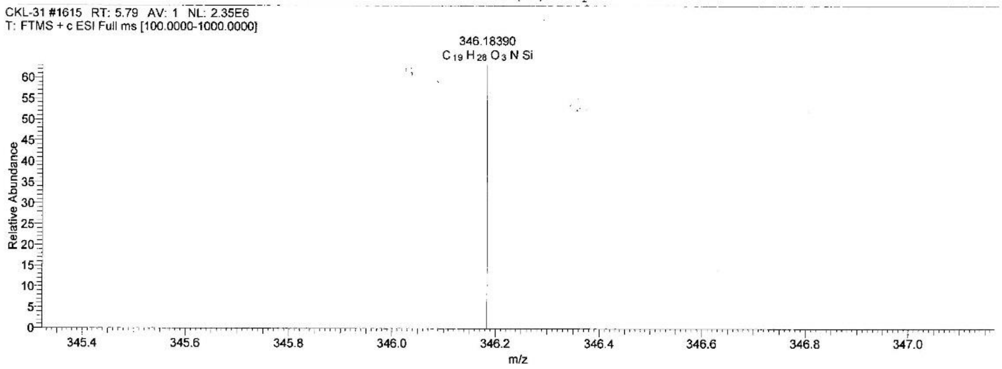
CKL-31 #1615 RT: 5.79 AV: 1 NL: 2.35E6
T: FTMS + c ESI Full ms [100.0000-1000.0000]
346.18390
C 19 H 28 O 3 N Si
Relative Abundance
m/z

Methyl 4-(o-tolyl)-1-((2-(trimethylsilyl)ethoxy)methyl)-1H-pyrrole-2-carboxylate (**3m**)

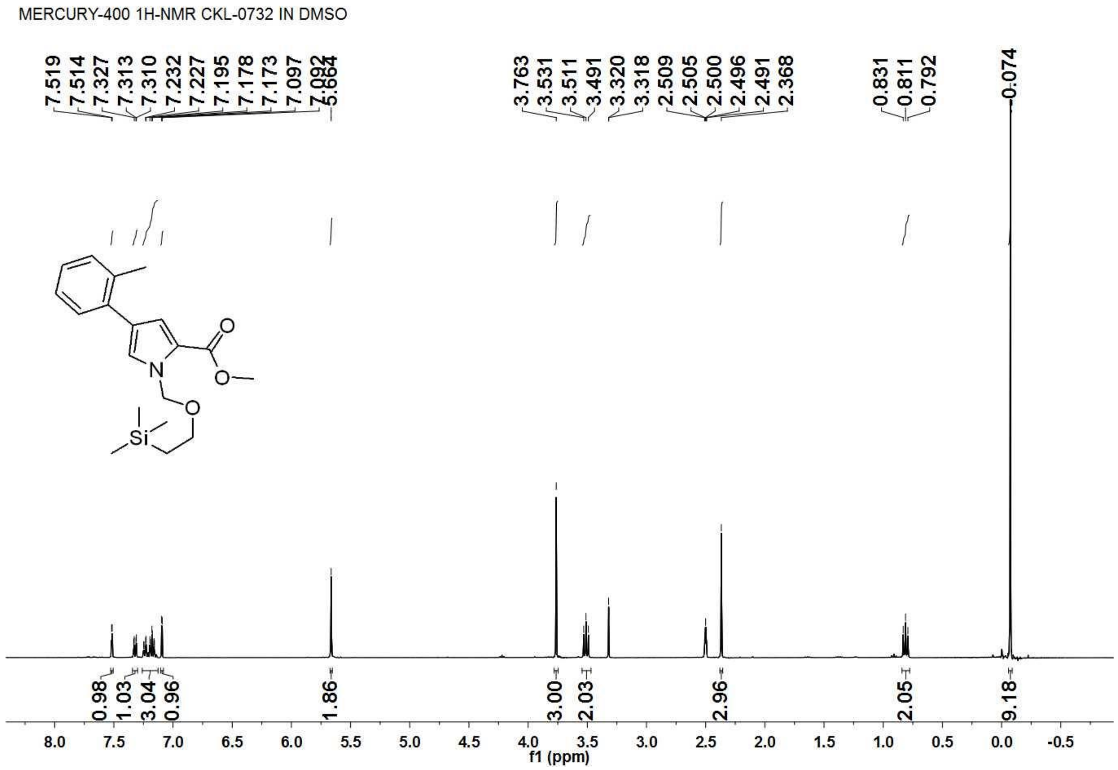

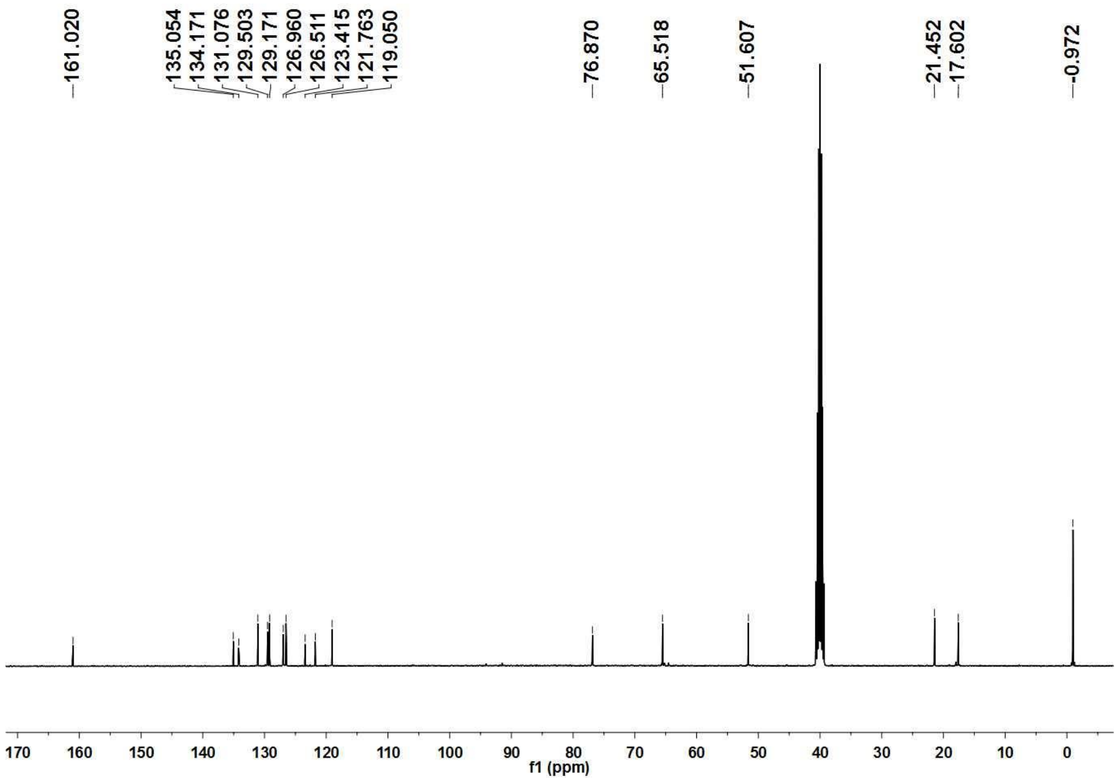

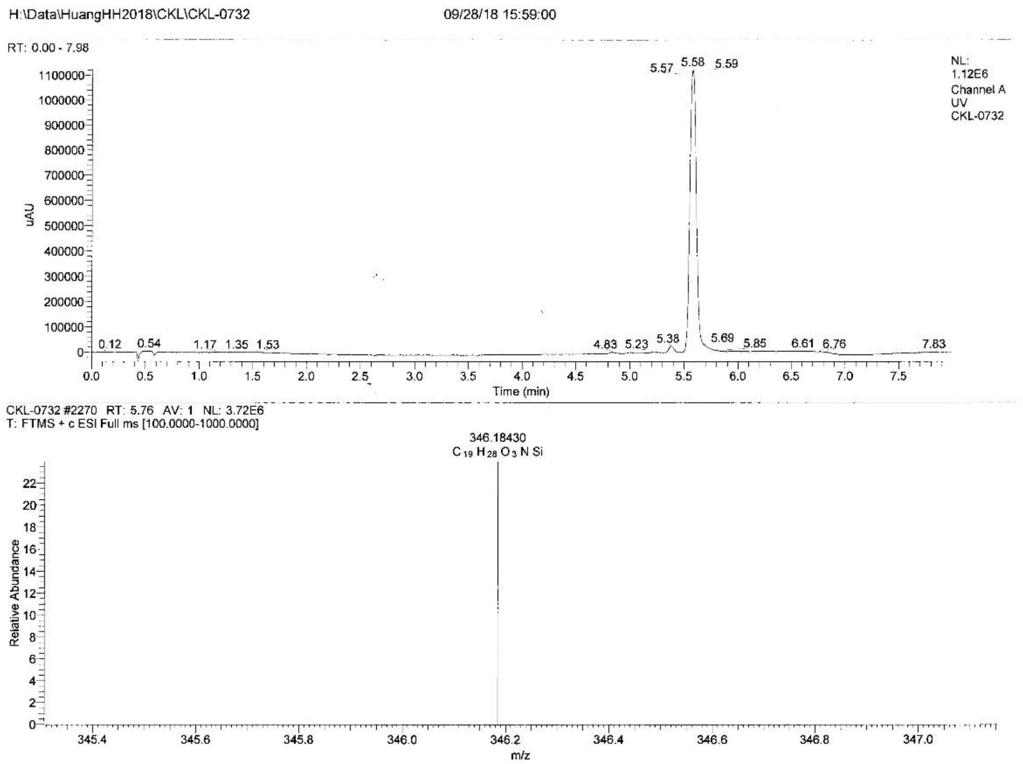
H:\Data\HuangHH2018\CKL\CKL-0732
09/28/18 15:59:00
RT: 0.00 - 7.98
NL: 1.12E6 Channel A UV CKL-0732
5.57 5.58 5.59
0.12 0.54 1.17 1.35 1.53 4.83 5.23 5.38 5.69 5.85 6.61 6.76 7.83
uAU
Time (min)
CKL-0732 #2270 RT: 5.76 AV: 1 NL: 3.72E6
T: FTMS + c ESI Full ms [100.0000-1000.0000]
346.18430
C 19 H 28 O 3 N Si
Relative Abundance
m/z

Methyl 4-(naphthalen-2-yl)-1-((2-(trimethylsilyl)ethoxy)methyl)-1H-pyrrole-2-carboxylate (**3n**)

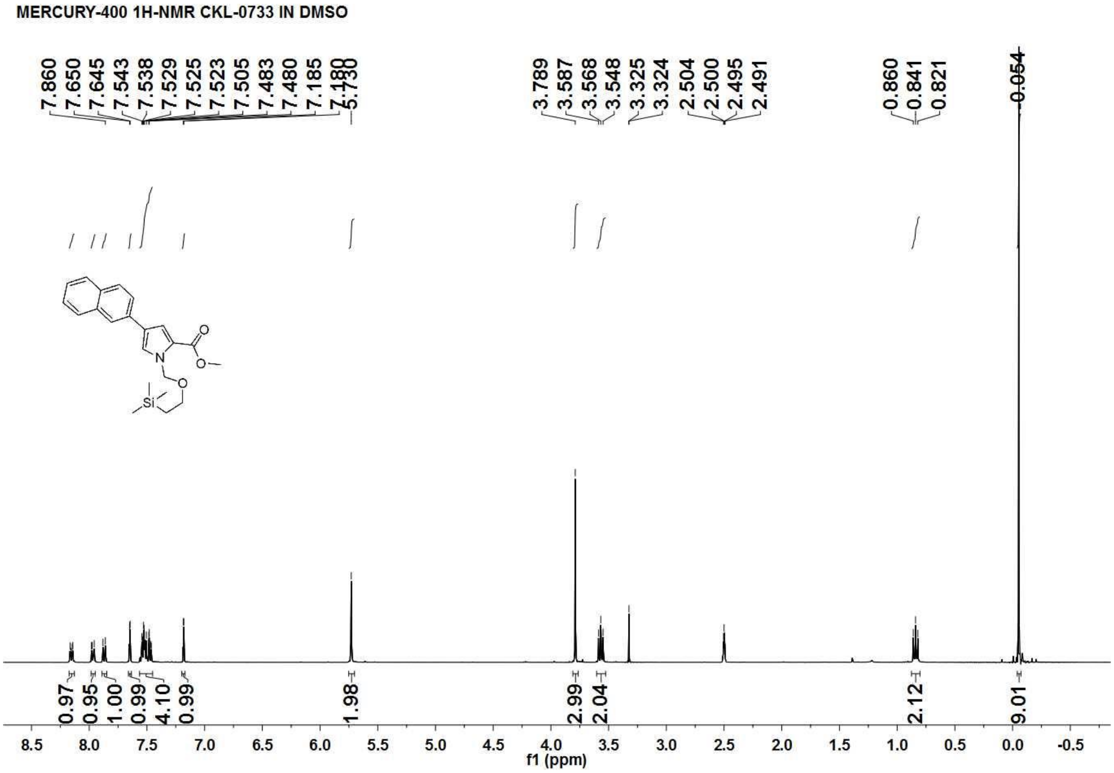

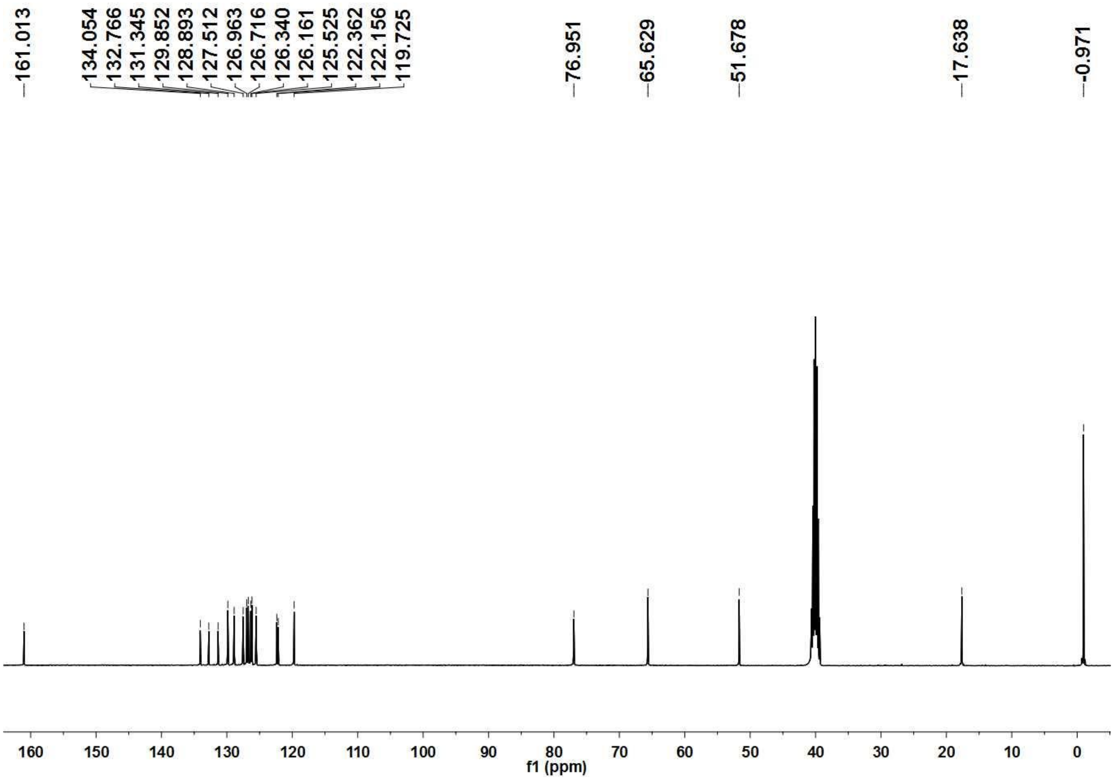

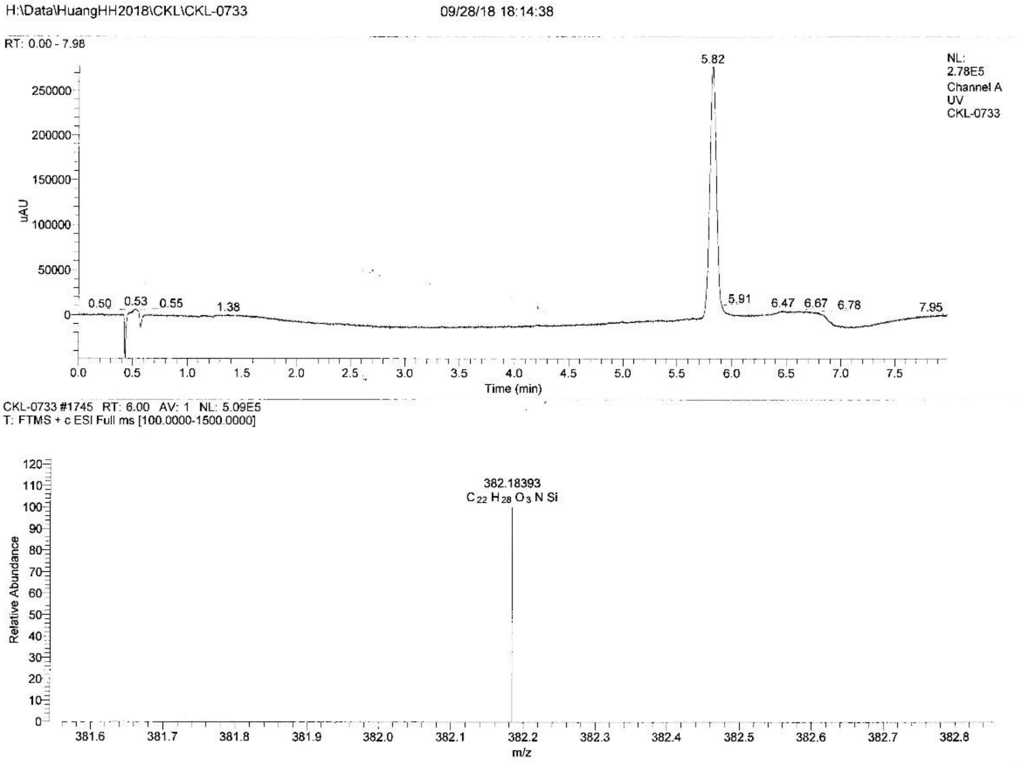
H:\Data\HuangHH2018\CKL\CKL-0733
09/28/18 18:14:38
RT: 0.00 - 7.98
NL:
2.78E5
Channel A
UV
CKL-0733
5.82
0.50
0.53
0.55
1.38
5.91
6.47
6.67
6.78
7.95
uAU
Time (min)
CKL-0733 #1745 RT: 6.00 AV: 1 NL: 5.09E5
T: FTMS + c ESI Full ms [100.0000-1500.0000]
382.18393
C 22 H 28 O 3 N Si
Relative Abundance
m/z

Methyl 4-(quinolin-3-yl)-1-((2-(trimethylsilyl)ethoxy)methyl)-1H-pyrrole-2-carboxyla te (**3o**)

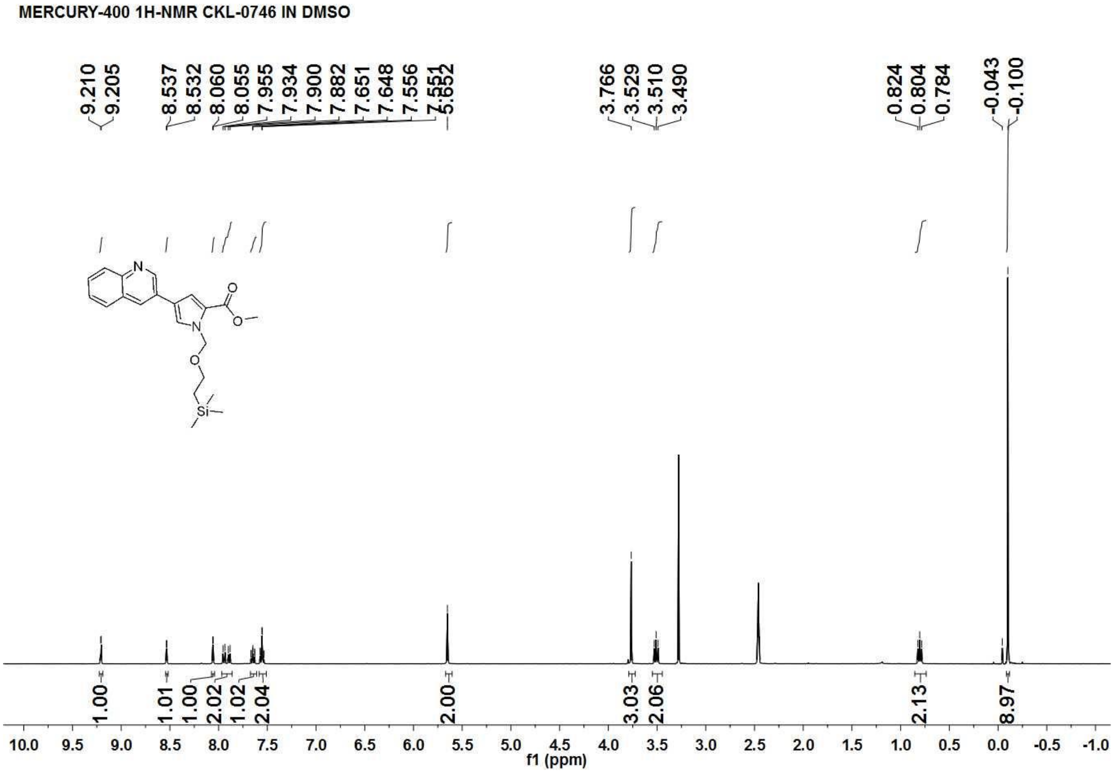

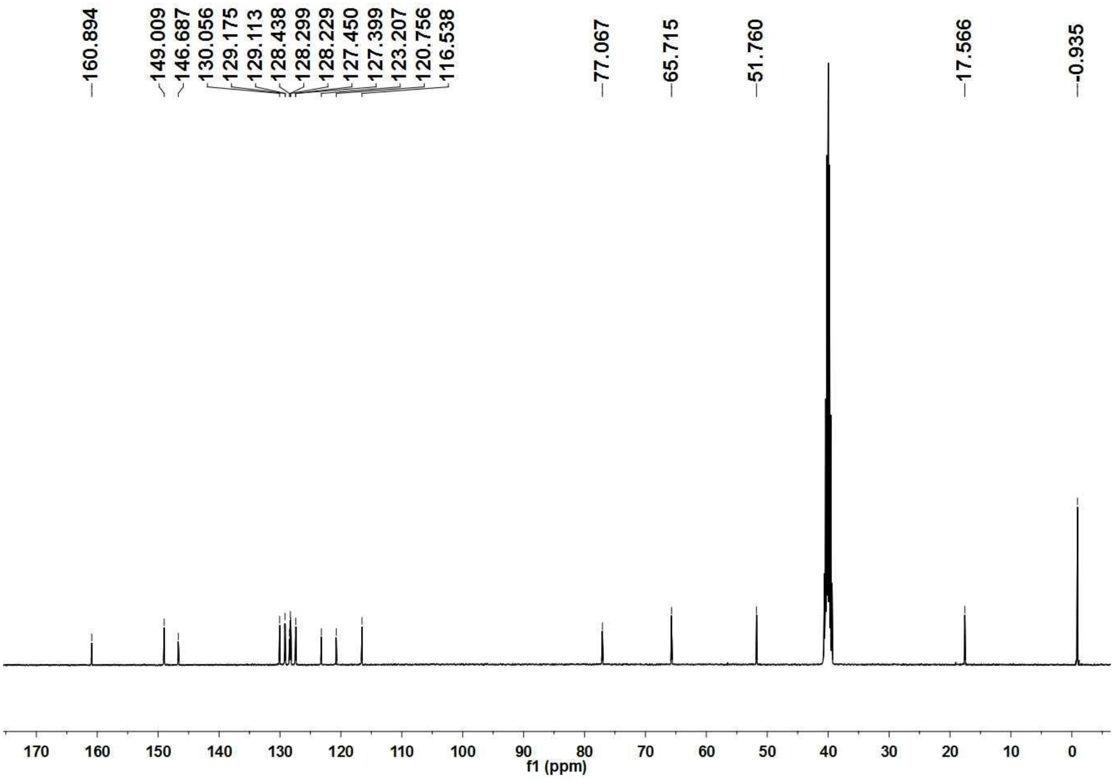

H:\Data\HuangHH2018\CKL\CKL-0746-2 10/22/18 22:38:47

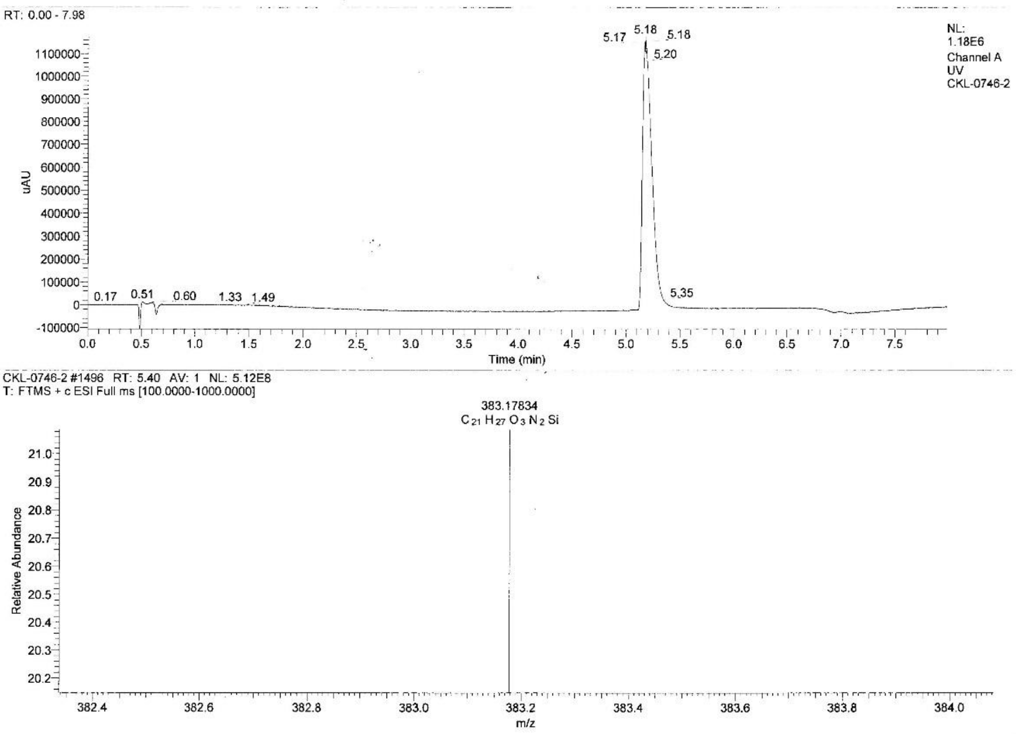

Methyl 4-(pyridin-3-yl)-1-((2-(trimethylsilyl)ethoxy)methyl)-1H-pyrrole-2-carboxylate (**3p**)

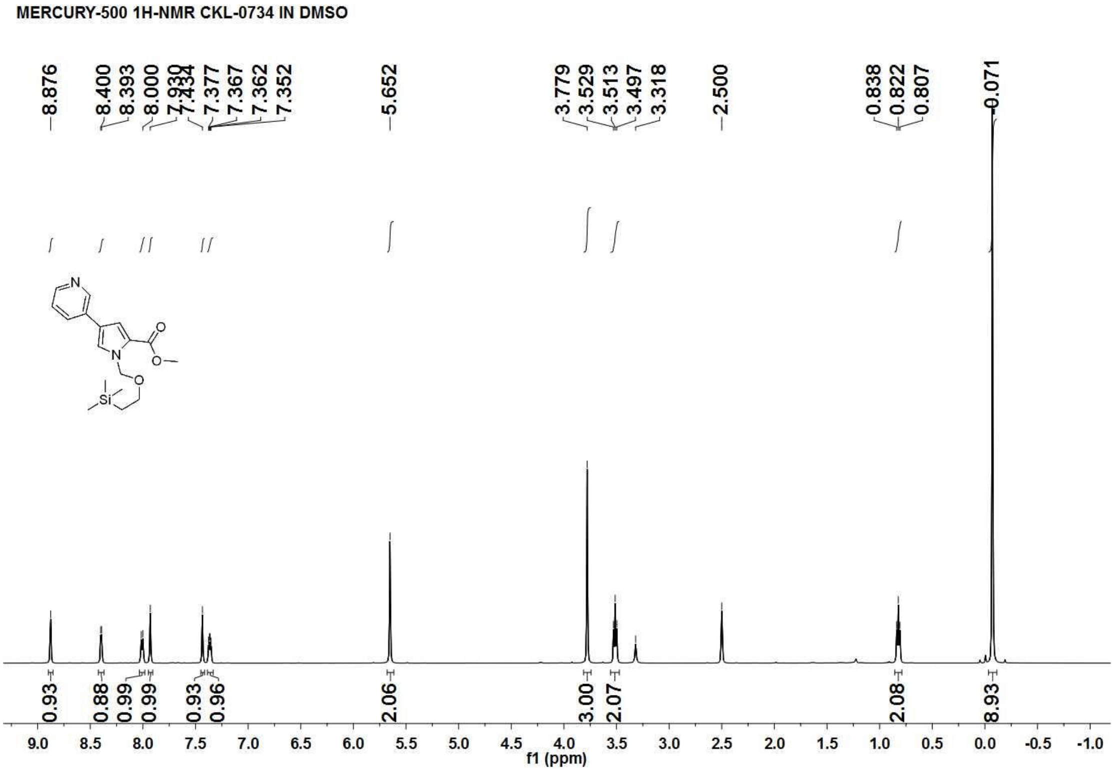

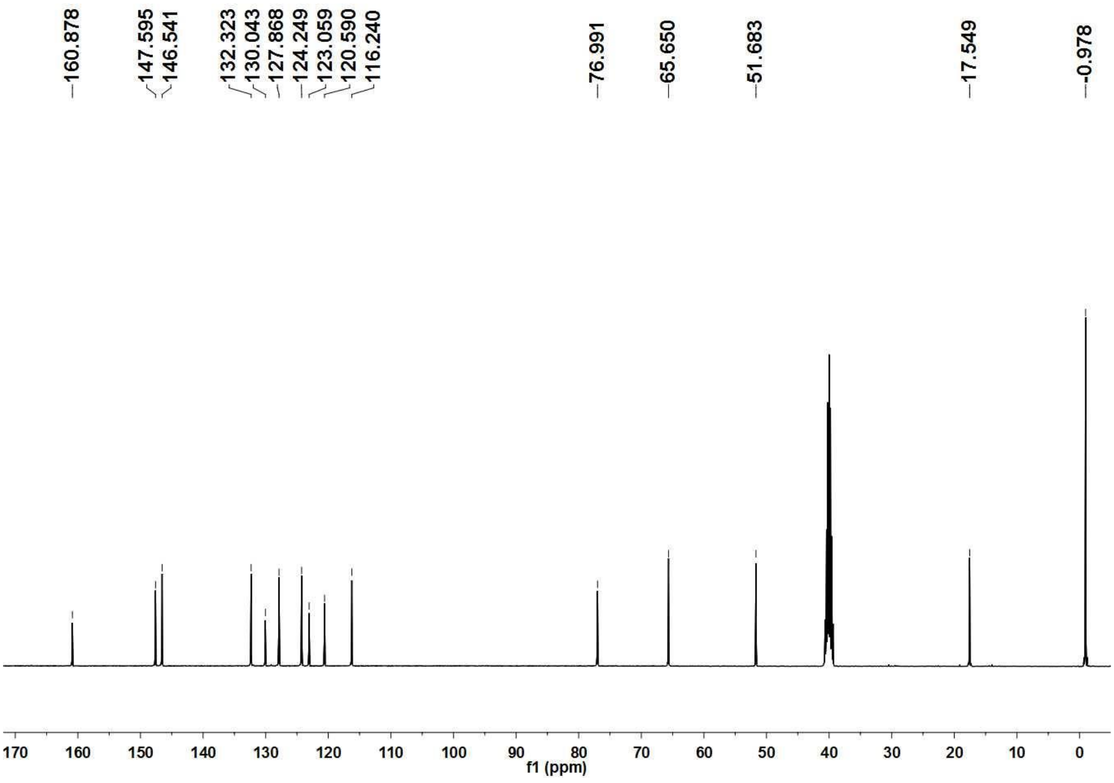

H:\Data\HuangHH2018\CKL\CKL-0734 10/12/18 10:49:38

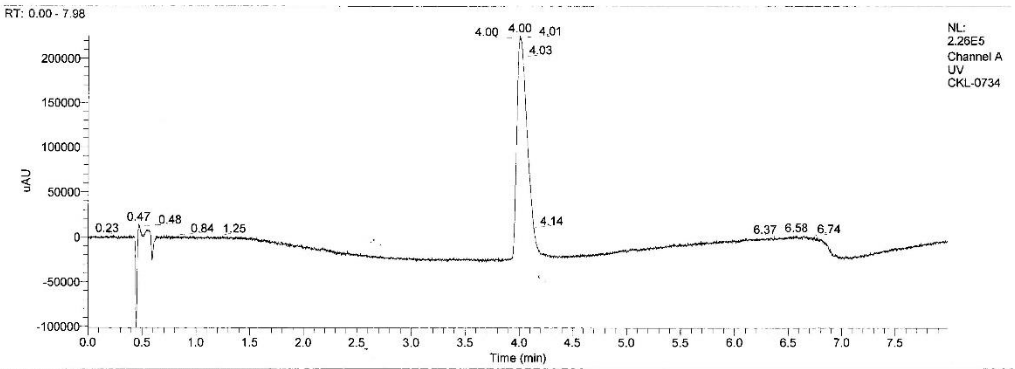

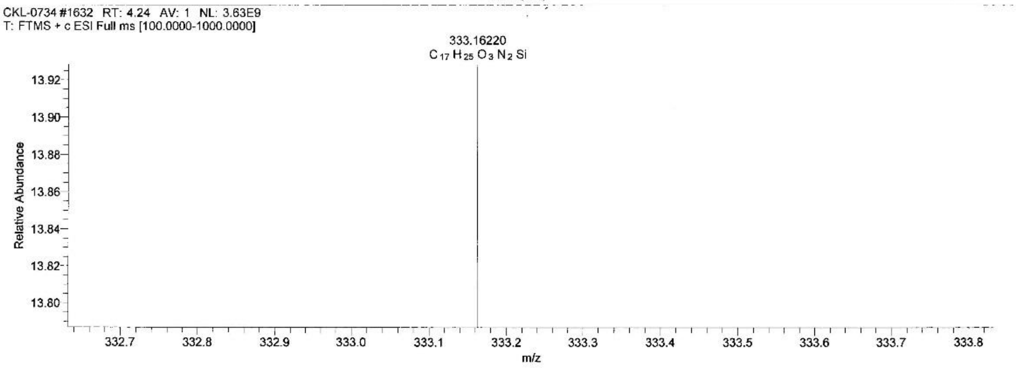

Methyl 4-(furan-2-yl)-1-((2-(trimethylsilyl)ethoxy)methyl)-1H-pyrrole-2-carboxylate (**3q**)

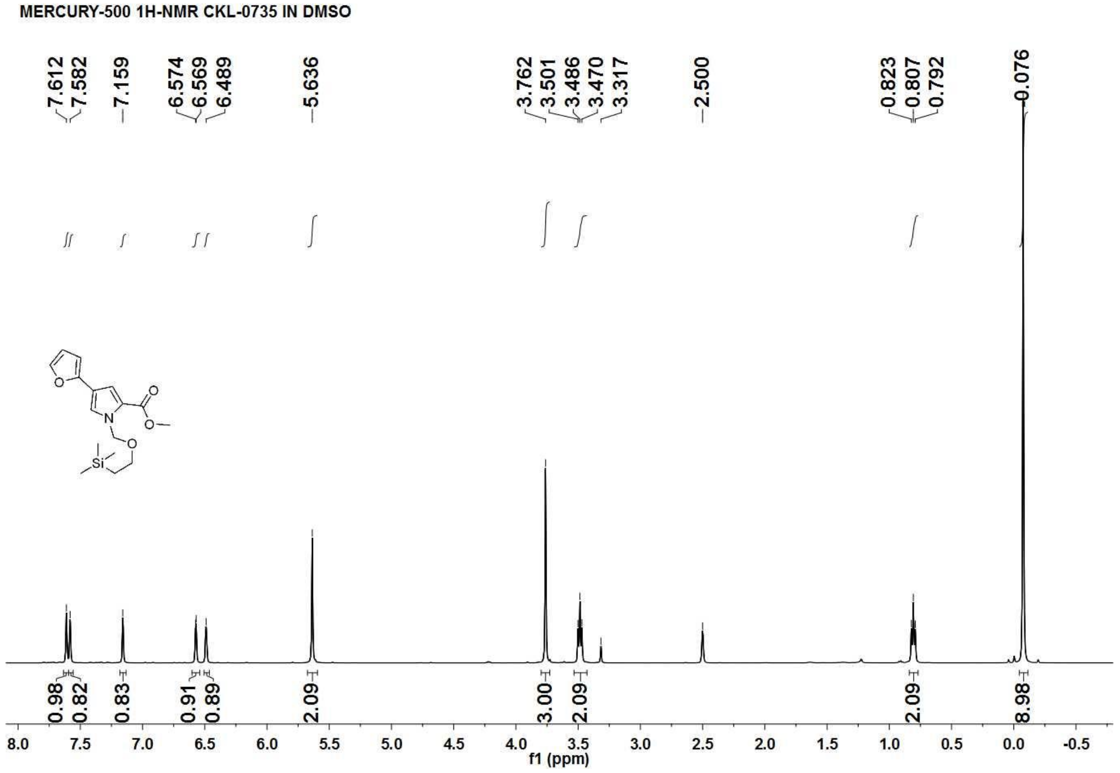

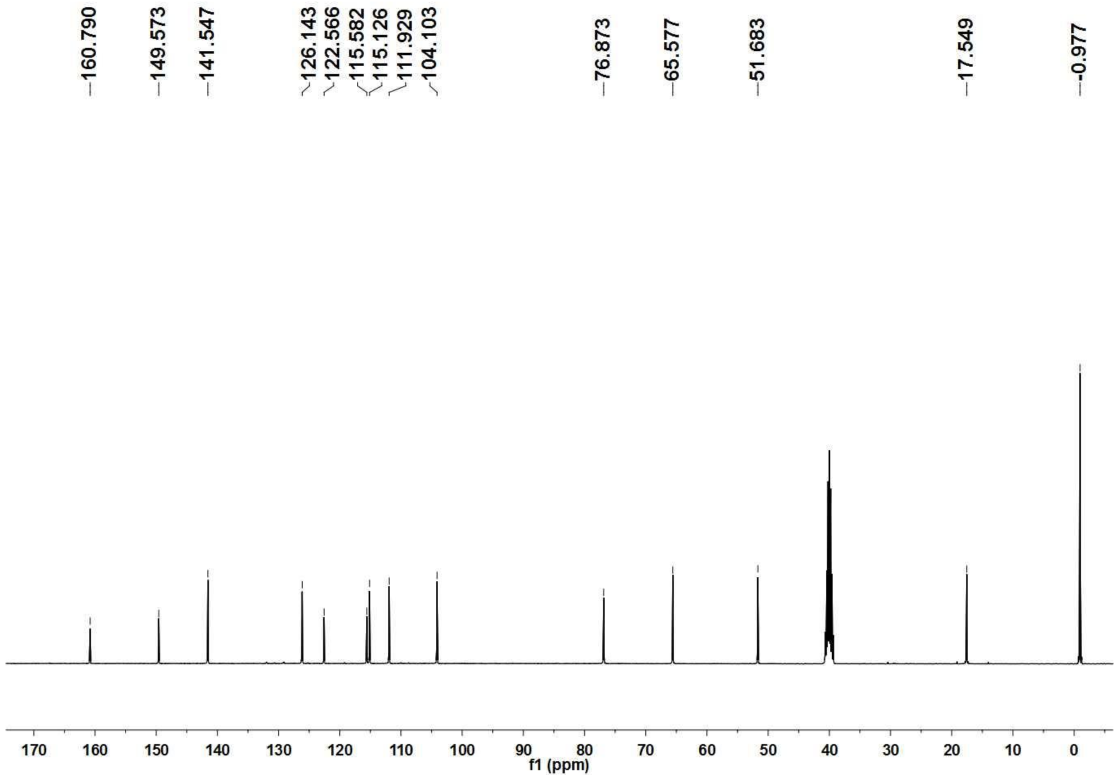

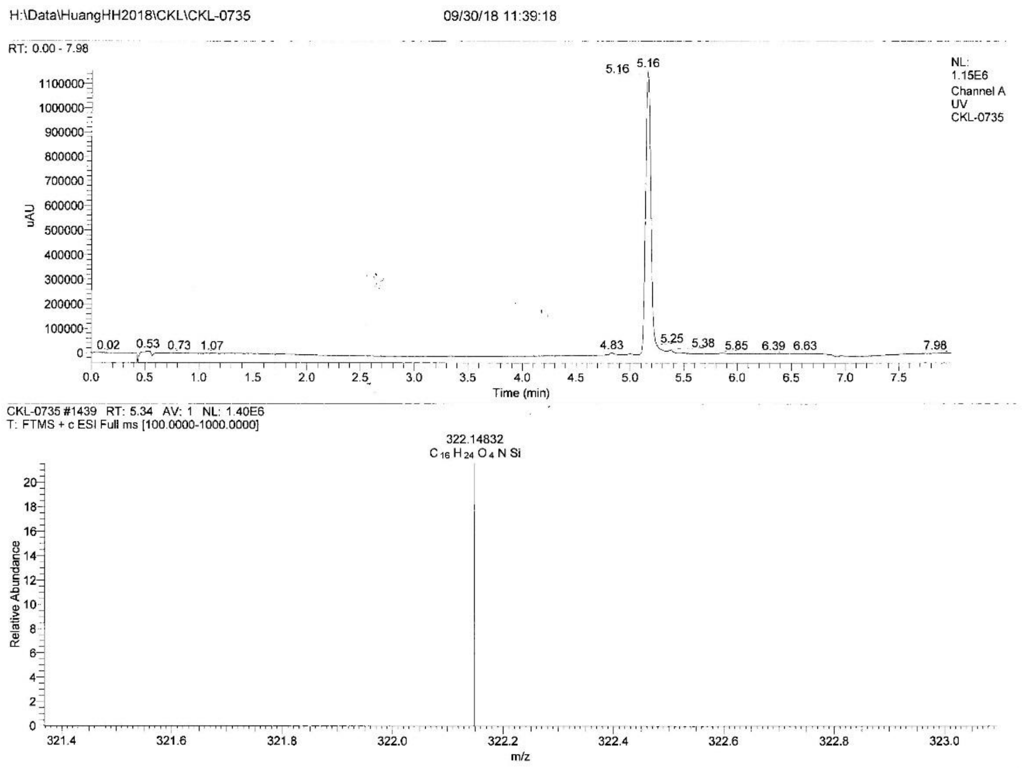
H:\Data\HuangHH2018\CKL\CKL-0735
09/30/18 11:39:18
RT: 0.00 - 7.98
NL: 1.15E6 Channel A UV CKL-0735
uAU
Time (min)
CKL-0735 #1439 RT: 5.34 AV: 1 NL: 1.40E6
T: FTMS + c ESI Full ms [100.0000-1000.0000]
322.14832
C16 H24 O4 N Si
Relative Abundance
m/z

Methyl 5-phenyl-1-((2-(trimethylsilyl)ethoxy)methyl)-1H-pyrrole-2-carboxylate (**3r**)

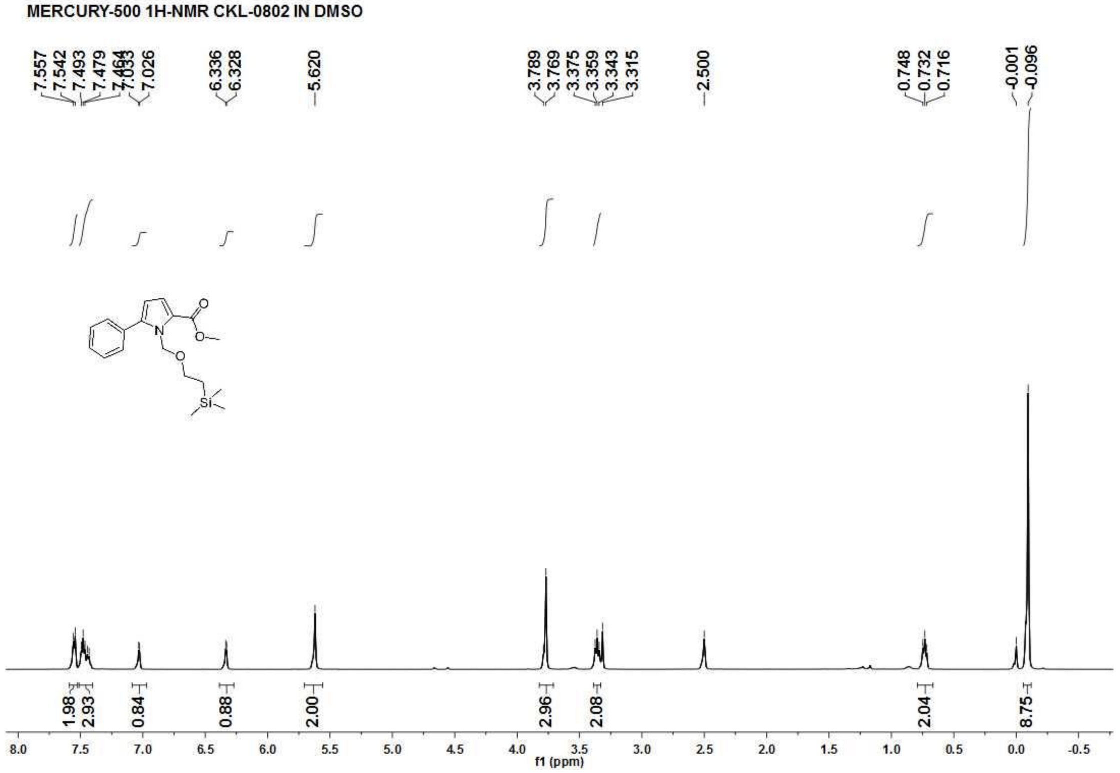

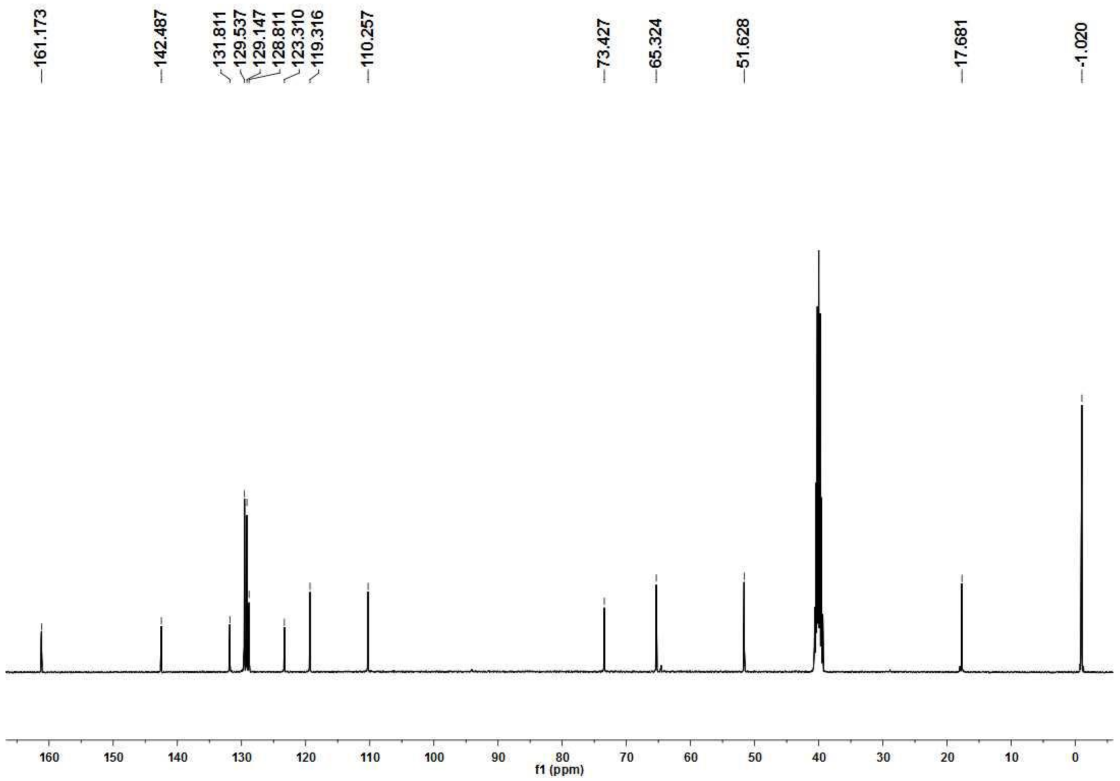

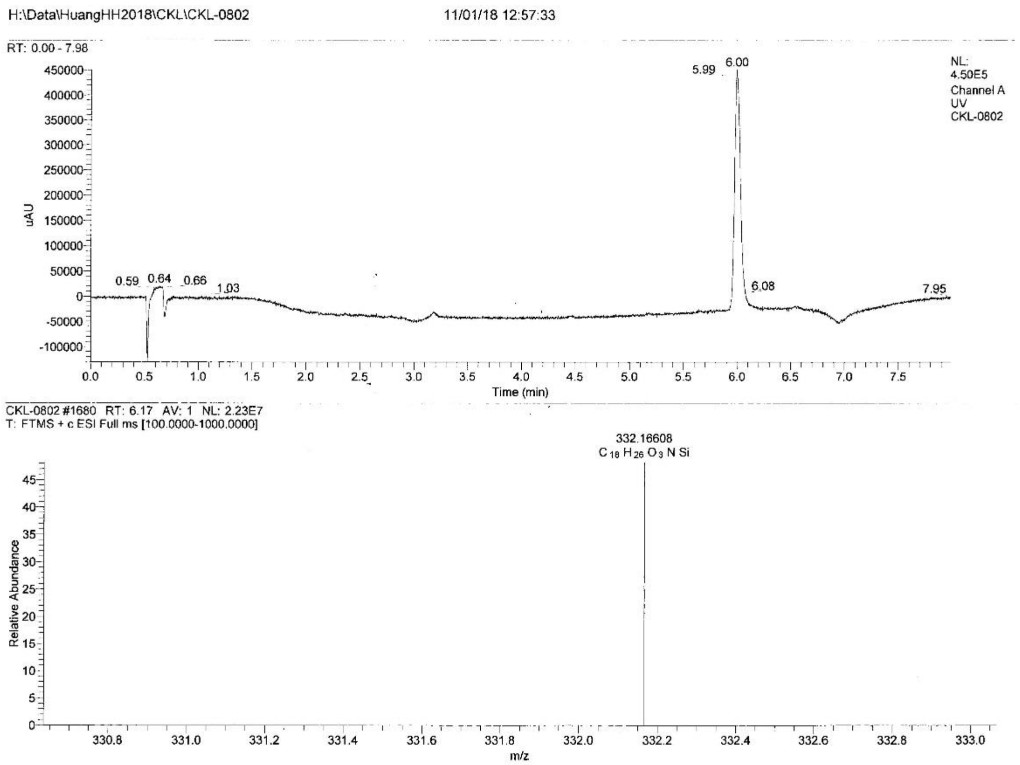
H:\Data\HuangHH2018\CKL\CKL-0802
11/01/18 12:57:33
RT: 0.00 - 7.98
NL:
4.50E5
Channel A
UV
CKL-0802
uAU
Time (min)
5.99
6.00
6.08
7.95
0.59
0.64
0.66
1.03
CKL-0802 #1680 RT: 6.17 AV: 1 NL: 2.23E7
T: FTMS + c ESI Full ms [100.0000-1000.0000]
332.16608
C 18 H 26 O 3 N Si
Relative Abundance
m/z

Methyl 3-phenyl-1-((2-(trimethylsilyl)ethoxy)methyl)-1H-pyrrole-2-carboxylate (**3s**)

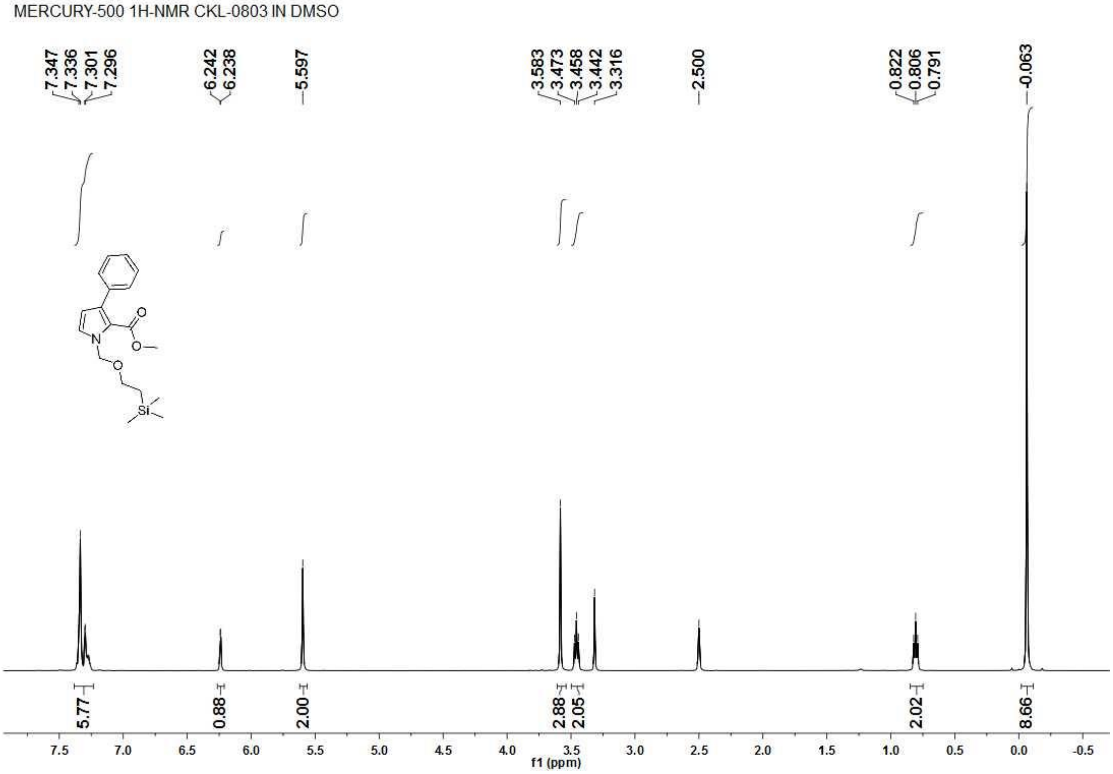

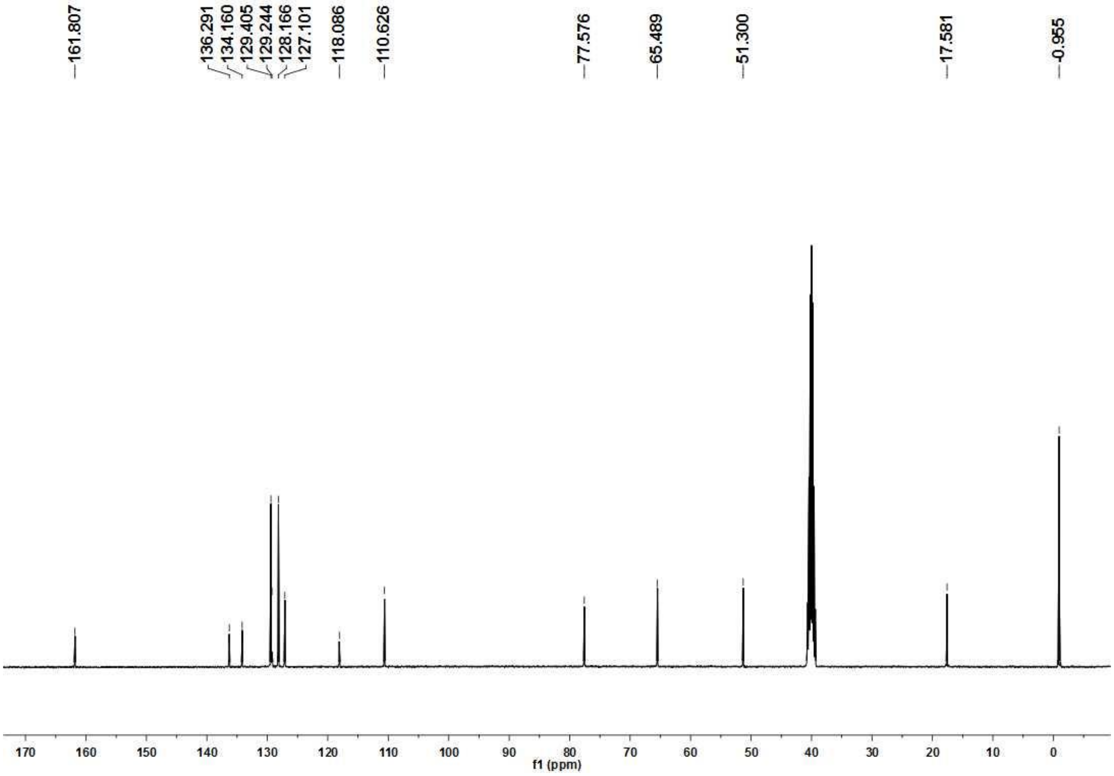

H:\Data\HuangHH2018\CKL\CKL-0803 11/01/18 13:06:07

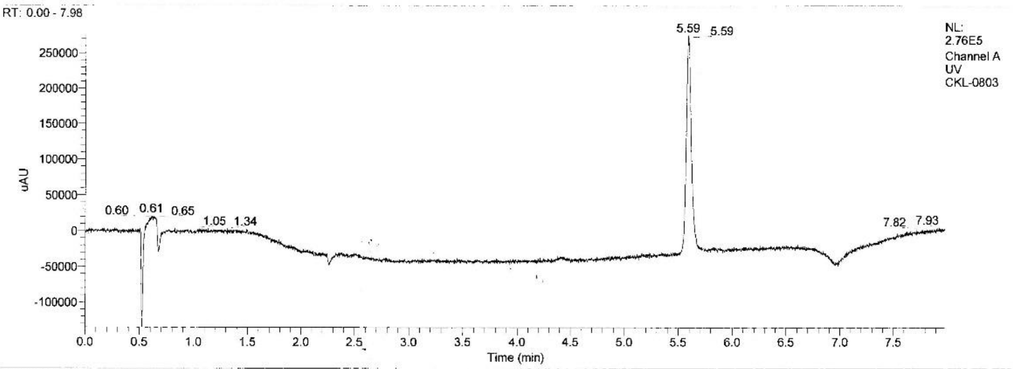

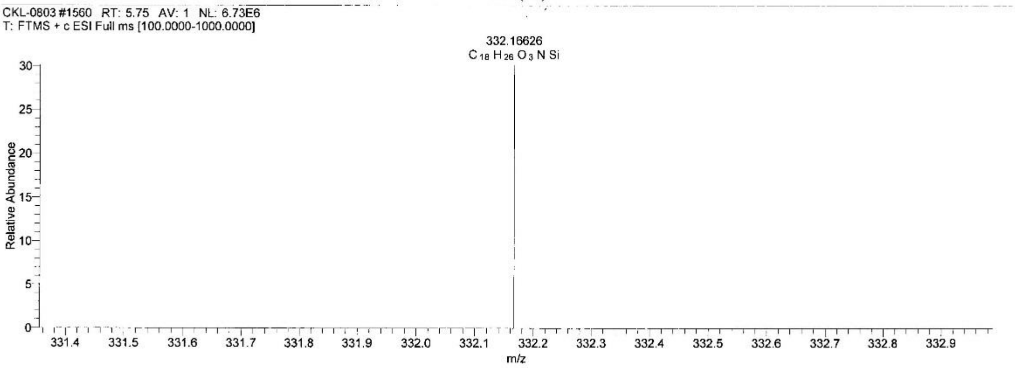

Methyl 4-phenyl-1H-pyrrole-2-carboxylate (**4a**)

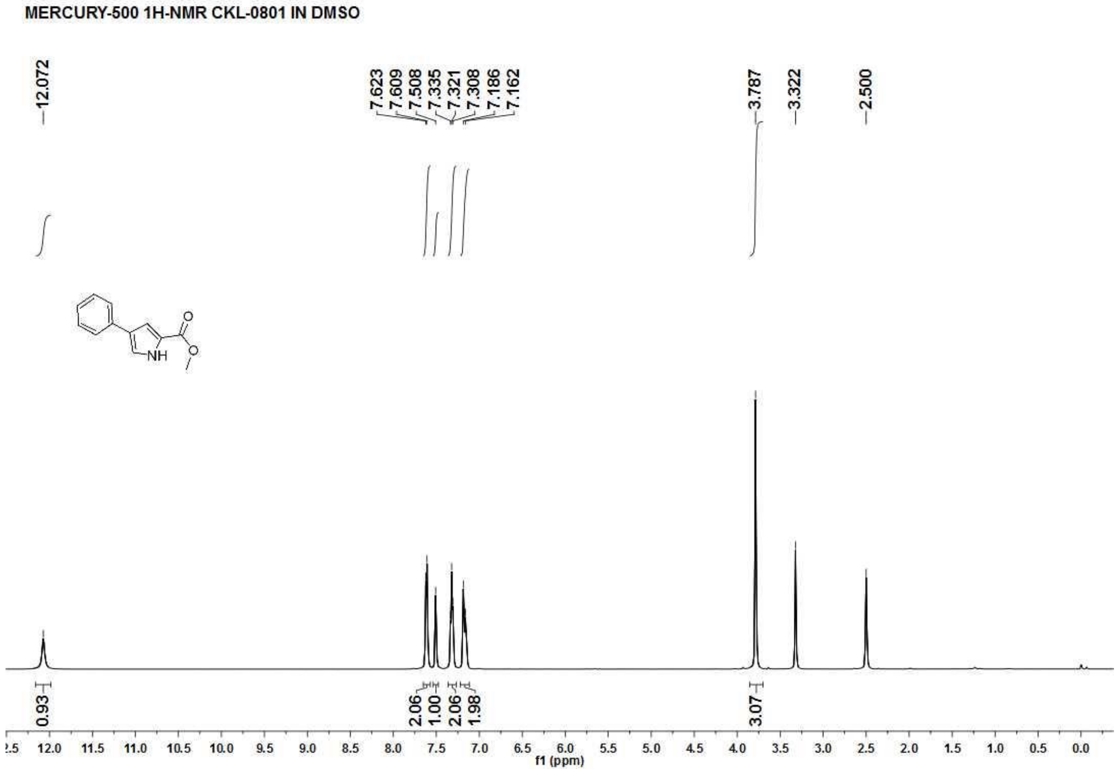

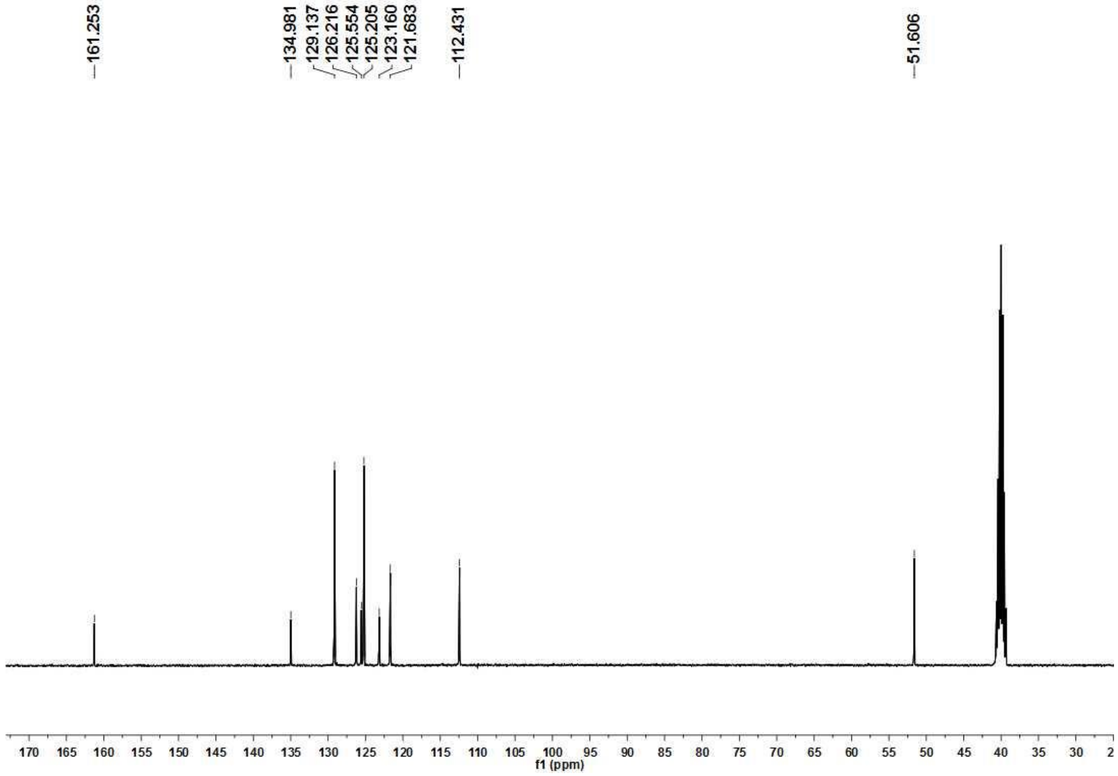

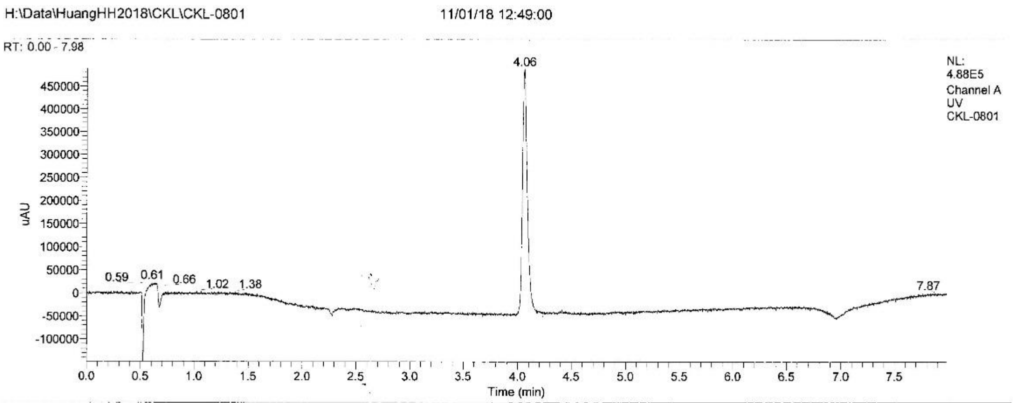

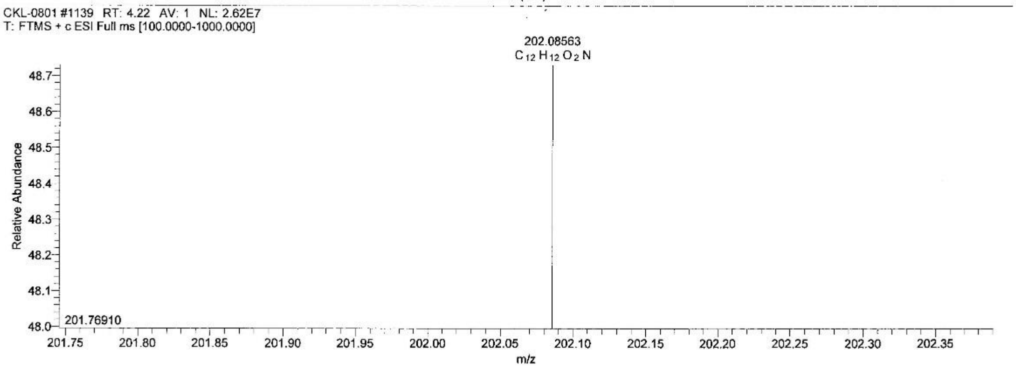

The synthesis method and yield of compound 4a in references

Synthetic route	Yield	References
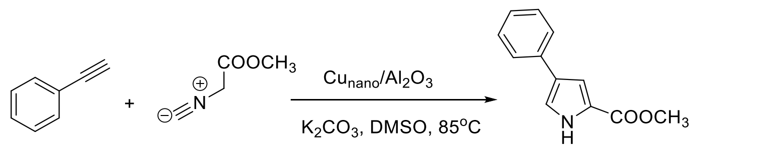	75%	*Chem. Commun.*, 2015, 51, 13646–13649
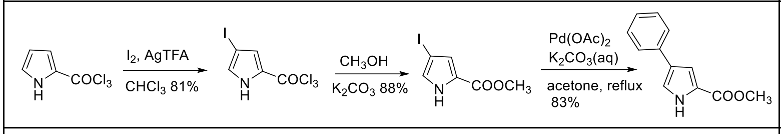	59%	*Org. Biomol. Chem.*, 2006, 4, 2477–2482
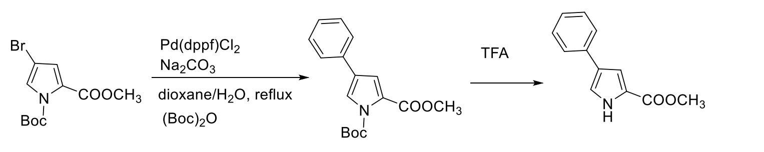	-	US2017190713
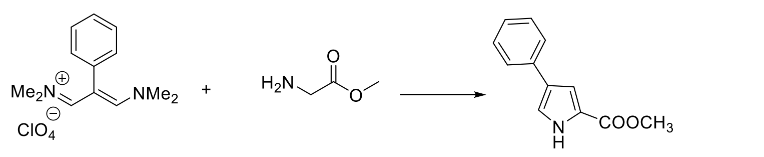	49%	*Tetrahedron Lett.*, 2010, 51, 4150–4152
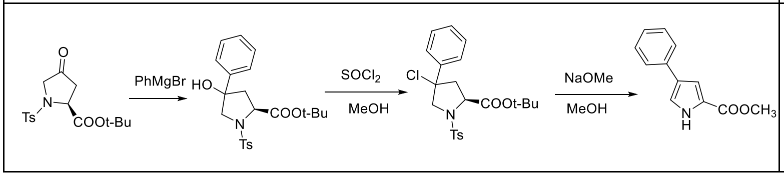	79%	*Chem. Pharm. Bull.*, 2009, 57, 167–176

4-Phenyl-1-((2-(trimethylsilyl)ethoxy)methyl)-1H-pyrrole-2-carboxylic acid (**4b**)

MERCURY-400 1H-NMR CKL-0748 IN DMSO

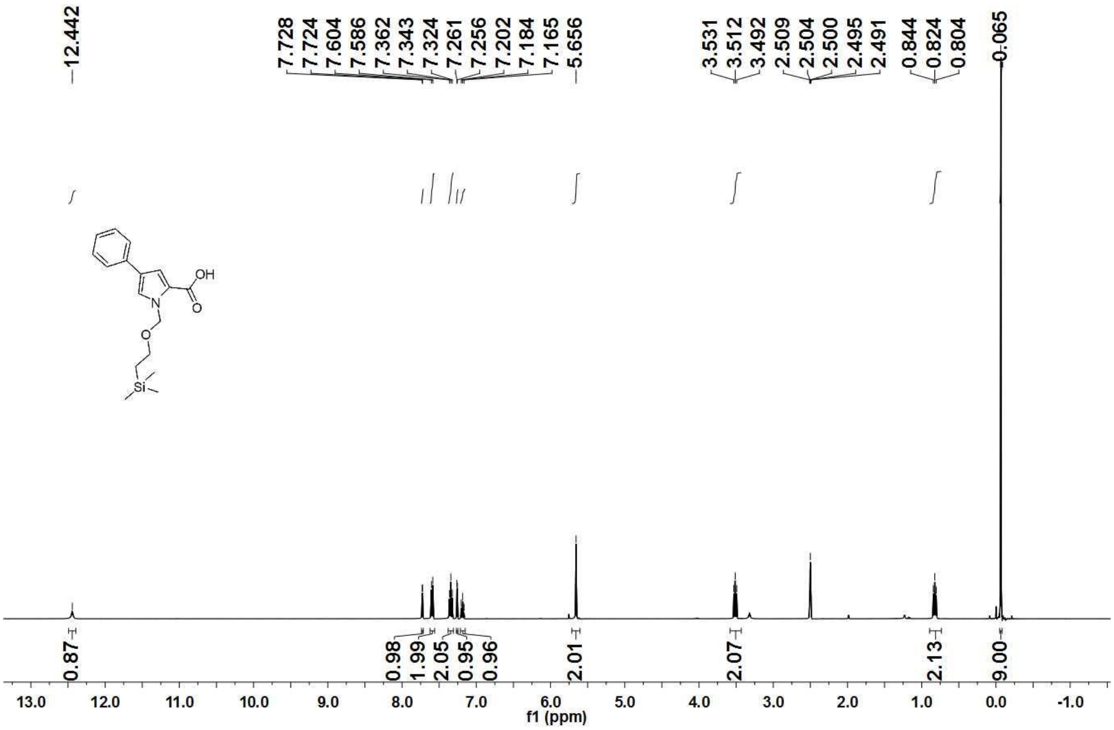

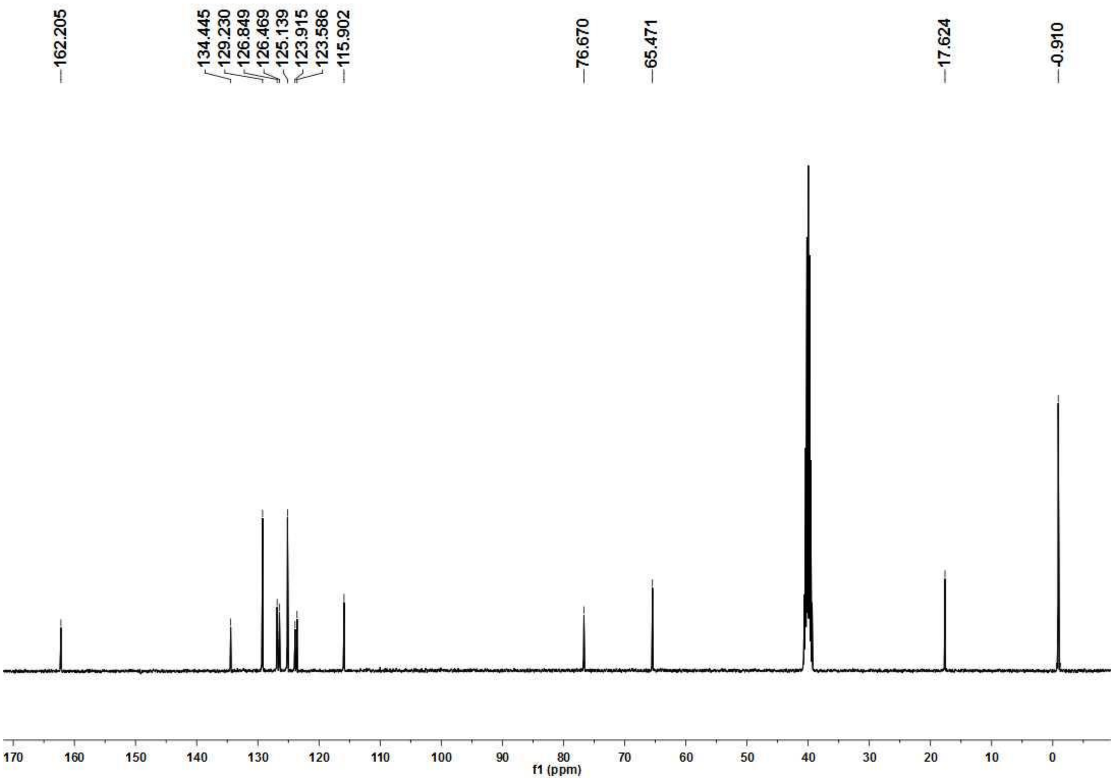

H:\Data\HuangHH2018\CKL\CKL-0748 11/01/18 12:31:54

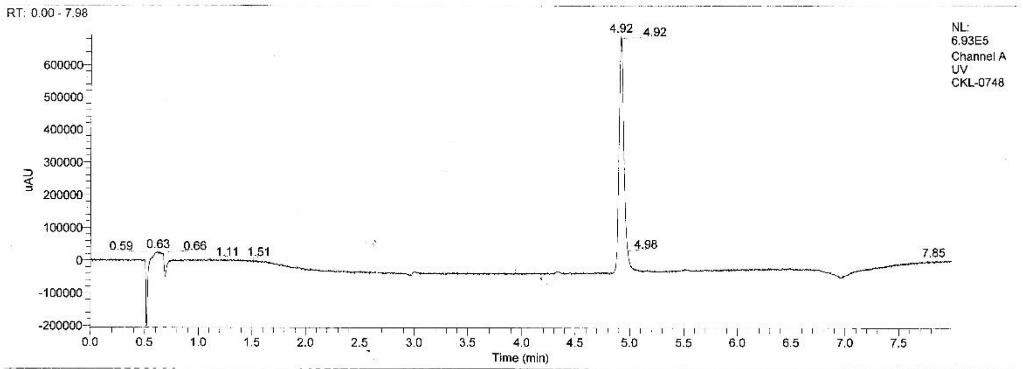

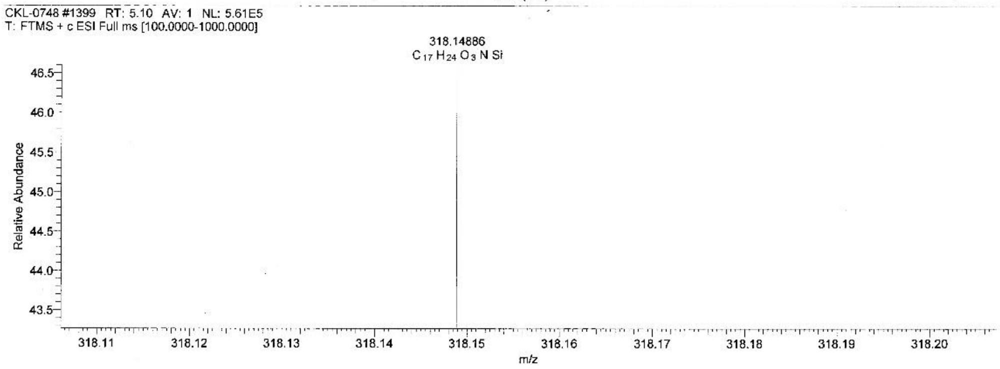

(4-Phenyl-1-((2-(trimethylsilyl)ethoxy)methyl)-1H-pyrrol-2-yl)methanol (**4c**)

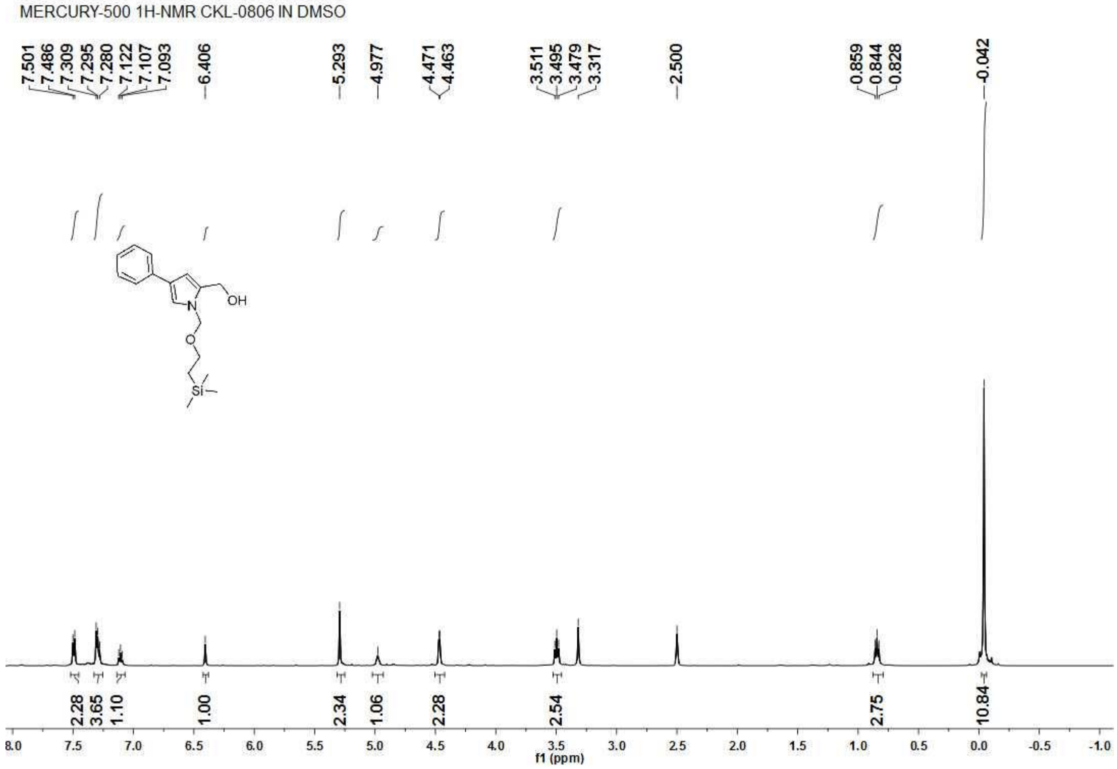

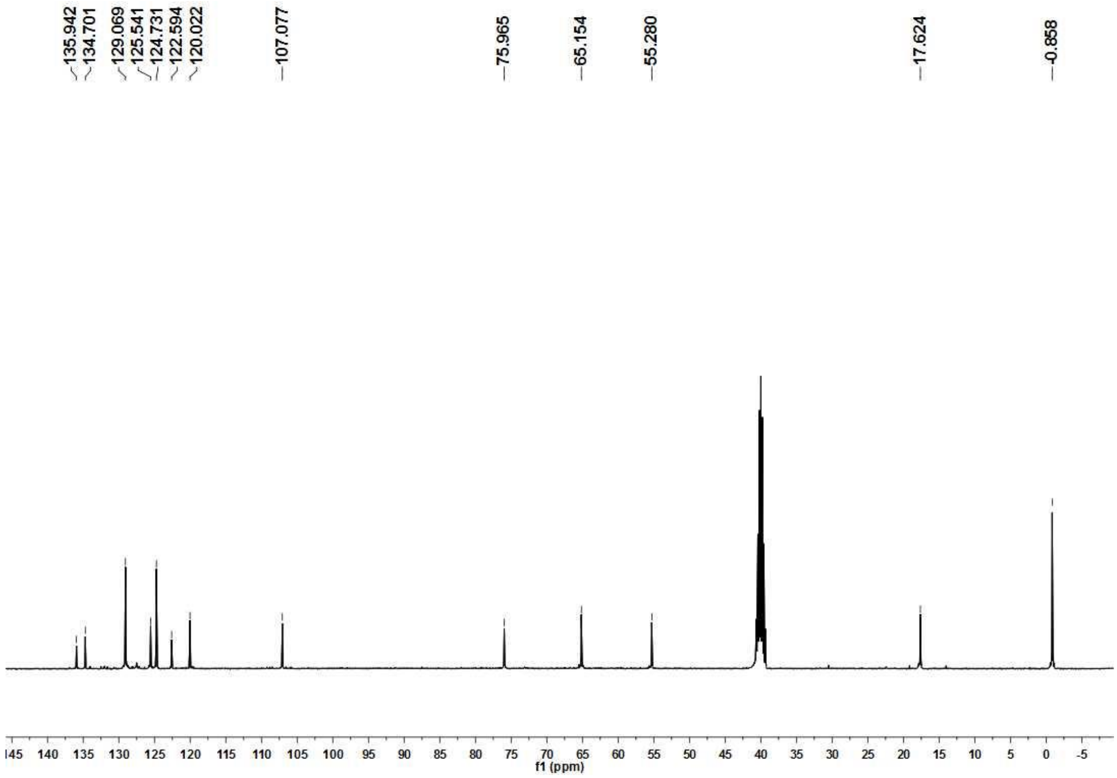

H:\Data\...\CKL\CKL-0806_181101131439 11/01/18 13:14:39

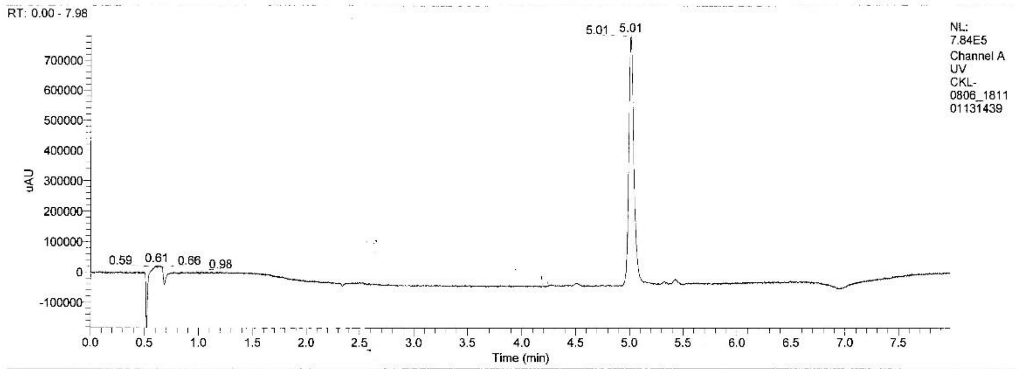

CKL-0806_181101131439 #1410 RT: 5.19 AV: 1 NL: 4.46E6
T: FTMS + c ESI Full ms [100.0000-1000.0000]

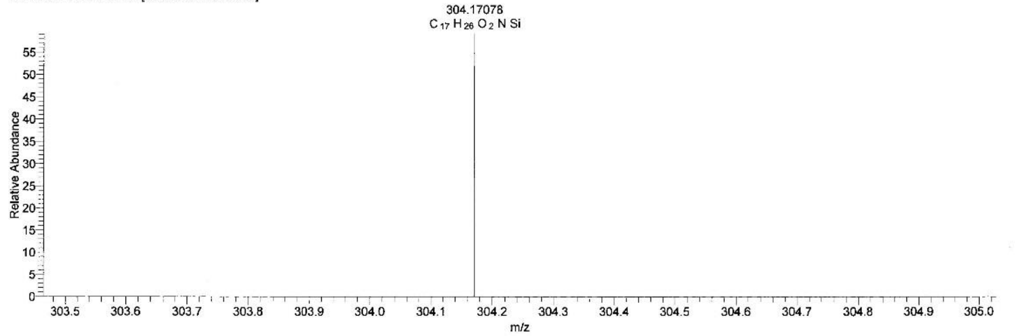

Supplement: Supplementary file 1 [file molecules-24-01594-s001.pdf]
